# Analyzing the effects of drought at different time scales on cause-specific mortality in South Africa

**DOI:** 10.1088/1748-9326/ad3bd2

**Published:** 2024-04-18

**Authors:** Coral Salvador, Raquel Nieto, Thandi Kapwata, Caradee Y Wright, Chris Reason, Luis Gimeno, Ana M Vicedo-Cabrera

**Affiliations:** 1 Centro de Investigación Marinã, Universidade de Vigo, Environmental Physics Laboratory (EPhysLab), Ourense, Spain; 2 Institute of Social and Preventive Medicine, University of Bern, Bern, Switzerland; 3 Oeschger Center for Climate Change Research, University of Bern, Bern, Switzerland; 4 Environment and Health Research Unit, South African Medical Research Council, Johannesburg 2090, South Africa; 5 Department of Environmental Health, University of Johannesburg, Johannesburg 2000, South Africa; 6 Environment and Health Research Unit, South African Medical Research Council, Pretoria 0001, South Africa; 7 Department of Geography, Geoinformatics and Meteorology, University of Pretoria, Pretoria 0001, South Africa; 8 Oceanography Department, University of Cape Town, Rondebosch 7701, South Africa

**Keywords:** drought, cause-specific mortality, vulnerability assessment, South Africa, SPEI

## Abstract

South Africa (SA) is highly vulnerable to the effects of drought on the environment, economy, and society. However, its effect on human health remains unclear. Understanding the mortality risk associated with different types of droughts in different population groups and by specific causes would help clarify the potential mechanisms involved. The study aims to comprehensively assess the effect of droughts of varying time scales on cause-specific mortality (all; infectious and parasitic; endocrine, nutritional, and metabolic; cardiovascular; respiratory) in SA (from 2009–2016) and identify more vulnerable profiles based on sex and age. We also evaluated the urbanicity and district-level socioeconomic deprivation as potential risk modifiers. We used a two-stage time-series study design, with the weekly standardized precipitation-evapotranspiration index (SPEI) calculated at 1, 6, 12, and 15 months of accumulation to identify droughts of different duration (SPEI1, 6, 12, 15, respectively). We applied a quasi-Poisson regression adjusted by mean temperature to assess the association between each type of drought and weekly mortality in all district municipalities of SA, and then pooled the estimates in a meta-regression model. We reported relative risks (RRs) for one unit increase of drought severity. Overall, we found a positive association between droughts (regardless the time scale) and all causes of death analyzed. The strongest associations were found for the drought events more prolonged (RR [95%CI]: 1.027 [1.018, 1.036] (SPEI1); 1.035 [1.021, 1.050] (SPEI6); 1.033 [1.008, 1.058] (SPEI12); 1.098 [1.068, 1.129] (SPEI15)) and respiratory mortality (RRs varied from 1.037 [1.021, 1.053] (SPEI1) to 1.189 [1.14, 1.241] (SPEI15)). An indication of greater vulnerability was found in younger adults for the shortest droughts, in older adults for medium-term and long-term droughts, and children for very long-term droughts. However, differences were not significant. Further evidence of the relevance of urbanicity and demographic and socioeconomic conditions as potential risk modifiers is needed.

## 1 Introduction

Drought is one of the most complex and destructive hazards faced by many regions worldwide, causing detrimental effects on the environment, economy, and society [1–4]. Drought generally originates from deficiencies in precipitation, known as meteorological drought, which can result in water scarcity across different parts of the hydrological cycle leading to other different types of droughts depending on the system or sector affected (e.g. agricultural, hydrological, socioeconomic drought) [5–9]. Drought can also cause adverse health effects through different pathways such as the reduction of water quantity and quality, intensification of heatwaves, increase of air pollution, reduction of food supplies, and socioeconomic disruptions, leading to an increased risk of water-, food- and vector-borne diseases, cardiovascular and respiratory conditions, mental health disorders, nutritional issues, and mortality [2, 4, 10–14]. From 1970 to 2019, drought accounted for 6% of total number of natural disasters worldwide and led to 34% of related fatalities. This impact was notably pronounced in Africa, where droughts were identified as the deadliest natural hazard [15]. Particularly in South Africa, droughts affected 414 million individuals between 1900 and 2013 and were responsible for approximately 870 000 deaths [16].

There has been growing interest in assessing the association between drought and mortality over the last few years, with studies showing inconsistent patterns. For example, a positive association was found in the Iberian Peninsula [17, 18] and urban areas of Brazil [19] for non-external, circulatory, and respiratory-cause mortality. Conversely, a slightly negative association was found in Nebraska [20] and the United States [21] for all-cause mortality. However, evidence suggests that the magnitude and direction of drought-related mortality risk can vary by regions [e.g. 22, 23], causes of death [24] and population groups [e.g. 19–21, 23]. Factors such as geography, climate, settlement patterns (rural vs. urban), demographic and socioeconomic conditions, culture, and governance can contribute to vulnerability [4, 12]. Children, the elderly, individuals living in rural areas heavily dependent on agriculture for livelihoods, and those with pre-existing conditions may face higher health risks related to extreme weather events such as droughts due to an increased susceptibility to the impacts, increased exposure and/or lack of adaptative capacity [3, 25, 26]. However, there is a lack of studies assessing the role of demographic, socioeconomic and environmental vulnerability factors as potential modifiers in drought-related morbidity and mortality risk. Contrasting findings have been emerged concerning age and sex-specific risks associated with droughts. Some studies indicated that older adults (i.e. 65 years and above) are more at risk of drought-related death than younger adults in Lisbon [18] and Brazil [19], in contrast to findings found in other studies conducted in the United States for all-cause mortality [21] and Nebraska [20]. Similarly, it is not clear whether females or males are more vulnerable to drought exposure [18–21, 23, 27]. Additionally, uncertainty exists on vulnerability according to the type of settlement [17]. The United Nations Office for Disaster Risk Reduction indicates that rural communities and farmers tend to be more vulnerable to drought effects, particularly associated with mental health [2], while other studies postulate that people living in urban areas may have higher extreme weather events-related respiratory risks because of the urban heat island effects and higher concentration of pollution from road traffic and industries in urban environments [3, 4]. However, a recent study on drought-related respiratory mortality in the United States indicated that non-metro counties were associated with a higher mortality risk ratio and were more susceptible to drought exposure than metro counties [23]. Consequently, further studies are needed to better understand the profiles of the most vulnerable populations. Additionally, it is necessary to deepen the knowledge on how different characteristics of drought events (e.g. duration, severity) influence specific health outcomes.

South Africa (SA) is a country vulnerable to drought, which has experienced significant episodes in the last decades (e.g. El Niño associated 2015/2016 drought [28], the Day Zero 2015–2018 drought in southwestern SA [29]) contributing to environmental degradation, disruption of the distribution of water resources, reduction of hydroelectrical power, agricultural losses, reduction of livestock quality, food insecurity, and economic damage [30, 31]. However, the effect of droughts on specific health outcomes remains uncertain in SA [e.g. 32]. Climate change, rapid population growth and the anticipated increase of the elderly population in SA [e.g. 16, 33] may significantly increase the exposure and vulnerability to drought in the coming decades [3, 4]. Thus, offering valuable evidence on the effects of drought on human health is crucial to guide targeted interventions to reduce the health burden associated with drought and improve the population’s resilience to the impacts of climate change in a highly vulnerable region. This study aims to determine how different types of droughts affect human health by comprehensively assessing the association between drought events of different time scales and mortality due to different causes in the total population and sex and age groups in SA between 2009 and 2016.

## 2 Material and methods

### 2.1 Mortality data

We collected daily all-cause mortality for the 52 district municipalities in SA (figure S1 in the supplementary material) between 2009 and 2016 from the National Statistics Office [34]). We excluded individuals without information on the district of residence (7.08%) and stillbirths. Additionally, we derived weekly time series of mortality for the total population and by sex, age (<5, 5–24, 25–44, 45–64, above 65 years), and cause of death by aggregating the daily counts for each week of study. In the stratified analysis, we excluded the individuals without information on sex (0.26%) and age (0.18%). We created four groups of major specific-cause mortality classified according to the 10th Revision of the International Classification of Diseases codes (www.cdc.gov/nchs/icd/icd10.htm): certain infectious and parasitic diseases (A00–B99); endocrine, nutritional, and metabolic diseases (E00–E90); circulatory diseases (I00–I99); and respiratory diseases (J00–J99).

### 2.2 Climate data

We used the Standardized Precipitation Evapotranspiration Index (SPEI) at a weekly scale which is based on a climatic water balance calculated by considering the difference between precipitation and evapotranspiration over different time scales, accounting for the influence of temperature [35].

We estimated the potential evapotranspiration applying the Hargraves method as described in Salvador *et al* [18], which requires information on the maximum and minimum temperatures and latitude of each location of study [36]. Following the World Meteorological Organization’s recommendations we used a 30 year reference period to compute the SPEI [37]. Hourly 2 m temperature and precipitation data were obtained from ERA-5 land reanalysis between 1987 and 2016 [38]. We derived the district municipality-level meteorological hourly series by averaging temperature and precipitation data from the grid cells intersecting each of the 52 district municipalities in SA. The maximum and minimum hourly temperatures were registered each day, and the mean of 24 h temperature records within each day was also considered to obtain daily series of minimum, mean, and maximum temperatures. For temperature, weekly time series were computed by averaging the daily series for each week. For precipitation, the accumulated total precipitation value registered each day was used, and the weekly time series were obtained by summing the daily records for each week of study. Regarding the latitude, we utilized the geographical coordinates of the centroid of each district municipality.

The climatic water balance was computed at one, six, twelve, and fifteen months of accumulation (SPEI1, 6, 12, 15, respectively) and these series were independently adjusted using a log-logistic probability distribution and transformed into a standard normal variable, which makes the SPEI a drought index spatially and temporarily comparable [35]. Thus, negative values of SPEI correspond to drought conditions and positive values of SPEI correspond to wet conditions. Meanwhile, the different time scales for which the SPEI was calculated represented periods of time over which deficits/surplus are accumulated, allowing us to characterize different types of droughts and wet events. For example, meteorological drought is usually better represented by shorter time scales (1–3 months; short-term droughts), agricultural drought is usually better represented by 3–6 months (medium-term droughts); and hydrological drought is usually better represented by longer time scales (e.g. longer than 9–12 months; prolonged droughts) [20, 39, 40]. Additionally, the severity of droughts is represented by the value of the SPEI on the negative scale, with lower values indicating higher severity of the events. Since we worked at weekly time scale, SPEI series contained a specific value for each week (four values per month), where 1 month SPEI deficit corresponded to the accumulation of deficit over a region for the last four weeks period (1 month); 6 month SPEI deficit for the last 24 weeks; 12 month SPEI deficit for the last 48 weeks; and 15 month SPEI deficit for the last 60 weeks, respectively.

### 2.3 Main analysis

**Figure 1:**
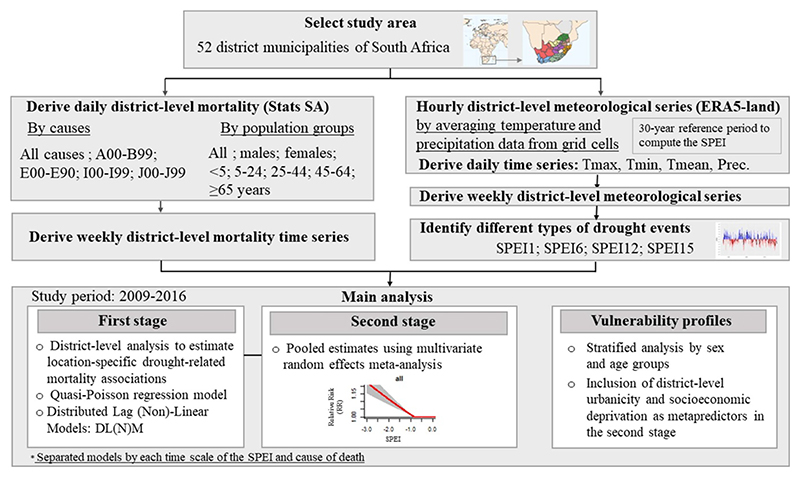
Diagram of the design and procedures conducted in this research study. Tmax: maximum temperature registered each day; Tmin: minimum temperature registered each day; Tmean: mean 24-hourly temperature within each day; Prec.: total precipitation accumulated each day. SPEI: the Standardized Precipitation Evapotranspiration Index calculated at 1, 6, 12, and 15 months of accumulation (SPEI1; SPEI6; SPEI12; SPEI15, respectively). A00–B99: mortality due to certain infectious and parasitic diseases; E00–E90: mortality due to endocrine, nutritional, and metabolic diseases; I00–I99: mortality due to circulatory diseases; J00–J99: mortality due to respiratory diseases.

We conducted a two-stage time-series analysis to assess the association between droughts and weekly mortality in SA (figure 1).

In the first stage, we applied a quasi-Poisson regression model for each district municipality and SPEI index separately to obtain location-specific estimates (method S1 in the supplementary material). We constructed independent models for each cause of death and population group, and the stratified analysis by sex and age was restricted to all-cause mortality due to the low statistical power in specific-cause mortality. In this analysis we used continuous weekly SPEI series. Given our focus on droughts, and following the methodology used in a recent study [19], we applied a threshold function to model the exposure-response association assuming a linear association for negative values of the SPEI, using as the reference value SPEI = −0.84. This threshold was chosen based on the classification system proposed by Agnew [41] to identify droughts, which has been widely applied in other studies [e.g. 39, 42–44] indicating that values ranging from 0 to −0.84 typically represent ‘normal’ or ‘near normal’ conditions. Then, the relative risks (RRs) were estimated for each unit of increase in drought severity (represented by the negative scale of the SPEI) for the same week’s exposure. We adjusted the model by mean weekly temperature with a distributed lag non-linear model (DLNM) to flexibly describe in a bi-dimensional space of functions simultaneously the non-linear shape of the exposure-response association and the lag dimension to account for the delayed effect of temperature on mortality [45]. The temperature-mortality association was modeled using a cubic spline with three internal knots at the 10th, 75th, and 90th percentiles of each location-specific mean temperature, following the specifications used in a recent study on temperature-related mortality in SA [46]. We modeled the lag-response association using an unconstrained lag structure up to 3 weeks of lag as in Salvador *et al* [19]. We also controlled for seasonality and long-term trends in the main model. For that, we conducted a battery of tests exploring different specifications to ensure proper control of temporal patterns by each subgroup of study and the time scale of the SPEI. Specifications were chosen based on the quasi-Akaike information criteria (see table S1 in the supplementary material). For most studied subgroups, including the all-cause, we used a natural spline of time with two degrees of freedom per year and a natural spline with two degrees of freedom of the week within the year (‘week’). An additional degree of freedom was included in the latter for younger adults (25–44 years) using SPEI6, SPEI12 and SPEI15. Another additional degree of freedom was included when exploring drought effects on respiratory mortality. Among children, temporal patterns were controlled using a natural spline of time with three degrees of freedom per year.

In the second stage, we applied meta-analytical models to combine individual district-specific estimates and derive the pooled drought-mortality association in SA. The models included as meta-predictors the temperature range by district. We conducted separate meta-analytical models for each time scale of the SPEI, cause of death, and population group. Additionally, we explored the role of the district-level urbanicity and socioeconomic deprivation as meta-predictors of the overall association to evaluate whether potential risk differences across population groups may be explained by these factors. Rural and urban areas often differ in infrastructure, population density and dynamics, access to resources, healthcare services, dependency on agriculture-based livelihoods, and environmental conditions (e.g. heat island effects, air pollution levels, availability of green spaces) [47], which may contribute to differences in drought-related health risks across urban and rural populations. Socioeconomic deprivation status may intensify climate-related health risks associated with higher exposure, higher susceptibility (e.g. reduced health status) and/or lower adaptative behavior due to limited education, reduced access and control of resources, illiteracy, lower awareness of climate-related health risks, among others [26]. In this analysis, we used a district-level multidimensional poverty index in SA as a continuous variable using information in 2011 Census. That index accounts for 12 indicators capturing deprivation for four dimensions: education (years of schooling and school attendance), health (child mortality, disability), standard of living (cooking fuel, water, sanitation type, dwelling type, refuse removal frequency, asset ownership, overcrowding), and economic activity (unemployment) [48]. Table S2 shows the classification of the 52 district municipalities according to urbanicity level and poverty.

We conducted the analysis using R software (version 4.2.2) and the *SPEI, dlnm*, and *mixmeta* packages.

## 3 Results

**Figure 2:**
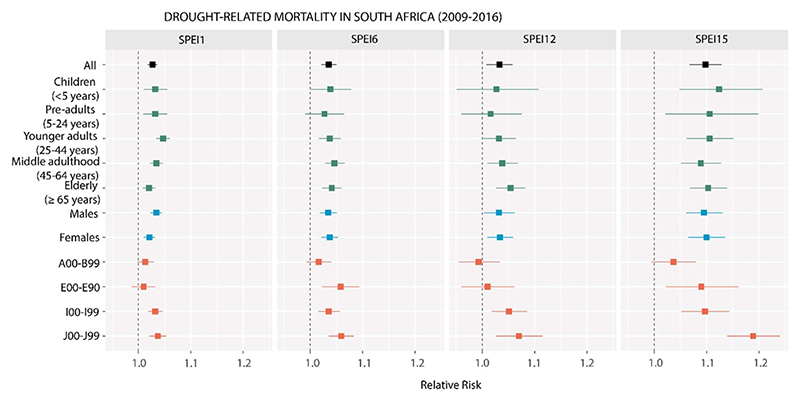
Pooled results for drought-mortality associations categorized by groups of sex, age and causes of death (relative risk, RR) using various drought metrics to characterize short, medium, long, and very long term (SPEI1, 6, 12, 15, respectively) in South Africa between 2009 and 2016. Estimates were calculated for one unit increase of drought severity. Horizontal bars correspond to the 95% confidence intervals. All: all-cause mortality; A00–B99: mortality due to certain parasitic and infectious diseases; E00–E90: mortality due to endocrine, nutritional, and metabolic diseases; I00–I99: mortality due to circulatory diseases; J00–J99: mortality due to respiratory diseases.

A total of 3757 736 deaths were assessed across all district municipalities of SA between 2009 and 2016. Tables S3 and S4 provide a summary of the descriptive statistics regarding the exposures and mortality outcomes used in this study, overall and for each district municipality of SA. We found that drought events increased the risk of all-cause mortality in all population groups, regardless of the SPEI time scale used (figure 2; table S5). Larger risks were predominantly associated with very long-term drought conditions (RR[95%CI]: 1.098 [1.068, 1.129] using the SPEI15), compared to shorter droughts (i.e. 1.027 [1.018, 1.036] for SPEI1; 1.035[1.021, 1.050] for SPEI6; 1.033 [1.008, 1.058] for SPEI12). We also observed a positive association for specific causes (figure 2, table S5). In particular, the risks seem to be larger for respiratory mortality, which tended to be even higher as droughts were more prolonged (e.g. 1.037 [1.021, 1.053] for SPEI1; 1.189 [1.14, 1.241] for SPEI15). A similar pattern was observed for circulatory mortality (e.g. 1.032 [1.019, 1.046] for SPEI1; 1.097 [1.052, 1.144] for SPEI15, respectively). The association between drought and mortality due to certain infectious and parasitic diseases was positive, but also imprecise, for short- (1.013 [0.998, 1.029]), medium- (1.016 [0.993, 1.04]), and very long-term droughts (1.037 [0.995, 1.08]). For mortality due to endocrine, metabolic, and nutritional causes, we found a positive association independently of the time scale of the SPEI used, which was only robust for medium-term (1.058 [1.023, 1.094] for SPEI6) and the longest drought conditions analyzed (1.09 [1.023, 1.161] for SPEI15).

Droughts significantly increased the risk of mortality in all population groups by sex and age, with no substantial differences between them. However, we observe interesting patterns. For example, for the shortest drought conditions, we found larger risks in younger adults (i.e. 25–44 years) compared to the oldest ones (⩾65 years) (1.047 [1.034, 1.06] *vs* 1.02 [1.008, 1.032], using the SPEI1), while for medium- and long-term droughts (SPEI3, SPEI12), older population groups reported higher risks than younger adults and preadults (e.g. 1.046 [1.028, 1.066] in people in middle-adulthood (45–64 years) *vs* 1.027 [0.99, 1.065] in pre-adults using the SPEI6; or 1.054 [1.027, 1.083] in the oldest population group *vs* 1.016 [0.96, 1.075] in pre-adults using the SPEI12). However, children under 5 years were mostly affected by very long-term droughts, with the highest risk reported for all causes in SPEI15 among all age groups (1.124 [1.048, 1.207]). However, there were almost no differences in the estimates obtained for different sex groups, and only for shorter droughts we observed a slightly larger risk in males compared to females (1.034 [1.023, 1.045] *vs* 1.021 [1.01, 1.031]) (figure 2, table S5).

Additionally, we did not find substantial differences in drought-related mortality risk across different district- level urbanicity and deprivation over-all (*p* > 0.05 for these predictors in the metanalytical model; table S6 shows the coefficients, relative risks, 95% confidence intervals, and *p*-values). However, we observed some exceptions across specific population groups associated with prolonged droughts. For example, urban and other people aged 5–24 years were associated with lower risks compared to their rural counterparts during very long-term droughts (a negative and significant coefficient (*p* < 0.05) was found for ‘urban’ and ‘other’ categories compared to the ‘rural’ category). Also, women and the elderly residing in more deprived districts were associated with higher risks compared to women and the elderly residing in less deprived areas during very long- and long-term droughts, respectively (a positive and significant coefficient was identified, indicating that the greater the deprivation the greater risk).

**Figure 3:**
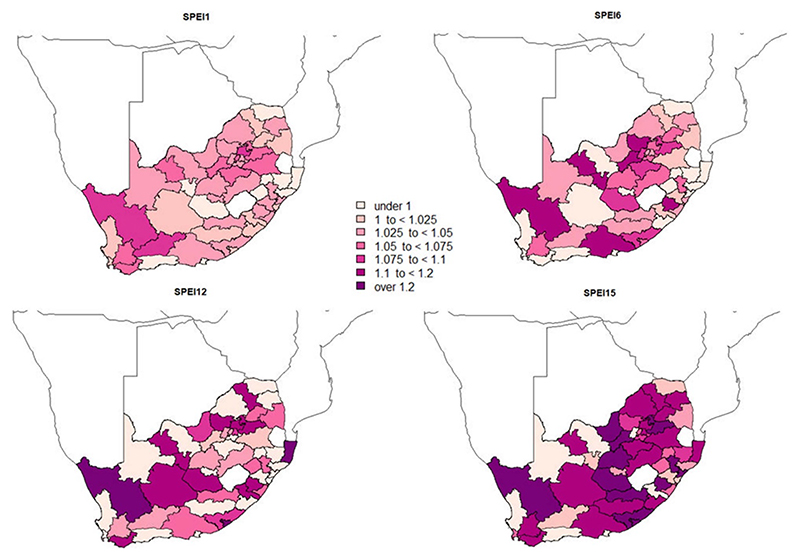
Map risk (RR, 95%CI) of all-cause mortality associated with drought measured by the weekly SPEI calculated at different time scales (1, 6, 12, 15 accumulation months; SPEI1, 6, 12, 15) in the total population at district municipality level in South Africa between 2009 and 2016 (represented in a color gradient).

Figure 3 shows the relative risks of all-cause mortality associated with different types of droughts of different time scales across the 52 district municipalities in the total population of SA. Tables S7–S10 in the supplementary material show all estimates, including for specific causes of death and population groups. For most district municipalities, the longest drought events (SPEI15) were associated with a larger and more robust estimation of all-cause mortality risk in the total population compared to shorter scales of the SPEI. Additionally, the most extended droughts seemed to mainly affect all-cause mortality in the Eastern half of the country (figure 3, tables S7–S10). We also found negative associations in some locations; however, those estimates were highly uncertain. The role of frequency and severity in drought-related mortality risks was not clear since inconsistent patterns were observed across the 52 district municipalities (i.e. higher risks were found both in locations where droughts were highly frequent and/or severe and in locations where the number and magnitude of the events was relatively low) (tables S4, S7–S10). Further details in the supplementary material.

## 4 Discussion

This is the first nationwide epidemiological study assessing the association between droughts of varying time scales, population groups, and cause-specific mortality in SA. Our findings provide strong evidence indicating that drought events (regardless of the accumulation period) increased the risk of all-cause mortality and for specific causes, mainly for most extended periods of drought analyzed and due to respiratory causes. Only estimates obtained with the SPEI15 were substantially different to those obtained with the rest of the drought indicators. Our findings also suggest that patterns of vulnerability may differ across different population groups and types of droughts, with factors such as urbanicity and district-level deprivation being particularly relevant for certain groups of individuals during pro-longed droughts, making them more vulnerable to the effects.

An increased risk of mortality was also associated with drought events in several regions around the world including peninsular Spain [17], Lisbon [18], urban areas of Brazil [19]; and Bangladesh [24, 49]. Also, Berman *et al* [50] indicated that worsening droughts were associated with higher rate of mortality in older people, especially in regions where droughts were less frequent. However, there is a limited number of studies that evaluate the influence of the time scale of droughts on mortality risk, which makes the comparison across studies difficult. In line with our results, Salvador *et al* [22] suggested an indication of higher risks related to a longer accumulation period (SPEI3) compared to the shortest one (SPEI1) in peninsular Spain, especially for respiratory mortality. However, other studies suggested a higher negative influence on mortality risk in shorter droughts (3 months of accumulation) compared to longer droughts (12 months of accumulation) in northern Bangladesh [24, 49]. Thus, further evidence is needed to clarify existing uncertainties. It is worth noting that these studies showed heterogeneity across regions and did not account for the same time scales than those used in this study (e.g. more extended droughts). Different characterization of drought events, as well as the use of different drought indices, may also influence drought-related mortality [2, 4, 23, 24]. Meanwhile, differences found in this study across district municipalities may be explained by potential differences in the drought exposure as well as in the adaptative capacity and resilience of the populations (e.g. more efficient early warning systems, availability and access to public health services and interventions or better preparation to mitigate drought effects) [4, 23].

Overall, in our study, droughts were mostly associated with respiratory mortality. Specifically, only respiratory and circulatory deaths showed robust associations with drought events for all time scales analyzed, with risks increasing as the accumulation period of the drought events extended. These findings are consistent with previous studies conducted in Spain, which have shown that drought conditions are more strongly associated with respiratory mortality compared to circulatory mortality [e.g. 17, 22]. Different mechanisms can be involved such as the intensification of extreme heat events via land-atmospheric feedbacks [51], and reduction of air quality [6, 17]. Droughts are often associated with stagnant conditions, which are frequently linked to the presence of persistent high-pressure systems, leading to a higher concentration of air pollutants [8]. Also, as water becomes scarce and the soil becomes drier during a drought episode, the amount of dust and other toxic particles suspended in the air (including smoke from wildfires) can increase [1, 4, 52], which lead to the irritation of the respiratory system and trigger local and systemic inflammatory and oxidative processes, resulting in detrimental effects on respiratory and cardiovascular health [23, 39, 53–56]. Droughts can also lead to the proliferation of air-borne allergens such as pollen, exacerbating allergies [23]. The fact that we observed larger drought effects on respiratory and circulatory mortality for longer drought events compared to the shorter ones may be partly related to the hypothesis that longer droughts can aggravate the effects previously mentioned and may be more likely to be associated with an increased risk of other environmental hazards such as wildfires and air pollution (especially when droughts are synchronized with heatwaves [11]). Wildfires are in turn associated with a wide range of health outcomes, including respiratory and circulatory conditions [4, 57].

Our results also indicate that droughts were associated with an increased risk of mortality due to endocrine, nutritional, and metabolic causes in SA, in particular for medium-term droughts (which are generally associated with agricultural impacts induced by drought), and the most extended periods of drought analyzed. In this regard, longer droughts generally affect food production (e.g. reduced sowing and crop yields, decreased livestock) resulting in reduced availability, access, and intake of food. This can compromise diet quality and increase the risk of hunger and malnutrition [4, 58]. In severe cases, prolonged droughts can trigger severe famines and increase the risk of death, particularly among vulnerable populations such as rural communities, children, pregnant women, the elderly, and socioeconomically deprived populations [55, 58]. Furthermore, the impacts of drought on economic sectors and food insecurity can lead to economic disruptions and contribute to increased food prices [2, 30, 55, 59]. Vulnerable people such as poor individuals or those living in developing countries can be more forced to consume less diverse and balanced diets [58, 60], which increase the risk of endocrine, metabolic, and/or nutritional disorders [61]. Droughts have been also associated with deficiencies of essential micronutrients such as vitamins and minerals (e.g. vitamins A and C, iron, folic acid, zinc) [58, 62], which play an important role in metabolism and the maintenance of the correct functioning of the tissues [62, 63]. Evidence also indicates that exposure to undernutrition or famine during childhood can have long-lasting negative effects on an individual’s physical and cognitive development in later life [4] and increase the risk of disease [64].

In this study, we also found a positive but highly imprecise association between droughts and mortality due to certain infectious and parasitic diseases for short-, medium-, and very long-term drought conditions, while for long-term droughts we found a null risk. Another study conducted in Northern Bangladesh reported a positive and robust association between shorter droughts (3 months of accumulation) and infectious disease-related mortality in some regions, but not for long-term droughts (12 months of accumulation) [24]. In this context, droughts have been associated with a decrease in water quantity and quality [65], which can threaten human health [1, 2, 6, 55]. This, combined with the lack of hygiene purposes, can contribute to a higher risk of water-borne infectious diseases caused by parasites and bacteria, leading to multiple gastrointestinal disorders, diarrhea [66], and, in the worst scenario, an increased risk of death is also possible. Drought has also been associated with changes in the transmission of certain vector-borne diseases such as dengue, malaria, chikungunya, or West Nile Virus through different pathways [2, 4, 14, 55, 67]. In addition, drought-related nutritional effects can also contribute to an increased risk of infectious diseases by weakening people’s immune systems [68]. Some studies indicate that undernutrition is a risk factor for infectious and respiratory diseases, which are among the leading causes of death in children [62, 69]. Also, droughts can alter the distribution and abundance of certain animal and insect species, which can influence the likelihood of disease transmission between wildlife and humans [52, 53].

It should be noted that drought-related stressors such as economic pressures can also lead to mental health disorders, including chronic stress and anxiety, and, in extreme cases, increase the risk of suicide [4, 6, 24, 55, 70]. Evidence indicates that chronic stress can contribute to a higher risk of disease and premature mortality by affecting the neuro-immuno-endocrine axis and disturbing the body’s homeostasis, which has adverse effects on multiple physiological functions including growth, metabolism, inflammatory and endocrine functions, and immune competence [6, 56, 71, 72].

We did not find clear evidence of different vulnerabilities across sex and age groups; however, some interesting patterns were observed. Specifically, medium-term, and long-term drought conditions tended to be associated with a slightly higher risk of death in older adults aged 45–64 and 65 and above, respectively, while the shortest droughts seemed to have a slightly higher effect on young adults (25–44 years). However, in the case of the most pro-longed droughts analyzed, our study suggested an indication of a larger vulnerability in small children, where the risk of mortality due to all specific causes of death was particularly elevated. These diverse risk patterns could be influenced by differences in drought exposure and the susceptibility and adaptative capacity of the individuals within each population group [2, 4]. Prior studies indicate that people aged 65 years above are more vulnerable to extreme climate-related events such as drought due to their higher prevalence of chronic diseases and comorbidity, diminished homeostasis capacity, and their tendency to live alone, isolated, and with reduced financial status [3, 19, 73]. Additionally, other studies speculate that younger adults may be more at risk of drought-related mortality due to a higher level of exposure during drought events [20]. For instance, we found slightly larger risks in middle-aged adults associated with medium-term drought conditions (i.e. agricultural droughts). This might imply that this particular age group may be more involved in outdoor activities, potentially related to farming and livestock, leading to increased exposure and risk to their health. However, further analyses are required to test this hypothesis. Meanwhile, several studies suggest that children are particularly susceptible to drought-related nutritional effects (as they heavily rely on adequate food intake for their development and growth) and to drought-related infectious diseases [61]. In a recent study conducted in Uganda, it was found that drought reduced the supply of calories, protein, and zinc resulting in lower heigh-for-age-*z*-scores and higher risk of stunting in children aged 6–59 months. Additionally, that study found a reduction in diet diversity associated with drought conditions [69]. A recent comprehensive study also suggested a positive and robust association between longer droughts and diarrhea risk in children under 5 years from 51 low-and middle-income countries, which was larger for children living in households that required more time to collect water or had limited access to water or soap/detergent for handwashing [66]. Furthermore, children are also considered vulnerable to the effects of drought-related environmental hazards such as air pollution events because they have an increased physiological susceptibility, an immature immune system and tend to be more exposed [19, 56]. According to the role of urbanicity factor, this study suggested that rural people aged 5–24 years were more vulnerable to their urban counterparts within the same age range during very long-term droughts. One hypothesis that may explain this finding may involve that young and less experienced farmers, particularly those in rural areas, often face higher levels of stress during droughts compared to older adults [55]. We also noted that females and the elderly residing in areas of higher deprivation were more vulnerable to their counterparts living in lower deprived areas during prolonged droughts (SPEI15, SPEI12, respectively). They may be more likely to be more exposed, have poorer access to clean water and sanitation and limitation of preventive and adaptative resources. It is worth noting that the elderly are highly susceptible to health impacts attributable to extreme weather phenomena, and socioeconomic status may disproportionately amplify their vulnerability. However, finer analysis (e.g. at the individual level) is needed to clarify the role of socioeconomic deprivation factors as modifiers of drought-related health risks.

In contrast, we did not find significant differences across sex groups, similar to previous studies that indicate that sex did not seem to have a relevant role in vulnerability to drought-related mortality [e.g. 21]. However, our findings are not consistent with those found in other studies [e.g. 18, 19, 27]. It should be noted that estimates were highly heterogeneous across different district municipalities, with larger risks in females in some locations, and opposite patterns or no substantial differences in others. As Salvador *et al* [19] speculated, potential local factors (e.g. availability of services focused on supporting vulnerable gender groups, different levels of exposure between males and females, differences in gender roles, occupation, education, or economic level as well as in environmental awareness, preparedness, and capacity of adaptation) may influence differences found across district municipalities. However, further assessments are needed to clarify the influence of local factors on drought-related mortality risks between both sex groups at the district municipality level. It is also worth noting that we only conducted a stratified analysis by sex and age accounting for all-cause mortality, and we may expect larger differences across the population groups by specific causes of death, as argued in previous studies [e.g. 18, 20, 62, 74].

This study has important strengths that need to be highlighted. First, to our knowledge, it is the first nationwide assessment of different types of drought events of varying time scales on specific mortality outcomes and population groups. We here provide useful information for the management of the current and future health impacts associated with drought events in a country highly affected by this extreme weather phenomenon. In addition, the analysis was conducted using a robust study design and model, that allowed us to accurately control for the effect of temperature. Furthermore, we used a robust and standard high resolution drought index to monitor drought events [75]. The SPEI has been extensively used in scientific literature, including epidemiological studies on drought and health [19, 22, 66]. Additionally, the study tested the role of urbanicity and district-level socioeconomic deprivation as risk modifiers. However, it is important to acknowledge some limitations of this study. This comprehensive research was based on weekly aggregated data at the district municipality level, so results cannot be extrapolated at the individual level. Moreover, due to statistical power issues, we could not include a stratified analysis by specific causes of death for the different population groups. It is required further availability of environmental and climatic data including variables related to agricultural activities, water availability and quality, and air quality, to conduct finer research on the contribution of potential key mediators and modifiers in the drought-health association.

## 5 Conclusions

This study shows robust evidence of an increased risk of all-cause mortality and specific causes (infectious and parasitic; endocrine, nutritional, and metabolic; circulatory; respiratory) associated with drought events in SA. Our findings indicate that the effect of drought can vary depending on the accumulation period of the events and causes of death, with larger risks being associated with more extended droughts and respiratory mortality. Additionally, risk patterns may vary across population groups. These results are of particular relevance in SA because it is a country prone to drought episodes and highly vulnerable to climate change effects.

Therefore, this study offers valuable information that can be included as recommendations in public health interventions to reduce the health burden associated with droughts and enhance preparation, awareness, and concern among the population. Strategies for managing droughts should focus on proactive approaches, emphasizing interventions that strengthen structures dedicated to drought prevention and impact minimization instead of short-term reactive crisis responses, to provide sustainable solutions. Critical steps include improving monitoring and early warning systems, investing in research, reinforcing policies for land and water use regulations, and other actions by policy- and decision-makers aimed to increase preparedness and adaptative capacity, especially in vulnerable populations. It is imperative to ensure funding for capacity building, education, training, public awareness campaigns, and reinforcement of health infrastructure and services [4, 10].

Also, it is crucial to enhance multi-stakeholder participation and planning across different sectors and geographical levels, developing integrated drought risk management plans with the collaborative effort of countries’ governments, national and local authorities, private stakeholders, academia, and community organizations [4, 10]. In this context, further research is needed on vulnerabilities and impact assessments to guide targeting interventions aimed to increase the resilience of the population of SA. Future research includes the design of studies focused on highly vulnerable groups to better understand specific mechanisms and risk factors and combine quantitative analysis with qualitative methods (e.g., interviews, focus group discussions) to gain a deeper understanding of how droughts impact vulnerable communities, including behavioral adaptations and barriers to care.

## Supplementary Material

Supplementary material

## Data Availability

Climatic series were obtained from the European Centre for Medium-Range Weather Forecasts public datasets (ECMWF): https://doi.org/10.24381/CDS.E2161BAC. Mortality data was available after signing the data agreement with the Statistics South Africa. The data cannot be made publicly available upon publication because they contain sensitive personal information. The data that support the findings of this study are available upon reasonable request from the authors.
