# Supplementary material for: Analyzing the effects of drought at different time scales on cause-specific mortality in South Africa
Source: Environ Res Lett. Author manuscript; Available in PMC 2024 Jun 7. (PMC7616071; doi:10.1088/1748-9326/ad3bd2)
Supplement: Supplementary material [file EMS196490-supplement-Supplementary_material.docx]

Supporting Information for

**ANALYZING THE EFFECTS OF DROUGHT AT DIFFERENT TIME SCALES ON CAUSE-SPECIFIC MORTALITY IN SOUTH AFICA**

Coral Salvador^1,2,3^, Raquel Nieto^1^, Thandi Kapwata^4,5^, Caradee Y Wright^6,7^, Chris Reason^8^, Luis Gimeno^1^, Ana M. Vicedo-Cabrera^2,3^

*^1^ Centro de Investigación Mariña, Universidade de Vigo, Environmental Physics Laboratory (EPhysLab), Ourense, Spain.*

*^2^ Institute of Social and Preventive Medicine, University of Bern, Bern, Switzerland.*

*^3^ Oeschger Center for Climate Change Research, University of Bern, Bern, Switzerland.*

*^4^ Environment and Health Research Unit, South African Medical Research Council, Johannesburg 2090, South Africa.*

*^5^ Department of Environmental Health, University of Johannesburg, Johannesburg 2000, South Africa.*

*^6^ Environment and Health Research Unit, South African Medical Research Council, Pretoria 0001, South Africa.*

*^7^ Department of Geography, Geoinformatics and Meteorology, University of Pretoria, Pretoria 0001, South Africa.*

*^8^ Oceanography Dept, University of Cape Town, Rondebosch, 7701 South Africa.*

**Corresponding author:** Coral Salvador

Email: csalvador@uvigo.gal

**Table of Contents**

**Supplementary figures**

**Figure S1.** Study region showing the 52 district municipalities of South Africa, grouped into 9 provinces (colours) within the broader Southern African region.

**Supplementary methods**

**Method S1.** Quasi-Poisson regression model to estimate the effects of drought on weekly mortality in each district municipality of South Africa between 2009 and 2016.

**Supplementary tables**

**Table S1**. Quasi-Akaike information of models with different specifications to control the seasonality and long-term trend.

**Table S2.** Classification of the 52 district municipalities of South Africa according to the district-level urbanicity and socioeconomic deprivation.

**Table S3**. Descriptive statistics corresponding to weekly counts of all-cause mortality and specific causes of the total population (A00-B99= certain parasitic and infectious diseases; E00-E90= nutritional, endocrine and metabolic diseases; I00-I99= circulatory diseases; J00-J99= respiratory diseases) for each district municipality of South Africa between 2009 and 2016. Total number of all-cause deaths separated by sex (males, females) and age (<5, 5-24, 25-44, 45-64, ≥65 years) groups was also reported.

**Table S4**. Descriptive statistics corresponding to drought conditions measured by weekly SPEI calculated at short-, medium-, long-, and very long- term (SPEI1,6,12,15, respectively) and weekly mean temperature for each district municipality and province of South Africa and overall, between 2009 and 2016. D= number of weeks with drought; I: moderate drought conditions; II: severe drought conditions; III: Extreme drought conditions.

**Table S5**. Overall drought-related mortality association (relative risks (RR), and 95% confidence intervals) by types of droughts based on its accumulation period (short-, medium-, long-, very long- term droughts) in South Africa between 2009 and 2016. Results are indicated for the total population and for each group of sex and age, and cause of mortality. SPEI,1,6,12,15= Standardized Precipitation Evapotranspiration Index measured at 1,6,12, and 15 months of accumulation, respectively. A00-B00= mortality due to certain infectious and parasitic diseases; E00-E90= mortality due to endocrine, metabolic, and nutritional causes; I00-I99= mortality due to circulatory conditions; J00-J99= mortality due to respiratory conditions.

**Table S6.** Coefficients, relative risks (RRs), 95% confidence intervals, and p-values obtained in the metanalytical model for A) different levels of urbanicity (“Other” and “Urban) using as reference the “Rural” level, and B) a multidimensional poverty index (MPI) in South Africa. * p<0.05; # tendency to be significant (p<0.07).

**Table S7**. Relative risks of mortality and 95% confidence intervals associated with short-term droughts (SPEI measured at one month of accumulation, SPEI1) by district municipality in South Africa between 2009 and 2016.

**Table S8**. Relative risks of mortality and 95% confidence intervals associated with medium-term droughts (SPEI measured at six months of accumulation, SPEI6) by district municipality in South Africa between 2009 and 2016.

**Table S9**. Relative risks of mortality and 95% confidence intervals associated with long-term droughts (SPEI measured at twelve months of accumulation, SPEI12) by district municipality in South Africa between 2009 and 2016.

**Table S10**. Relative risks of mortality and 95% confidence intervals associated with very long-term droughts (SPEI measured at fifteen months of accumulation, SPEI15) by district municipality in South Africa between 2009 and 2016.


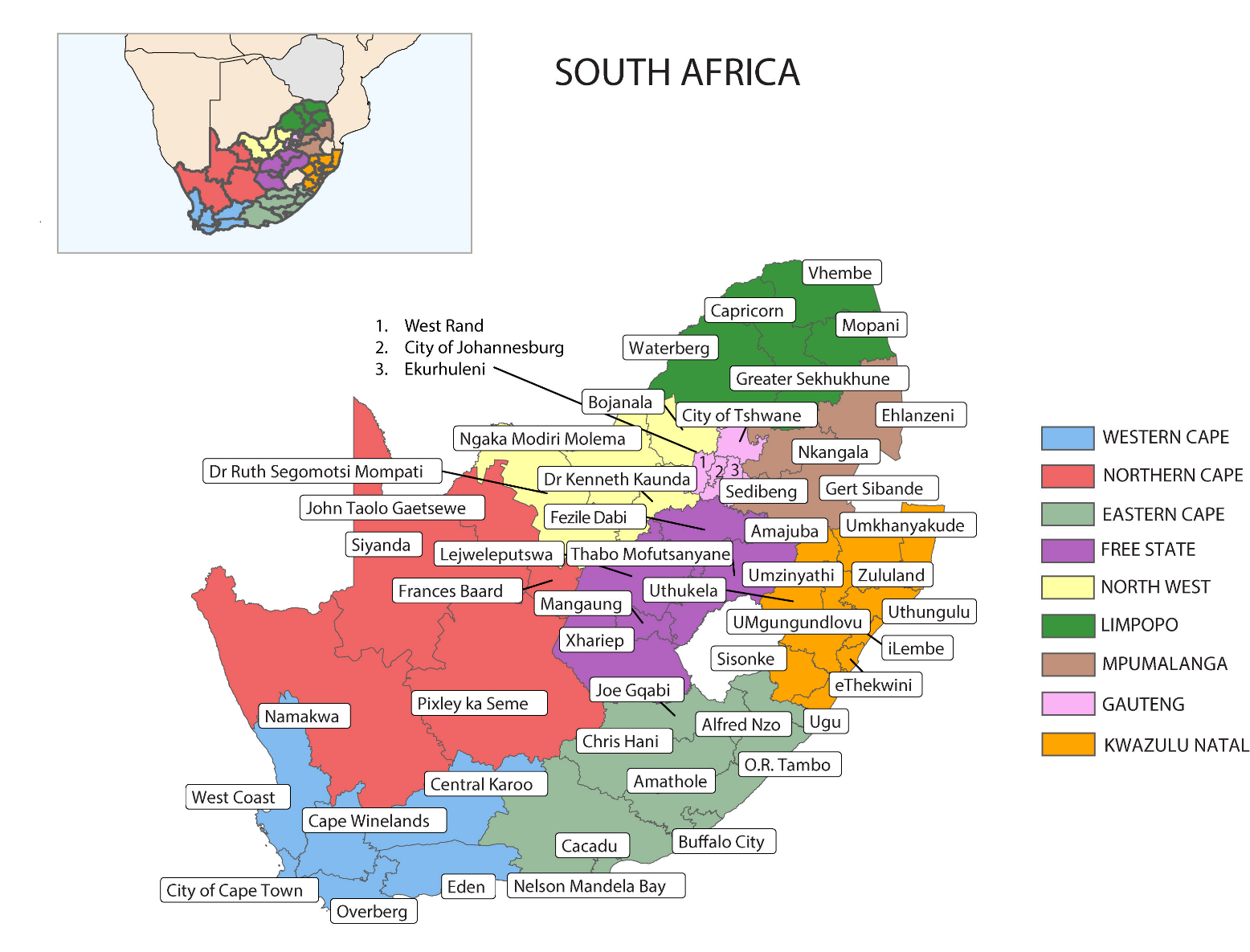


**Figure S1.** Study region showing the 52 district municipalities of South Africa, grouped into 9 provinces (colours) within the broader Southern African region. South Africa is a country located at the southern tip of Africa. It has a population of 60.14 million people and covers a geographical extent of 1219602 km^2^ [1,2]. South Africa is known for its cultural and linguistic diversity [2], but also its heterogeneous climate due to the irregular topography and its contrasting oceanic conditions of the warm Agulhas Current on the east coast and the upwelling cold Benguela Current on the west coast. Associated with these oceanic conditions and the mean positions of the subtropical anticyclones, there is a strong east-to-west decreasing gradient in precipitation and increasing aridity [3]. The region is often influenced by high-pressure systems, which result in sinking air and inhibit cloud formation and rainfall [4], leading to an average annual rainfall of 464 mm, which is substantially below the global average [1]. Additionally, the semi-arid climate of South Africa leads to high evaporation rates, resulting in water loss and increasing vulnerability to drought and water scarcity conditions [5].

[1] South African Government, 2023a. Geography and Climate. <https://www.gov.za/about-sa/geography-and-climate> (accessed: 9.8.23).

[2] South African Government, 2023b. South Africa’s people. <https://www.gov.za/about-sa/south-africas-people> (accessed: 9.8.23).

[3] Reason CJC. 2017: The Climate of Southern Africa, In Oxford Encyclopedia of Climate Science. Oxford University Press. <https://doi.org/10.1093/acrefore/9780190228620.013.513>

[4] Akoon I, Archer E, Colvin C, Davis C, Diedericks GPJ, Engelbrecht F, Fatti C, Le Maitre D, Landman W, le Roux A, Lötter D, Maserumule R, Meyer AA, Midgley G, Nkambule C, Olwoch J, Rossouw M, Sinden L-A, Stevens N, Theron AK, van Huyssteen E, Vogel C, Warbuton M, Wright C, Matherry A. n.d. South African Risk and Vulnerability Atlas, Ministry of Science and Technology, Pretoria, South Africa. <https://www.dffe.gov.za/sites/default/files/docs/sarva_atlas.pdf>

[5] Orimoloye IR, Belle JA, Orimoloye YM, Olusola AO, Ololade OO. 2022. Drought: A Common Environmental Disaster. Atmosphere 13, 111. <https://doi.org/10.3390/atmos13010111>

**Method S1.** Quasi-Poisson regression model to estimate the effects of drought on weekly mortality in each district municipality of South Africa between 2009 and 2016.

$Y_{ti}$ ~quasi-Poisson ($\mu_{it}$)

Log ($\mu_{it}$) = α + $\beta_{1}$ ${SPEIn}_{it}$ + cb (${Tmean}_{it}, lag=3$) + $M_{t}$ + $S_{t}$

$Y_{ti}$ corresponds to the number of mortality outcomes (independently for each cause of death and population group) on week *t* in location *i* in South Africa; $\alpha$ is the intercept; ${SPEIn}_{it}$refers to the drought term (we used the continuous series of the drought index obtained at a specific accumulation period, and applied a threshold parametrization (threshold=-0.84) to model the exposure-response association during the same week of exposure, assuming a linear association for values below that threshold); *cb* is the crossbasis function of mean temperature (*Tmean*) during 0-3 lag weeks, including three internal knots at the 10^th^, 75^th^, 90^th^ percentiles of each mean temperature series. $M_{t}$ and $S_{t}$ represent the adjustment of the seasonal and long-trends applying spline functions for time variables.

**Table S1**. Quasi-Akaike information of models with different specifications to control the seasonality and long-term trend. ns= natural spline function; df= degrees of freedom; date: date in the time series; week: indicator of the week within the year.

| Drought  Indicator | Population | ns(data$date, df=2*8)+ns(data$week, df=2) | ns(data$date, df=3*8)+ns(data$week, df=2) | ns(data$date, df=4*8)+ns(data$week, df=2) | ns(data$date, df=2*8)+ns(data$week, df=3) | ns(data$date, df=3*8)+ns(data$week, df=3) | ns(data$date, df=4*8)+ns(data$week, df=3) | ns(data$date, df=2*8)+ns(data$week, df=4) | ns(data$date, df=3*8)+ns(data$week, df=4) | ns(data$date, df=4*8)+ns(data$week, df=4) | ns(data$date, df=5*8)+ns(data$week, df=4) |
| --- | --- | --- | --- | --- | --- | --- | --- | --- | --- | --- | --- |
| SPEI1 | Total  Children  Pre-adults  Y-adults  O-adults  Elderly  Males  Females  A00-B99  E00-E90  I00-I99  J00-J99 | 213236.3  110834.5  107389.6  147987.3  146624.7  149688.1  175836.3  167198.6  131260.4  101617.7  136962.7  130858.5 | 213882.3  110679.1  107711.9  148176.2  146893.9  150266.6  176332  167692.6  131689.6  102055.9  137173.2  130801.2 | 215109.3  110819.2  108111.8  148839.7  147532.4  150807  177208.9  168463  132171.6  102497.6  137637.1  131073.2 | 213412.2  110609.2  107439.7  148002.3  146697.4  149764.7  175951.9  167292.4  131285.8  101665.3  137012.5  130807.9 | 214041.4  110692.7  107762.7  148262.6  146989.4  150380.4  176429.5  167806.3  131729.7  102076.4  137259  130831 | 215247  110818.5  108205.9  148968.4  147600.7  150872.1  177293.8  168574.6  132234.4  102533.1  137709.8  131141.2 | 213451.9  110647.5  107504.5  148077.6  146750.7  149799.9  176021  167337.2  131342.2  101713.9  137042.9  130795.7 | 214320  110687.1  107871  148420.7  147088.6  150404.9  176640.9  167927.2  131830.3  102172.5  137307.5  130915 | 215662.5  110900.4  108258.9  149080.8  147759.7  151037.2  177582.7  168742.1  132304.6  102611.1  137803.4  131248.1 | 216937.3  111088.5  108803.3  149871.2  148448.3  151586  178569.2  169544.9  132754.4  103035.6  138319.6  131620.7 |
| SPEI6 | Total  Children  Pre-adults  Y-adults  O-adults  Elderly  Males  Females  A00-B99  E00-E90  I00-I99  J00-J99 | 213334.8  110868.5  107397.7  148096.7  146718.3  149746.2  175960.5  167189.5  131298.7  101618.7  137003.6  130937.4 | 214066.8  110722.2  107730.4  148268.7  147027.6  150346.3  176507.9  167717.4  131724.8  102079.2  137204.3  130890.7 | 215267.2  110832.8  108145.6  148941.6  147635.7  150878.1  177350.9  168491.3  132207.3  102507.9  137662  131130.9 | 213480.2  110641.4  107450.8  148072.6  146769.3  149817.3  176057  167264.5  131323.8  101672.5  137046.7  130839.9 | 214197.8  110727.7  107783.8  148348.9  147112.1  150455.4  176585.3  167818.6  131760  102095.8  137292.4  130912.9 | 215449.8  110842.5  108237  149077  147715.3  150944.2  177459.4  168626.8  132272.2  102551.5  137744.8  131209.9 | 213473.8  110678.5  107515.1  148122.8  146802.2  149842.3  176096.2  167294.4  131374.4  101724  137051.2  130803.8 | 214470.2  110717.7  107886.6  148504.3  147202.8  150483.6  176796.3  167937.8  131869.9  102204.2  137346.2  130963.2 | 215828.9  110906.5  108294.6  149195.9  147863.5  151107.7  177726.4  168776.7  132343.4  102623.2  137832.4  131310.9 | 217103.6  110906.5  108814.5  149986.4  148556.7  151643.4  178707.4  169569.8  132797.5  103046.9  138355.8  131657.4 |

| SPEI12 | Total  Children  Pre-adults  Y-adults  O-adults  Elderly  Males  Females  A00-B99  E00-E90  I00-I99  J00-J99 | 213277.2  110877.2  107394.8  148083.7  146721  149740.3  175908.4  167205.6  131278.9  101621  136985.9  130936.8 | 213982.5  110691.3  107711.8  148243.1  147012.7  150337.2  176419.6  167721  131732.6  102082  137204.9  130849.2 | 215233.9  110808.3  108146.8  148923.8  147640.6  150890  177304.3  168494.4  132223.7  102511.6  137690  131109.2 | 213414.2  110640.5  107440.8  148056.2  146769.7  149806.5  175993.7  167282.1  131313.8  101672.3  137020  130831 | 214133.8  110694.6  107761.6  148326.5  147098.9  150452.5  176502.2  167837.9  131777.8  102098.3  137287.3  130878 | 215396.5  110811.7  108239.5  149054.8  147719.1  150949.9  177398.5  168624.2  132289.3  102550.5  137766.3  131190.8 | 213399.4  110670.1  107503.1  148108.7  146797  149836.8  176017.5  167315.8  131368.7  101718.2  137033.1  130784.7 | 214371.5  110682.9  107869  148476.4  147178.7  150478.2  176692.9  167939.6  131870.3  102201.7  137336.3  130914.3 | 215792.1  110874.5  108288.6  149173.6  147866.5  151128.8  177682.4  168781  132355.8  102623.7  137852.2  131289.7 | 217079.6  111097.4  108852.1  149956.6  148584.4  151635  178679.4  169576.9  132814.2  103053.2  138367.8  131690.3 |
| --- | --- | --- | --- | --- | --- | --- | --- | --- | --- | --- | --- |
| SPEI15 | Total  Children  Pre-adults  Y-adults  O-adults  Elderly  Males  Females  A00-B99  E00-E90  I00-I99  J00-J99 | 213176.9  110913.5  107353.1  148050.2  146703.9  149696.6  175870.3  167128.5  131338.8  101628  136956.7  130878.6 | 213949.2  110714  107698.5  148237.5  146995.6  150302.9  176438.8  167663.9  131754.7  102078.7  137168.4  130840.8 | 215136  110822.5  108109.4  148910.3  147608.4  150830.3  177258  168434.1  132225  102508.7  137645.1  131117.1 | 213320.3  110686.6  107404.5  148029.8  146750.3  149762.4  175962  167202.8  131368.5  101681  136993.8  130768.7 | 214111.1  110724.9  107757.1  148330.1  147090  150416.7  176532.6  167780.3  131793.1  102093.1  137265.1  130870.9 | 215332.5  110822  108203.2  149053  147698.5  150902.8  177380.3  168573.4  132299.1  102548.1  137732.3  13119I.2 | 213346.2  110732.4  107470.7  148083.8  146783.6  149809.4  176016.4  167250  131428.9  101731.2  137011.8  130733.2 | 214355.1  110713.4  107854.6  148477.1  147167.3  150438.9  176729.5  167887  131903.6  102198.6  137304.9  130916.6 | 215717.1  110900.8  108259.2  149168.7  147846.6  151061.6  177652.3  168720.7  132369.8  102619  137809.5  131297.7 | 217022.7  111102.8  108805.7  149950.7  148562.4  151612.1  178661.6  169538.3  132838.3  103060.4  138356  131683.8 |

| Drought  Indicator | Population | ns(data$date, df=3*8) | ns(data$date, df=4*8) | ns(data$date, df=5*8) | ns(data$date, df=3*8)+Seas | ns(data$date, df=4*8)+Seas | ns(data$date, df=5*8)+Seas |
| --- | --- | --- | --- | --- | --- | --- | --- |
| SPEI1 | Total  Children  Pre-adults  Y-adults  O-adults  Elderly  Males  Females  A00-B99  E00-E90  I00-I99  J00-J99 | 215620.2  110576.4  108301.2  149031.5  147327.4  150450.1  177849.6  168024.9  131664.3  102042.7  137367.7  131078.6 | 216343.5  110708.6  108655.7  149637.3  147821.9  150881.6  178337.4  168668.2  132097.1  102431.9  137742.6  131252.1 | 217558.4  110887.6  109151.3  150357.4  148493.2  151391.7  179276  169453.9  132569.5  102856.2  138215.6  131593.3 | 214968.6  110605.4  108253.6  148834.8  147254.8  150488.2  177500.3  167875.6  131610.8  102130.1  137436.1  131202.1 | 215050.9  110694.1  108553.7  149259.5  147545.6  150745.6  177639.6  168242.7  131907.4  102503.2  137659.2  131322 | 216318.8  110916.4  109075.6  150020.8  148269.5  151234.3  178625.5  169031.8  132380.4  102915.7  138122.1  131669.8 |
| SPEI6 | Total  Children  Pre-adults  Y-adults  O-adults  Elderly  Males  Females  A00-B99  E00-E90  I00-I99  J00-J99 | 215998.5  110629.4  108348.2  149189.7  147527.8  150503.1  178175.7  168108.4  131712.6  102071.2  137446.8  131216.2 | 216516.7  110730.2  108680.6  149714  147931  150899.3  178476.4  168716.7  132144.2  102453.4  137787.8  131319 | 217710  110932.8  109158.7  150432.9  148602.7  151395  179401.9  169481  132619.3  102876.5  138259  131646.6 | 215272.4  110668.3  108286  148973.6  147435  150518.6  177776.6  167932.3  131658.4  102155.8  137495.6  131320.6 | 215183.8  110724.7  108569.9  149319.3  147645.9  150764.8  177752.3  168273.7  131954.6  102526.9  137686.8  131379.7 | 216435.3  110962.7  109081.6  150081.1  148360.7  151247.7  178731.1  169040.9  132427  102942.1  138157.8  131721.3 |
| SPEI12 | Total  Children  Pre-adults  Y-adults  O-adults  Elderly  Males  Females  A00-B99  E00-E90  I00-I99  J00-J99 | 215877.1  110592.1  108351.4  149171.9  147503.9  150464.3  178085.8  168075.3  131721.7  102065  137439.3  131149.6 | 216484.2  110694.1  108708.3  149723.6  147937  150889.7  178456  168696.9  132156  102447.3  137797.8  131276.9 | 217689.4  110900.5  109207.8  150423.1  148616.4  151368.5  179386.1  169474.9  132624.9  102878.6  138253.6  131647.5 | 215164.8  110623.3  108290.6  148957.3  147408  150418.1  177699.2  167897.2  131667.6  102147.3  137495.8  131246.5 | 215088.1  110682  108605  149299  147642.3  150734.2  177699.3  168217.6  131968.3  102501.2  137688.5  131318.4 | 216356.2  110925.2  109132.4  150054.4  148375.2  151198.7  178703.7  168982.6  132436.9  102920  138138.1  131696.6 |
| SPEI15 | Total  Children  Pre-adults  Y-adults  O-adults  Elderly  Males  Females  A00-B99  E00-E90  I00-I99  J00-J99 | 215930.7  110616.3  108346  149183.3  147503.7  150461.1  178157  168050.9  131746.3  102062.1  137420.1  131163.8 | 216429.2  110711.9  108674.3  149716.6  147922  150845.5  178442.3  168643.3  132158.6  102446.8  137761.6  131297.7 | 217633.4  110909.8  109173.2  150414.8  148600.1  151366.9  179364.7  169430.6  132643.1  102884.1  138247  131655.4 | 215211.5  110652  108277.5  148961.8  147408  150478.7  177749.9  167888  131688.1  102141  137471.9  131262.4 | 215115.2  110705.1  108557.4  149327.5  147642.3  150708.9  177717.4  168220.3  131973.2  102509.5  137668.5  131360.7 | 216403.7  110948.4  109096.6  150076.4  148375.2  151223.2  178711.6  169016.7  132462.5  102946.2  138157.3  131726.5 |

**Table S2.** Classification of the 52 district municipalities of South Africa according to the district-level urbanicity and socioeconomic deprivation. The district municipalities classified as “Urban” correspond to the eight metropolitan municipalities in the country (https://www.gov.za/about-government/government-system/local-government), while the those classified as “Rural” corresponded to the rural nodes indicated in the 2011 Census (http://www.statssa.gov.za/publications/Report-03-10-09/Report-03-10-092011.pdf). The rest of district municipalities were classified as “Other”. A district-level multidimensional poverty index (MPI) in South Africa was used as a continuous variable using information in 2011 Census. That index accounts for twelve indicators capturing deprivation for four dimensions [1].

| District municipalities | Urbanicity level | Multidimensional poverty Index (MPDI) | District municipalities | Urbanicity level | Multidimensional poverty Index (MPDI) | District municipalities | Urbanicity level | Multidimensional poverty Index (MPDI) |
| --- | --- | --- | --- | --- | --- | --- | --- | --- |
| Alfred Nzo | No-Urban | 0.0913 | eThekwini | Urban | 0.0171 | Sedibeng | Other | 0.0136 |
| Amajuba | Other | 0.0264 | Fezile Dabi | Other | 0.0190 | Sisonke | No-Urban | 0.0668 |
| Amathole | No-Urban | 0.0534 | Frances Baard | Other | 0.0264 | Siyanda | Other | 0.0201 |
| Bojanala | Other | 0.0277 | Gert Sibande | Other | 0.0283 | Thabo Mofutsanyane | No-Urban | 0.0268 |
| Buffalo City | Urban | 0.0534 | Greater Sekhukhune | No-Urban | 0.0447 | Ugu | No-Urban | 0.0570 |
| Cacadu | Other | 0.0160 | iLembe | Other | 0.0459 | UMgungundlovu | No-Urban | 0.0294 |
| Cape Winelands | Other | 0.0064 | Joe Gqabi | No-Urban | 0.0626 | Umkhanyakude | No-Urban | 0.0579 |
| Capricorn | Other | 0.292 | John Taolo Gaetsewe | No-Urban | 0.0483 | Umzinyathi | No-Urban | 0.0726 |
| Central Karoo | No-Urban | 0.0122 | Lejweleputswa | Other | 0.0215 | Uthukela | No-Urban | 0.0472 |
| Chris Hani | No-Urban | 0.0627 | Mangaung | Urban | 0.0173 | Uthungulu | Other | 0.0471 |
| City of Cape Town | Urban | 0.0096 | Mopani | No-Urban | 0.0390 | Vhembe | Other | 0.0384 |
| City of Johannesburg | Urban | 0.0096 | Namakwa | Other | 0.0113 | Waterberg | Other | 0.0271 |
| City of Tshwane | Urban | 0.0133 | Nelson Mandela Bay | Urban | 0.0153 | West Coast | Other | 0.0057 |
| Dr Kenneth Kaunda | Other | 0.0193 | Ngaka Modiri Molema | Other | 0.0529 | West Rand | Other | 0.0235 |
| Dr Ruth Segomotsi Mompati | Other | 0.0495 | Nkangala | Other | 0.0197 | Xhariep | Other | 0.0233 |
| Eden | Other | 0.0135 | O.R.Tambo | No-Urban | 0.0857 | Zululand | No-Urban | 0.0451 |
| Ehlanzeni | No-Urban | 0.0290 | Overberg | Other | 0.0090 |  |  |  |
| Ekurhuleni | Urban | 0.0169 | Pixley ka Seme | Other | 0.0247 |  |  |  |

[1] Fransman, T., Yu, D. 2018. Multidimensional poverty in South Africain 2001-2016. Stellenbosch Economic Working Papers: WP07/2018.www.ekon.sun.ac.za/wpapers/2018/wp072018. Access: 11.03.2024 on 10 September

**Table S3**. Descriptive statistics corresponding to weekly counts of all-cause mortality and specific causes of the total population (A00-B99= certain parasitic and infectious diseases; E00-E90= nutritional, endocrine and metabolic diseases; I00-I99= circulatory diseases; J00-J99= respiratory diseases) for each district municipality of South Africa and overall, between 2009 and 2016. Total number of all-cause deaths separated by sex (males, females) and age (<5, 5-24, 25-44, 45-64, ≥65 years) groups were also reported. During the study period, a total of 3,757,736 deaths were assessed across all district municipalities of South Africa, with 52% males and 47.7% females. According to age groups, the lowest percentage of deaths was observed in pre-adults (5-24 years), constituting 6.4% of the total deaths in South Africa, in contrast to the oldest population (65 years and above), accounting for 31%. Furthermore, 17.5% of all analyzed deaths were due to certain parasitic and infectious diseases; 5.5% to endocrine, nutritional, and metabolic causes; 20.9% to circulatory conditions, and 16.1% to respiratory diseases. The highest values of deaths were recorded in the City of Johannesburg (n=231888 deaths), eThekwini (n=208860) and the city of Cape Town (n=206999), while the lowest number of deaths were observed in Overberg (n=18059), Namakwa (n=9355), and Central Karoo (n=6271).

| District municipalities | all | 0-4 years | 5-24 years | 25-44 years | 45-64 years | >= 65 years | males | females | A00B99 | E00E90 | I00I99 | J00J99 |
| --- | --- | --- | --- | --- | --- | --- | --- | --- | --- | --- | --- | --- |
| Alfred Nzo | 47818 | 3166 | 4024 | 14113 | 11607 | 14828 | 23771 | 23922 | 6318 | 1392 | 3817 | 4950 |
| Amajuba | 40167 | 3072 | 2829 | 12688 | 11124 | 10350 | 20238 | 19831 | 7608 | 2958 | 9325 | 8515 |
| Amathole | 113409 | 4654 | 8369 | 27713 | 29509 | 42988 | 58262 | 54905 | 20748 | 5506 | 21179 | 19790 |
| Bojanala | 99790 | 8871 | 5406 | 26956 | 28021 | 30310 | 53305 | 46131 | 16057 | 6551 | 21029 | 16886 |
| Buffalo City | 68130 | 2915 | 4243 | 17515 | 19992 | 23384 | 34927 | 33059 | 13004 | 3094 | 16206 | 11099 |
| Cacadu | 42852 | 2378 | 2132 | 9936 | 13657 | 14677 | 22354 | 20392 | 7459 | 1803 | 10988 | 6071 |
| Cape Winelands | 50250 | 2621 | 2347 | 9757 | 16546 | 18890 | 27460 | 22675 | 7048 | 1631 | 13164 | 7326 |
| Capricorn | 98802 | 8741 | 5432 | 24559 | 24900 | 35111 | 49117 | 49527 | 16949 | 7671 | 17436 | 19571 |
| Central Karoo | 6271 | 358 | 270 | 1225 | 2147 | 2265 | 3262 | 3000 | 896 | 177 | 1847 | 1022 |
| Chris Hani | 73819 | 3828 | 5037 | 18761 | 20091 | 26016 | 38127 | 35559 | 12788 | 3836 | 15115 | 13067 |
| City of Cape Town | 206999 | 10972 | 12521 | 42782 | 59388 | 80851 | 113329 | 93048 | 26854 | 7065 | 55247 | 26714 |
| City of Johannesburg | 231888 | 19196 | 12189 | 66099 | 63908 | 69589 | 122750 | 107598 | 33397 | 8514 | 43759 | 32018 |
| City of Tshwane | 156086 | 11107 | 7499 | 38018 | 44034 | 55237 | 81572 | 74025 | 22433 | 10571 | 43583 | 26547 |
| Dr Kenneth Kaunda | 58191 | 5313 | 3080 | 15541 | 17864 | 16318 | 31481 | 26553 | 12307 | 3012 | 11162 | 9505 |
| Dr Ruth Segomotsi Mompati | 47585 | 5935 | 2548 | 12463 | 12837 | 13773 | 24832 | 22645 | 9718 | 2875 | 8944 | 8985 |
| Eden | 39591 | 1660 | 1636 | 7110 | 12623 | 16520 | 21602 | 17945 | 5195 | 1340 | 11736 | 6153 |
| Ehlanzeni | 120044 | 8686 | 9384 | 39150 | 30610 | 31789 | 61220 | 58415 | 30693 | 7836 | 22336 | 18877 |
| Ekurhuleni | 203198 | 18900 | 11570 | 58844 | 57670 | 55677 | 108115 | 94121 | 27868 | 9572 | 40692 | 32476 |
| eThekwini | 208860 | 11714 | 15896 | 60363 | 56778 | 63599 | 108901 | 99612 | 36500 | 10603 | 54219 | 26095 |
| Fezile Dabi | 47304 | 3906 | 2313 | 12114 | 14325 | 14606 | 24825 | 22427 | 7973 | 3761 | 11764 | 9703 |
| Frances Baard | 25952 | 1968 | 1353 | 6699 | 8167 | 7735 | 13608 | 12262 | 4565 | 1429 | 5236 | 4324 |
| Gert Sibande | 87982 | 8986 | 6614 | 27476 | 24070 | 20655 | 45452 | 42331 | 18191 | 5944 | 15087 | 16244 |
| Greater Sekhukhune | 78465 | 5953 | 4870 | 20822 | 19659 | 27107 | 38346 | 39950 | 15087 | 5043 | 17382 | 19615 |
| iLembe | 43433 | 3228 | 4018 | 13290 | 10886 | 11874 | 22230 | 21080 | 11212 | 2778 | 8705 | 5430 |
| Joe Gqabi | 38654 | 2366 | 2516 | 10092 | 10514 | 13117 | 19463 | 19106 | 6485 | 1631 | 6279 | 5270 |
| John Taolo Gaetsewe | 23835 | 3196 | 1487 | 6481 | 6638 | 6006 | 12519 | 11281 | 4179 | 984 | 3243 | 4236 |
| Lejweleputswa | 71294 | 6959 | 4131 | 20364 | 22011 | 17729 | 37680 | 33440 | 11710 | 4611 | 13553 | 15547 |
| Mangaung | 65510 | 4601 | 3698 | 18411 | 19426 | 19286 | 34082 | 31211 | 10003 | 3508 | 10672 | 10918 |
| Mopani | 81225 | 9593 | 5022 | 21350 | 20162 | 24990 | 40004 | 41041 | 15457 | 6485 | 10715 | 13151 |
| Namakwa | 9355 | 409 | 357 | 1600 | 2955 | 4025 | 5136 | 4215 | 734 | 331 | 2639 | 1363 |
| Nelson Mandela Bay | 71893 | 3652 | 3780 | 17388 | 22878 | 24112 | 37494 | 34242 | 12792 | 3921 | 18941 | 9313 |
| Ngaka Modiri Molema | 75141 | 7979 | 4242 | 20552 | 20778 | 21486 | 39471 | 35433 | 12415 | 4014 | 15738 | 14234 |
| Nkangala | 84672 | 6122 | 5262 | 24585 | 25107 | 23430 | 44168 | 40261 | 13898 | 5644 | 19156 | 17176 |
| O.R.Tambo | 98749 | 5531 | 10515 | 30965 | 23136 | 28517 | 49350 | 49073 | 19134 | 3778 | 11387 | 10643 |
| Overberg | 18059 | 872 | 859 | 3231 | 5333 | 7727 | 10232 | 7803 | 2032 | 614 | 5092 | 2508 |
| Pixley ka Seme | 28010 | 2100 | 1498 | 6901 | 9130 | 8345 | 14464 | 13463 | 4694 | 1433 | 6539 | 5206 |
| Sedibeng | 84263 | 6616 | 4651 | 22218 | 26103 | 24522 | 45144 | 38882 | 14084 | 5011 | 20157 | 16576 |
| Sisonke | 45969 | 3387 | 4161 | 14416 | 11719 | 12192 | 23051 | 22798 | 9581 | 2882 | 8060 | 6935 |
| Siyanda | 22974 | 1848 | 1224 | 5987 | 7254 | 6635 | 12309 | 10624 | 3886 | 1083 | 4611 | 4573 |
| Thabo Mofutsanyane | 84847 | 8172 | 4939 | 24896 | 24172 | 22546 | 42956 | 41751 | 15299 | 6337 | 18167 | 19101 |
| Ugu | 78163 | 5121 | 6657 | 22888 | 18814 | 24576 | 39957 | 38086 | 17041 | 4883 | 16246 | 12280 |
| UMgungundlovu | 77883 | 3584 | 5421 | 23499 | 21066 | 24213 | 39769 | 37928 | 14242 | 5252 | 19067 | 11338 |
| Umkhanyakude | 38560 | 3575 | 3456 | 12360 | 8391 | 10655 | 19287 | 19026 | 10079 | 2123 | 5812 | 3773 |
| Umzinyathi | 44574 | 4136 | 3683 | 12611 | 10880 | 13127 | 22002 | 22441 | 9103 | 2769 | 8887 | 6692 |
| Uthukela | 59353 | 4933 | 4654 | 18520 | 15338 | 15828 | 30199 | 29046 | 14065 | 3764 | 13410 | 9265 |
| Uthungulu | 59743 | 4650 | 5211 | 18834 | 14743 | 16063 | 30580 | 28998 | 13138 | 3404 | 10604 | 7594 |
| Vhembe | 67458 | 6732 | 3988 | 15242 | 16956 | 24416 | 33214 | 34121 | 10840 | 5747 | 9305 | 7741 |
| Waterberg | 43377 | 4316 | 2458 | 11391 | 10994 | 14178 | 22475 | 20837 | 6967 | 3078 | 8572 | 7647 |
| West Coast | 28388 | 1360 | 1332 | 5497 | 9340 | 10818 | 15868 | 12485 | 4054 | 959 | 7965 | 4079 |
| West Rand | 73212 | 6230 | 3984 | 20559 | 21688 | 20502 | 39858 | 32921 | 10930 | 3154 | 14991 | 12123 |
| Xhariep | 22514 | 1618 | 1142 | 6045 | 6821 | 6866 | 11783 | 10686 | 3395 | 1200 | 4278 | 4672 |
| Zululand | 63175 | 6405 | 5738 | 20544 | 14649 | 15668 | 31531 | 31451 | 16065 | 3522 | 9593 | 9057 |
| TOTAL | 3757736 | 284191 | 241646 | 1 021421 | 1037409 | 1165724 | 1953132 | 1793663 | 657170 | 207084 | 783637 | 603994 |

**Table S4**. Descriptive statistics corresponding to drought conditions measured by weekly SPEI calculated at short-, medium-, long-, and very long- term (SPEI1,6,12,15, respectively) and weekly mean temperature for each district municipality and province of South Africa and overall, between 2009 and 2016. D= number of weeks with drought; I: moderate drought conditions; II: severe drought conditions; III: Extreme drought conditions. The average total number of weeks with drought conditions ranged from 103 using the SPEI1 to 126 using the SPEI12 across the 8 years in South Africa. This trend was highly consistent across most provinces, with some variations at the district municipality level. Regardless of the time scale of the SPEI used, moderate drought was the most common event, followed by severe drought, and extreme drought. Drought conditions seemed to be more frequent in provinces of Northwest (e.g., Dr. Kenneth Kaunda, Ngaka Modiri Molema), Free State (e.g., Thabo Mofutsanyane, Fezile Dabi), and Gauteng (e.g., Sedibeng, West Rand, City of Johannesburg), while in provinces of Limpopo (e.g., Capricorn, Vhembe, Greater Sekhukhune) and Western Cape (e.g., West Coast, Overberg) the number of events was lower. Extreme conditions of short- and medium-term droughts were particularly frequent in the district municipalities of Frances Baard (Northern Cape), Amajuba, uMzinyathi, and uThukela (Kwazulu-Natal). The frequency of extreme conditions of long- and very long-term droughts was particularly high in Cacadu and Nelson Mandela Bay (Eastern Cape) as well as in most of those located in the provinces of Free State, Northwest, and Gauteng. The average mean temperature in South Africa during the studied period was 17.8°C, with variations from 13.8°C in Joe Gqabi (Eastern Cape) and 22°C in Umkhanyakude (Kwazulu-Natal).

| Province | District Municipality | SPEI1 (weeks) | | | | SPEI6 (weeks) | | | | SPEI12 (weeks) | | | | SPEI5 (weeks) | | | | Tmean (°C);  (min, max) |
| --- | --- | --- | --- | --- | --- | --- | --- | --- | --- | --- | --- | --- | --- | --- | --- | --- | --- | --- |
|  |  | D | I | II | III | D | I | II | III | D | I | II | III | D | I | II | III |  |
| Western  Cape | West Coast | 78 | 35 | 19 | 24 | 80 | 49 | 19 | 12 | 86 | 43 | 30 | 13 | 74 | 29 | 29 | 16 | 17.6 (9.1, 28.4) |
|  | Cape Winelands | 87 | 44 | 25 | 18 | 96 | 58 | 24 | 14 | 101 | 53 | 47 | 1 | 101 | 61 | 24 | 16 | 16.1 (6.9, 26.3) |
|  | Overberg | 90 | 39 | 30 | 21 | 96 | 67 | 27 | 2 | 77 | 49 | 25 | 3 | 63 | 39 | 21 | 3 | 16.3 (9, 24.9) |
|  | Eden | 90 | 38 | 27 | 25 | 120 | 53 | 48 | 19 | 115 | 40 | 36 | 39 | 106 | 29 | 30 | 47 | 16.1 (8, 25) |
|  | Central Karoo | 93 | 50 | 24 | 19 | 118 | 70 | 31 | 17 | 142 | 65 | 58 | 19 | 128 | 61 | 37 | 30 | 16.6 (5.6, 26) |
|  | City of Cape Town | 88 | 44 | 26 | 18 | 120 | 81 | 27 | 12 | 125 | 77 | 43 | 5 | 132 | 71 | 57 | 4 | 16.6 (9.7, 26.6) |
|  | **Total** (average) | **87.7** | **41.7** | **25.2** | **20.8** | **105** | **63** | **29.3** | **12.7** | **107.7** | **54.5** | **39.8** | **13.3** | **100.7** | **48.3** | **33** | **19.3** | **16.5 (8.1, 26.2)** |
| Northern  Cape | Namakwa | 81 | 44 | 21 | 16 | 65 | 48 | 11 | 6 | 89 | 71 | 18 | 0 | 91 | 75 | 14 | 2 | 17.7 (7.2, 28.1) |
|  | Pixley ka Seme | 86 | 40 | 21 | 25 | 101 | 46 | 29 | 26 | 109 | 50 | 47 | 12 | 110 | 40 | 52 | 18 | 17.9 (5.1, 29.6) |
|  | Siyanda | 90 | 43 | 21 | 26 | 108 | 50 | 35 | 23 | 124 | 85 | 34 | 5 | 119 | 65 | 42 | 12 | 20.6 (7.2, 32.1) |
|  | Frances Baard | 113 | 52 | 33 | 28 | 156 | 77 | 46 | 33 | 168 | 70 | 59 | 39 | 160 | 62 | 50 | 48 | 19 (6.1, 31.2) |
|  | John Taolo Gaetsewe | 103 | 51 | 29 | 23 | 117 | 53 | 33 | 31 | 123 | 26 | 70 | 27 | 125 | 36 | 50 | 39 | 19.9 (7.1, 32.1) |
|  | **Total** (average) | **94.6** | **46** | **25** | **23.6** | **109.4** | **54.8** | **30.8** | **23.8** | **122.6** | **60.4** | **45.6** | **16.6** | **121** | **55.6** | **41.6** | **23.8** | **19 (6.5, 30.6 )** |
| Eastern  Cape | Cacadu | 93 | 48 | 22 | 23 | 134 | 70 | 38 | 26 | 141 | 27 | 69 | 45 | 113 | 21 | 30 | 62 | 16.8 (7.6, 25.89) |
|  | Amathole | 91 | 54 | 17 | 20 | 103 | 45 | 46 | 12 | 138 | 87 | 46 | 5 | 115 | 66 | 42 | 7 | 16.7 (8.8, 24.2) |
|  | Chris Hani | 99 | 51 | 26 | 22 | 110 | 60 | 28 | 22 | 119 | 58 | 37 | 24 | 139 | 91 | 35 | 13 | 15 (4.5, 23.5) |
|  | Joe Gqabi | 101 | 47 | 30 | 24 | 109 | 53 | 33 | 23 | 118 | 60 | 20 | 38 | 113 | 63 | 3 | 47 | 13.8 (2.1, 23.2) |
|  | O.R.Tambo | 104 | 56 | 29 | 19 | 106 | 55 | 36 | 15 | 124 | 71 | 51 | 2 | 129 | 81 | 42 | 6 | 16.8 (9.4, 23.8) |
|  | Alfred Nzo | 104 | 48 | 37 | 19 | 111 | 43 | 42 | 26 | 147 | 87 | 36 | 24 | 145 | 73 | 67 | 5 | 14.9 (4.4, 22.3) |
|  | Nelson Mandela Bay | 85 | 40 | 23 | 22 | 108 | 57 | 19 | 32 | 135 | 43 | 30 | 62 | 135 | 44 | 33 | 58 | 18 (11.5, 25) |
|  | Buffalo City | 90 | 55 | 19 | 16 | 105 | 52 | 41 | 12 | 129 | 62 | 55 | 12 | 102 | 31 | 59 | 12 | 18.1 (11.3, 24.9) |
|  | **Total** (average) | **95.9** | **49.9** | **25.4** | **20.6** | **110.8** | **54.4** | **35.4** | **21** | **131.4** | **61.9** | **43** | **26.5** | **123.9** | **58.8** | **38.9** | **26.25** | **16.3 (7.5, 24.1)** |
| Free  State | Xhariep | 108 | 57 | 29 | 22 | 125 | 69 | 32 | 24 | 128 | 64 | 25 | 39 | 137 | 69 | 17 | 51 | 16.9 (4.2, 28.9) |
|  | Lejweleputswa | 114 | 59 | 38 | 17 | 147 | 88 | 39 | 20 | 179 | 97 | 45 | 37 | 165 | 75 | 38 | 52 | 18 (5.6, 30) |
|  | Thabo Mofutsanyane | 130 | 73 | 33 | 24 | 155 | 72 | 54 | 29 | 179 | 105 | 28 | 46 | 208 | 146 | 9 | 53 | 14.9 (3.8, 24.7) |
|  | Fezile Dabi | 136 | 80 | 34 | 22 | 173 | 81 | 61 | 31 | 171 | 68 | 54 | 49 | 196 | 109 | 31 | 56 | 17 (5.6, 27.5) |
|  | Mangaung | 108 | 55 | 34 | 19 | 131 | 78 | 27 | 26 | 173 | 107 | 29 | 37 | 172 | 98 | 21 | 53 | 17.1 (4.3, 29.4) |
|  | **Total** (average) | **119.2** | **64.8** | **33.6** | **20.8** | **146.2** | **77.6** | **42.6** | **26** | **166** | **88.2** | **36.2** | **41.6** | **175.6** | **99.4** | **23.2** | **53** | **16.8 (4.7, 28.1)** |
| North West | Bojanala | 125 | 77 | 30 | 18 | 139 | 78 | 34 | 27 | 149 | 77 | 31 | 41 | 144 | 74 | 18 | 52 | 19.6 (8, 29.7) |
|  | Ngaka Modiri Molema | 125 | 74 | 36 | 15 | 189 | 131 | 38 | 20 | 179 | 51 | 89 | 39 | 172 | 48 | 75 | 49 | 18.7 (6.7, 29.7) |
|  | Dr Ruth Segomotsi Mompati | 106 | 53 | 33 | 20 | 149 | 89 | 39 | 21 | 156 | 72 | 49 | 35 | 140 | 48 | 43 | 49 | 19.5 (7.2, 31.4) |
|  | Dr Kenneth Kaunda | 131 | 76 | 38 | 17 | 178 | 100 | 57 | 21 | 180 | 70 | 67 | 43 | 182 | 86 | 38 | 58 | 17.9 (6.1, 29) |
|  | **Total** (average) | **121.8** | **70** | **34.3** | **17.5** | **163.8** | **99.5** | **42** | **22.3** | **166** | **67.5** | **59** | **39.5** | **159.5** | **64** | **43.5** | **52** | **18.9 (7, 29.9)** |
| Limpopo | Mopani | 108 | 56 | 34 | 18 | 90 | 40 | 25 | 25 | 78 | 27 | 11 | 40 | 82 | 32 | 13 | 37 | 21.7 (13.5, 29.2) |
|  | Vhembe | 89 | 48 | 26 | 15 | 72 | 40 | 18 | 14 | 51 | 6 | 34 | 11 | 52 | 14 | 35 | 3 | 21.7 (13, 30) |
|  | Capricorn | 78 | 44 | 20 | 14 | 74 | 42 | 17 | 15 | 51 | 7 | 41 | 3 | 44 | 13 | 31 | 0 | 19.9 (10.1, 28.4) |
|  | Waterberg | 94 | 53 | 23 | 18 | 92 | 49 | 21 | 22 | 79 | 30 | 21 | 28 | 84 | 35 | 7 | 42 | 20.4 (9.2, 29.7) |
|  | Greater Sekhukhune | 84 | 45 | 26 | 13 | 67 | 31 | 17 | 19 | 53 | 9 | 30 | 14 | 61 | 15 | 42 | 4 | 18.9 (8.6, 26.3) |
|  | **Total** (average) | **90.6** | **49.2** | **25.8** | **15.6** | **79** | **40.4** | **19.6** | **19** | **62.4** | **15.8** | **27.4** | **19.2** | **64.6** | **21.8** | **25.6** | **17.2** | **20.5 (10.9, 28.7)** |
| Mpumalanga | Gert Sibande | 124 | 67 | 32 | 25 | 139 | 72 | 35 | 32 | 124 | 58 | 21 | 45 | 116 | 47 | 19 | 50 | 15.4 (5.4, 22.8) |
|  | Nkangala | 107 | 61 | 24 | 22 | 119 | 79 | 19 | 21 | 116 | 65 | 24 | 27 | 93 | 34 | 17 | 42 | 16.4 (5.9, 24.5) |
|  | Ehlanzeni | 115 | 58 | 38 | 19 | 109 | 47 | 32 | 30 | 120 | 49 | 27 | 44 | 113 | 40 | 25 | 48 | 20.1 (12, 26.5) |
|  | **Total** (average) | **115.3** | **62** | **31.3** | **22** | **122.3** | **66** | **28.7** | **27.7** | **120** | **57.3** | **24** | **38.7** | **107.3** | **40.3** | **20.3** | **46.7** | **17.3 (7.8, 24.6)** |
| Gauteng | Sedibeng | 129 | 66 | 41 | 22 | 168 | 88 | 55 | 25 | 154 | 52 | 53 | 49 | 166 | 83 | 26 | 57 | 16.6 (5.4, 25.7) |
|  | West Rand | 135 | 76 | 40 | 19 | 177 | 113 | 38 | 26 | 155 | 60 | 46 | 49 | 178 | 99 | 25 | 54 | 17 (5.8, 26.7) |
|  | Ekurhuleni | 123 | 71 | 31 | 21 | 143 | 96 | 25 | 22 | 128 | 66 | 21 | 41 | 101 | 33 | 13 | 55 | 16.4 (5.6, 25.2) |
|  | City of Johannesburg | 128 | 74 | 36 | 18 | 154 | 105 | 25 | 24 | 130 | 58 | 29 | 43 | 117 | 50 | 14 | 53 | 16.5 (5.6, 25.4) |
|  | City of Tshwane | 113 | 69 | 27 | 17 | 102 | 68 | 14 | 20 | 119 | 68 | 26 | 25 | 87 | 28 | 24 | 35 | 18.2 (7.4, 27) |
|  | **Total** (average) | **125.6** | **71.2** | **35** | **19.4** | **148.8** | **94** | **31.4** | **23.4** | **137.2** | **60.8** | **35** | **41.4** | **129.8** | **58.6** | **20.4** | **50.8** | **16.9 (6, 26)** |
| Kwazulu  Natal | Ugu | 86 | 45 | 20 | 21 | 92 | 53 | 30 | 9 | 88 | 45 | 43 | 0 | 83 | 59 | 24 | 0 | 18.3 (11.8, 24.5) |
|  | UMgungundlovu | 90 | 40 | 29 | 21 | 110 | 63 | 22 | 25 | 129 | 77 | 39 | 13 | 137 | 78 | 56 | 3 | 15.5 (6.8, 22.2) |
|  | Uthukela | 102 | 45 | 28 | 29 | 128 | 59 | 36 | 33 | 140 | 80 | 19 | 41 | 135 | 72 | 13 | 50 | 15.8 (5.8, 23.1) |
|  | Umzinyathi | 106 | 53 | 23 | 30 | 145 | 64 | 48 | 33 | 167 | 87 | 35 | 45 | 138 | 47 | 40 | 51 | 17.3 (8.4, 24.3) |
|  | Amajuba | 123 | 57 | 32 | 34 | 142 | 52 | 42 | 48 | 172 | 81 | 38 | 53 | 180 | 86 | 37 | 57 | 15.9 (6.1, 22.8) |
|  | Zululand | 92 | 51 | 22 | 19 | 84 | 39 | 21 | 24 | 96 | 51 | 13 | 32 | 83 | 34 | 30 | 19 | 18.3 (10.2, 24.6) |
|  | Umkhanyakude | 91 | 50 | 28 | 13 | 81 | 41 | 14 | 26 | 80 | 37 | 16 | 27 | 84 | 36 | 27 | 21 | 22 (15.8, 27) |
|  | Uthungulu | 88 | 49 | 24 | 15 | 112 | 65 | 36 | 11 | 117 | 75 | 42 | 0 | 117 | 76 | 40 | 1 | 19.8 (12.8, 25.9) |
|  | iLembe | 98 | 56 | 26 | 16 | 114 | 64 | 38 | 12 | 129 | 88 | 41 | 0 | 118 | 85 | 33 | 0 | 19.5 (12.6, 25.8) |
|  | Sisonke | 92 | 47 | 31 | 14 | 109 | 57 | 30 | 22 | 117 | 73 | 31 | 13 | 128 | 91 | 37 | 0 | 14.2 (4.6, 21.1) |
|  | eThekwini | 97 | 59 | 18 | 20 | 103 | 62 | 29 | 12 | 135 | 100 | 33 | 2 | 126 | 97 | 29 | 0 | 19.3 (12.7, 25.1) |
|  | **Total** (average) | **96.8** | **50.18** | **25.5** | **21.1** | **110.9** | **56.3** | **31.5** | **23.2** | **124.5** | **72.2** | **31.8** | **20.5** | **120.8** | **69.2** | **33.3** | **18.4** | **17.8 (9.8, 24.2)** |
| Country | District  municipality | SPEI1 (weeks) | | | | SPEI6 (weeks) | | | | SPEI12 (weeks) | | | | SPEI5 (weeks) | | | | Tmean (°C);  (min, max) |
|  |  | D | I | II | III | D | I | II | III | D | I | II | III | D | I | II | III |  |
| **South Africa** | **Overall (52)** | **103** | **54 (52.4%)** | **28 (27.2%)** | **20 (19.4%)** | **119** | **65 (54.6%)** | **32 (26.9%)** | **22 (18.5%)** | **126** | **61 (48.4%)** | **38 (30.2%)** | **27 (21.4%)** | **122** | **59 (48.4%)** | **32 (26.2%)** | **31 (25.4%)** | **17.8**  **(7.6, 26.9)** |

**Table S5**. Overall drought-related mortality association (relative risks (RR), and 95% confidence intervals) by types of droughts based on its accumulation period (short-, medium-, long-, very long- term droughts) in South Africa between 2009 and 2016. Results are indicated for the total population and for each group of sex and age, and cause of mortality. SPEI,1,6,12,15= Standardized Precipitation Evapotranspiration Index measured at 1,6,12, and 15 months of accumulation, respectively. A00-B00= mortality due to certain infectious and parasitic diseases; E00-E90= mortality due to endocrine, metabolic, and nutritional causes; I00-I99= mortality due to circulatory conditions; J00-J99= mortality due to respiratory conditions.

| **Groups of study** | **SPEI1** | **SPEI6** | **SPEI12** | **SPEI15** |
| --- | --- | --- | --- | --- |
| All ages (all-cause mortality) | RR: 1.027 [1.018, 1.036] | RR: 1.035 [1.021, 1.050] | RR: 1.033 [1.008, 1.058] | RR: 1.098 [1.068, 1.129] |
| Children (0-4 years) | RR: 1.032 [1.01, 1.055] | RR: 1.038 [1.000, 1.078] | RR: 1.027 [0.951, 1.108] | RR: 1.124 [1.048, 1.207] |
| Pre-adults (5-24 years) | RR: 1.032 [1.009, 1.055] | RR: 1.027 [0.99, 1.065] | RR: 1.016 [0.96, 1.075] | RR: 1.106 [1.021, 1.199] |
| Younger adults (25-44 years) | RR: 1.047 [1.034, 1.06] | RR: 1.037 [1.016, 1.058] | RR: 1.032 [1.002, 1.064] | RR: 1.106 [1.062, 1.151] |
| Middle-aged adults  (45-64 years) | RR: 1.034 [1.022, 1.047] | RR: 1.046 [1.028, 1.066] | RR: 1.038 [1.010, 1.068] | RR: 1.089 [1.051, 1.128] |
| The elderly (65-105) | RR: 1.02 [1.008, 1.032] | RR: 1.041 [1.023, 1.059] | RR: 1.054 [1.027, 1.083] | RR: 1.103 [1.069, 1.139] |
| Males | RR: 1.034 [1.023, 1.045] | RR: 1.034 [1.018, 1.051] | RR: 1.032 [1.003, 1.062] | RR: 1.095 [1.061, 1.131] |
| Females | RR: 1.021 [1.01, 1.031] | RR: 1.037 [1.021, 1.053] | RR: 1.034 [1.010, 1.059] | RR: 1.1 [1.065, 1.135] |
| A00-B99 | RR: 1.013 [0.998, 1.029] | RR: 1.016 [0.993, 1.040] | RR: 0.993 [0.955, 1.033] | RR: 1.037 [0.995, 1.08] |
| E00-E90 | RR: 1.01 [0.987, 1.032] | RR: 1.058 [1.023, 1.094] | RR: 1.010[0.960, 1.061] | RR: 1.09 [1.023, 1.161] |
| I00-I99 | RR: 1.032 [1.019, 1.046] | RR: 1.035 [1.015, 1.056] | RR: 1.051 [1.018, 1.086] | RR: 1.097 [1.052, 1.144] |
| J00-J99 | RR: 1.037 [1.021, 1.053] | RR: 1.059 [1.035, 1.083] | RR: 1.07 [1.026, 1.115] | RR: 1.189 [1.140, 1.241] |

**Table S6.** Coefficients, relative risks (RRs), 95% confidence intervals, and p-values obtained in the metanalytical model for A) different levels of urbanicity (“Other” and “Urban) using as reference the “Rural” level, and B) a multidimensional poverty index (MPI) in South Africa. * p<0.05; # tendency to be significant (p<0.07).

| **A) Urbanicity level** | ALL | <5 | 5-24 | 25-44 | 45-64 | ≥65 | males | females | A00B99 | E00E90 | I00I99 | J00J99 |
| --- | --- | --- | --- | --- | --- | --- | --- | --- | --- | --- | --- | --- |
| SPEI1 | | | | | | | | | | | | |
| **«Rural»**  Coefficients  RRs [95%CI | Category of reference | Category of reference | Category of reference | Category of reference | Category of reference | Category of reference | Category of reference | Category of reference | Category of reference | Category of reference | Category of reference | Category of reference |
|  | 1.026  [0.751, 1.402] | 0.996  [0.996, 0.996] | 1.034  [0.724, 1.477] | 1.055  [0.671, 1.657] | 1.034  [0.724, 1.476] | 1.021  [0.769, 1.357] | 1.03  [0.735, 1.444] | 1.023  [0.762, 1.373] | 1.009  [0.842, 1.208] | 1.015  [0.797, 1.294] | 1.023  [0.761, 1.376] | 1.039  [0.707, 1.527] |
| **«Other»**  Coefficients  RRs [95%CI] | -0.0003  [-0.022, 0.022]  p=0.981 | 0.056  [0.003, 0.110]*  p=0.040 | -0.004  [-0.057, 0.050]  p=0.891 | -0.015  [-0.046, 0.016]  p=0.348 | -0.005  [-0.036, 0.026]  p=0.737 | -0.003  [-0.031, 0.025]  p=0.848 | 0.003  [-0.022, 0.029]  p=0.799 | -0.005  [-0.030, 0.020]  p=0.675 | 0.003  [-0.036, 0.041]  p= 0.881 | -0.010  [-0.064, 0.045]  p=0.725 | 0.004  [-0.029, 0.038]  p=0.975 | 0.004  [-0.035, 0.043]  p=0.832 |
|  | 1.025  [0.752, 1.399] | 1.054  [0.672, 1.653] | 1.03  [0.736, 1.441] | 1.039  [0.708, 1.524] | 1.028  [0.742, 1.425] | 1.018  [0.781, 1.327] | 1.034  [0.724, 1.476] | 1.017  [0.787, 1.316] | 1.011  [0.82, 1.247] | 1.005  [0.87, 1.162] | 1.028  [0.743, 1.421] | 1.044  [0.696m 1.566] |
| **«Urban»**  Coefficients  RRs [95%CI] | 0.008  [-0.017, 0.033]  p=0.537 | 0.048  [-0.007, 0.103]  p=0.089 | -0.001  [-0.054, 0.052]  p=0.969 | -0.004  [-0.037, 0.029]  p=0.820 | 0.014  [-0.019, 0.047]  p=0.417 | 0.0001  [-0.030, 0.030]  P=0.997 | 0.012  [-0.016, 0.041]  p=0.403 | 0.002  [-0.026, 0.029]  p=0.906 | 0.015  [-0.026, 0.056]  p=0.468 | -0.006  [-0.062, 0.049]  p=0.819 | 0.027  [-0.007, 0.060]  p=0.122 | -0.018  [-0.058, 0.023]  p=0.391 |
|  | 1.034  [0.723, 1.479] | 1.045  [0.692, 1.58] | 1.033  [0.727, 1.467] | 1.051  [0.68, 1.624] | 1.048  [0.686, 1.602] | 1.021  [0.768, 1.357] | 1.043  [0.698, 1.557] | 1.025  [0.755, 1.39] | 1.024  [0.757, 1.385] | 1.009  [0.839, 1.213] | 1.051  [0.68, 1.624] | 1.021  [0.769, 1.356] |
| SPEI6 | | | | | | | | | | | | |
| **«Rural»**  Coefficients  RRs [95%CI | Category of reference | Category of reference | Category of reference | Category of reference | Category of reference | Category of reference | Category of reference | Category of reference | Category of reference | Category of reference | Category of reference | Category of reference |
|  | 1.04  [0.707, 1.529] | 1.067  [0.648, 1.757] | 1.049  [0.683, 1.612] | 1.038  [0.711, 1.516] | 1.053  [0.674, 1.645] | 1.05  [0.682, 1.617] | 1.045  [0.692, 1.58] | 1.037  [0.714, 1.505] | 0.999  [0.999, 0.999] | 1.047  [0.689, 1.591] | 1.031  [0.733, 1.449] | 1.058  [0.665, 1.683] |
| **«Other»**  Coefficients  RRs [95%CI] | -0.005  [-0.039, 0.029]  p=0.761 | -0.032  [-0.129, 0.064]  p=0.515 | -0.049  [-0.140, 0.041]  p=0.284 | 0.008  [-0.041, 0.057]  p=0.752 | -0.012  [-0.058, 0.033]  p=0.605 | -0.010  [-0.052, 0.033]  p=0.664 | -0.014  [-0.054, 0.026]  p=0.483 | 0.001  [-0.038, 0.040]  p=0.968 | 0.025  [-0.033, 0.082]  p=0.398 | -0.010  [-0.093, 0.074]  p=0.820 | 0.010  [-0.041, 0.061]  p=0.693 | 0.010  [-0.049, 0.069]  p=0.734 |
|  | 1.034  [0.722, 1.48] | 1.033  [0.725, 1.473] | 0.999  [0.999, 0.999] | 1.046  [0.689, 1.588] | 1.041  [0.704, 1.538] | 1.04  [0.706, 1.532] | 1.031  [0.734, 1.448] | 1.038  [0.712, 1.512] | 1.024  [0.757, 1.386] | 1.037  [0.715, 1.503] | 1.041  [0.702, 1.545] | 1.069  [0.645, 1.771] |
| **«Urban»**  Coefficients  RRs [95%CI] | -0.008  [-0.049, 0.032]  p=0.688 | -0.054  [-0.156, 0.047]  p=0.294 | 0.001  [-0.094, 0.096]  p=0.978 | -0.022  [-0.076, 0.031]  p=0.414 | -0.003  [-0.052, 0.047 ]  p=0.909 | -0.021  [-0.068, 0.026]  p=0.388 | -0.019  [-0.065, 0.027]  p=0.414 | -0.001  [-0.045, 0.043]  p=0.952 | 0.025  [-0.037, 0.087]p=0.425 | 0.066  [-0.021, 0.154]  p=0.139 | -0.001  [-0.055, 0.053]  p=0.974 | -0.016  [-0.079, 0.047]  p=0.617 |
|  | 1.031  [0.732, 1.451] | 1.011  [0.827, 1.235] | 1.051  [0.68, 1.623] | 1.015  [0.799, 1.29] | 1.05  [0.681, 1.619] | 1.028  [0.742, 1.425] | 1.026  [0.751, 1.401] | 1.035  [0.718, 1.492] | 1.025  [0.755, 1.391] | 1.118  [0.581, 2.153] | 1.03  [0.736, 1.44] | 1.041  [0.703, 1.541] |
| SPEI12 | | | | | | | | | | | | |
| **«Rural»**  Coefficients  RRs [95%CI | Category of reference | Category of reference | Category of reference | Category of reference | Category of reference | Category of reference | Category of reference | Category of reference | Category of reference | Category of reference | Category of reference | Category of reference |
|  | 1.049  [0.682, 1.614] | 0.978  [0.978, 0.978] | 1.056  [0.669, 1.666] | 1.029  [0.738, 1.436] | 1.075  [0.634, 1.824] | 1.072  [0.64, 1.795] | 1.055  [0.671, 1.659] | 1.046  [0.691, 1.584] | 1.03  [0.734, 1.446] | 1.054  [0.671, 1.656] | 1.047  [0.687, 1.596] | 1.006  [0.861, 1.176] |
| **«Other»**  Coefficients  RRs [95%CI] | -0.031  [-0.090, 0.028]  p=0.299 | 0.077  [-0.116, 0.269]  p=0.434 | -0.068  [-0.209, 0.074]  p=0.348 | -0.001  [-0.079, 0.078]  p=0.986 | -0.061  [-0.132, 0.010]  p=0.091 | -0.024  [-0.093, 0.045]  p=0.499 | -0.044  [-0.115, 0.027]  p=0.228 | -0.019  [-0.078, 0.041]  p=0.535 | -0.072  [-0.170, 0.025]  p=0.145 | -0.066  [-0.194, 0.062]  p=0.312 | 0.005  [-0.082, 0.091]  p=0.913 | 0.079  [-0.027, 0.185]  p=0.146 |
|  | 1.017  [0.788, 1.313] | 1.056  [0.668, 1.669] | 0.987  [0.987, 0.987] | 1.029  [0.74, 1.429] | 1.011  [0.821, 1.246] | 1.047  0.689, 1.59] | 1.01  [0.834, 1.222] | 1.026  [0.748, 1.408] | 0.958  [0.958, 0.958] | 0.987  [0.987, 0.987] | 1.052  [0.676, 1.638] | 1.089  [0.615, 1.928] |
| **«Urban»**  Coefficients  RRs [95%CI] | -0.001  [-0.069, 0.068]  p=0.981 | 0.053  [-0.155, 0.262]  p=0.616 | -0.029  [-0.173, 0.115]  p=0.692 | 0.013  [-0.069, 0.095]  p=0.754 | -0.020  [-0.095, 0.055]  p=0.607 | -0.019  [-0.093, 0.055]  p=0.609 | 0.001  [-0.080, 0.082]  p=0.978 | -0.007  [-0.072, 0.057]  p=0.823 | -0.009  [-0.113, 0.094]  p=0.861 | -0.051  [-0.182, 0.080]  p=0.449 | 0.005  [-0.086, 0.097]  p=0.908 | 0.0982  [-0.015, 0.211]  p=0.088 |
|  | 1.049  [0.684, 1.606] | 1.032  [0.73, 1.457] | 1.025  [0.752, 1.398] | 1.043  [0.698, 1.559] | 1.054  [0.672, 1.655] | 1.051  [0.678, 1.629] | 1.056  [0.668, 1.669] | 1.038  [0.71, 1.517] | 1.021  [0.771, 1.352] | 1.002  [0.911, 1.103] | 1.053  [0.675, 1.643] | 1.11  [0.589, 2.092] |
| SPEI15 | | | | | | | | | | | | |
| **«Rural»**  Coefficients  RRs [95%CI] | Category of reference | Category of reference | Category of reference | Category of reference | Category of reference | Category of reference | Category of reference | Category of reference | Category of reference | Category of reference | Category of reference | Category of reference |
|  | 1.12  [0.579, 2.167] | 1.152  [0.551, 2.41] | 1.311  [0.473, 3.638] | 1.142  [0.559, 2.332] | 1.093  [0.609, 1.961] | 1.124  [0.575, 2.195] | 1.125  [0.574, 2.205] | 1.122  [0.577, 2.182] | 1.052  [0.676, 1.637] | 1.175  [0.535, 2.578] | 1.093  [0.609, 1.959] | 1.156  [0.548, 2.438] |
| **«Other»**  Coefficients  [96%CI]  RRs [95%CI] | -0.026  [-0.095, 0.042]  p=0.452 | -0.020  [-0.202, 0.161]  p=0.826 | -0.250  [-0.419, -0.081]*  p=0.004 | -0.054  [-0.154, 0.047]  p=0.294 | 0.004  [-0.087, 0.094]  p=0.939 | -0.026  [-0.106, 0.054]  p=0.529 | -0.036  [-0.115, 0.043]  p=0.372 | -0.028  [-0.107, 0.051]  p=0.485 | -0.035  [-0.139, 0.068]  p=0.502 | -0.135  [-0.299, 0.029]  p=0.106 | 0.013  [-0.096, 0.122]  p=0.814 | 0.061  [-0.052, 0.173]  p=0.291 |
|  | 1.091  [0.612, 1.945] | 1.129  [0.57, 2.235] | 1.021  [0.769, 1.357] | 1.082  [0.624, 1.878] | 1.097  [0.604, 1.99] | 1.095  [0.606, 1.978] | 1.085  [0.619, 1.902] | 1.091  [0.612, 1.946] | 1.016  [0.796, 1.296] | 1.026  [0.75, 1.403] | 1.107  [0.592, 2.069] | 1.228  [0.505, 2.986] |
| **«Urban»**  Coefficients  [96%CI]  RRs [95%CI] | -0.036  [-0.116, 0.044]  p=0.379 | -0.055  [-0.233, 0.122]  p=0.542 | -0.267  [-0.433, -0.101]*  p=0.002 | -0.034  [-0.139, 0.072]  p=0.531 | -0.022  [-0.119, 0.075]  p=0.653 | -0.025  [-0.110, 0.059]  p=0.556 | -0.046  [-0.133, 0.042]  p=0.305 | -0.032  [-0.118, 0.055]  p=0.475 | 0.003  [-0.099, 0.105]  p=0.953 | -0.063  [-0.221, 0.095]  p=0.436 | -0.008  [-0.125, 0.108]  p=0.887 | 0.003  [-0.109, 0.114]  p=0.961 |
|  | 1.081  [0.626, 1.865] | 1.09  [0.613, 1.94] | 1.004  [0.887, 1.136] | 1.104  [0.596, 2.046] | 1.069  [0.644, 1.773] | 1.095  [0.606, 1.979] | 1.075  [0.635, 1.818] | 1.087  [0.617, 1.915] | 1.055  [0.669, 1.664] | 1.103  [0.597, 2.037] | 1.084  [0.622, 1.889] | 1.159  [0.546, 2.462] |

| **B) Multidimensional poverty Index (MPDI)**  **(Continuous variable)** | ALL | <5 | 5-24 | 25-44 | 45-64 | ≥65 | males | females | A00B99 | E00E90 | I00I99 | J00J99 |
| --- | --- | --- | --- | --- | --- | --- | --- | --- | --- | --- | --- | --- |
| SPEI1 | | | | | | | | | | | | |
| Coefficients [95% CI] | -0.154  [-0.595,0.288 ]  p=0.495 | -0.497  [-1.571, 0.578]  p=0.365 | -0.276  [-1.247, 0.695]  p=0.577 | -0.075  [-0.681, 0.530]  p=0.807 | -0.184  [-0.786, 0.417]  p=0.548 | 0.045  [-0.500, 0.590]  p=0.872 | -0.201  [-0.707, 0.306]  p=0.438 | -0.082  [-0.576, 0.412]  p=0.746 | -0.041  [-0.808, 0.727]  p=0.918 | 0.056  [-1.076, 1.188]  p=0,923 | -0.243  [-0.909, 0.424]  p=0.476 | 0.211  [-0.584, 1.006]  p=0,603 |
| RRs [95%CI]  Minimum value of the MPDI | 1.031  [0.731, 1.456] | 1.046  [0.69, 1.587] | 1.04  [0.706, 1.531] | 1.049  [0.683, 1.611] | 1.039  [0.707, 1.527] | 1.019  [0.78, 1.331] | 1.04  [0.706, 1.532] | 1.023  [0.761, 1.374] | 1.014  [0.803, 1.282] | 1.008  [0.845, 1.202] | 1.038  [0.711, 1.517] | 1.031  [0.731, 1.454] |
| RRs [95%CI]  Maximum value of the MPDI | 1.018  [0.784, 1.322] | 1.003  [0.906, 1.11] | 1.015  [0.797, 1.294] | 1.042  [0.699, 1.553] | 1.023  [0.761, 1.375] | 1.023  [0.763, 1.371] | 1.022  [0.765, 1.367] | 1.016  [0.795, 1.299] | 1.011  [0.825, 1.239] | 1.013  [0.811, 1.265] | 1.017  [0.79, 1.309] | 1.05  [0.681, 1.619] |
| SPEI6 | | | | | | | | | | | | |
| Coefficients [95% CI] | -0.115  [-0.825, 0.594]  p=0.750 | 0.020  [-2.095, 2.134]  p=0.986 | 0.326  [-1.414, 2.065]  p=0.714 | 0.097  [-0.898, 1.093]  p=0.848 | -0.156  [-1.077, 0.766]  p=0.741 | 0.278  [-0.589, 1.146]  P=0.530 | 0.018  [-0.797, 0.833]  p=0.966 | -0.149  [-0.944, 0.646]  p=0.714 | -0.652  [-1.815, 0.511]  p=0.272 | -1.517  [-3.327, 0.293]  p=0.100 | 0.014  [-1.047, 1.075]  p=0.980 | -0.272  [-1.524, 0.979]  p=0.670 |
| RRs [95%CI]  Minimum value of the MPDI | 1.038  [0.71, 1.519] | 1.038  [0.712, 1.513] | 1.018  [0.783, 1.325] | 1.034  [0.722, 1.481] | 1.051  [0.68, 1.623] | 1.033  [0.725, 1.473] | 1.034  [0.723, 1.479] | 1.041  [0.703, 1.541] | 1.034  [0.723, 1.478] | 1.1  [0.601, 2.012] | 1.035  [0.719, 1.489] | 1.066  [0.65, 1.749] |
| RRs [95%CI]  Maximum value of the MPDI | 1.028  [0.741, 1.426] | 1.039  [0.707, 1.529] | 1.047  [0.688, 1.593] | 1.043  [0.698, 1.558] | 1.037  [0.715, 1.503] | 1.058  [0.664, 1.686] | 1.036  [0.718, 1.494] | 1.028  [0.743, 1.422] | 0.978  [0.978, 0.978] | .0.966  [0.966, 0.966] | 1.036  [0.716, 1.5] | 1.041  [0.702, 1.545] |
| SPEI12 | | | | | | | | | | | | |
| Coefficients [95% CI] | 0.703  [-0.514, 1.921]  p=0.703 | -1.682  [-5.524, 2.160]  p=0.391 | 1.060  [-1.651, 3.771]  p=0.444 | -0.01  [-1.567, 1.548]  P=0.990 | 1.230  [-0.196, 2.655]  P=0.091 | 1.620  [0.283, 2.956]  p=0.018* | 0.767  [-0.699, 2.233]  p=0.305 | 0.731  [-0.487, 1.950]  p=0.240 | 0.072  [-1.966, 2.110]  p=0.945 | 0.398  [-2.393, 3.189]  p=0.780 | 0.973  [-0.767, 2.713]  p=0.273 | -1.804  [-4.050, 0.442]  p=0.619 |
| RRs [95%CI]  Minimum value of the MPDI | 1.015  [0.802, 1.284] | 1.073  [0.638, 1.806] | 0.989  [0.989, 0.989] | 1.033  [0.727, 1.467] | 1.009  [0.837, 1.217] | 1.014  [0.803, 1.282] | 1.012  [0.815, 1.258] | 1.016  [0.73, 1.303] | 0.991  [0.991, 0.991] | 1  [1-1] | 1.029  [0.74, 1.431] | 1.117  [0.582, 2.146] |
| RRs [95%CI]  Maximum value of the MPDI | 1.077  [0.631, 1.841] | 0.929  [0.929, 0.929] | 1.083  [0.622, 1.887] | 1.032  [0.729, 1.459] | 1.121  [0.578, 2.175] | 1.165  [0.541, 2.507] | 1.081  [0.625, 1.869] | 1.082  [0.624, 1.875] | 0.997  [0.997, 0.997] | 1.035  [0.721, 1.486] | 1.118  [0.581, 2.152] | 0.957  [0.957, 0.957] |
| SPEI15 | | | | | | | | | | | | |
| Coefficients [95% CI] | 1.187  [-0.055, 2.428]  p=0.061# | 0.257  [-2.954, 3.467]  p=0.875 | 1.856  [-1.560, 5.272]  p=0.287 | 1.489  [-0.234, 3.212]  p=0.090 | 0.988  [-0.629, 2.606]  p=0.231 | 1.301  [-0.103, 2.705]  p=0.069# | 0.875  [-0.561, 2.312]  p=0.232 | 1.694  [0.355, 3.034]  p=0.013* | 1.000  [-0.832, 2.833]  p=0.285 | 1.287  [-1.770, 4.344]  p=0.409 | 1.418  [-0.580, 3.415]  p=0.164 | 0.381  [-1.684, 2.446]  p=0.718 |
| RRs [95%CI]  Minimum value of the MPDI | 1.066  [0.65, 1.746] | 1.117  [0.582, 2.144] | 1.053  [0.675, 1.641] | 1.064  [0.652, 1.736] | 1.063  [0.654, 1.729] | 1.069  [0.644, 1.777] | 1.072  [0.64, 1.794] | 1.053  [0.674, 1.644] | 1.011  [0.821, 1.246] | 1.056  [0.668, 1.669] | 1.061  [0.658, 1.713] | 1.179  [0.532, 2.61] |
| RRs [95%CI]  Maximum value of the MPDI | 1.179  [0.532, 2.615] | 1.142  [0.559, 2.332] | 1.234  [0.502, 3.031] | 1.209  [0.515, 2.84] | 1.157  [0.547, 2.448] | 1.195  [0.522, 2.736] | 1.155  [0.549, 2.43] | 1.217  [0.51, 2.904] | 1.102  [0.598, 2.029] | 1.179  [0.532, 2.612] | 1.198  [0.521, 2.759] | 1.218  [0.51, 2.907] |

**Table S7**. Relative risks of mortality and 95% confidence intervals associated with short-term droughts (SPEI measured at one month of accumulation, SPEI1) by district municipality in South Africa between 2009 and 2016. Short-term droughts were mostly linked to a higher number of positive associations and robust estimates in the populations of the city of Tshwane (Gauteng), Nkangala, and Gert Sibande (Mpumalanga), where short-term droughts were relatively frequent and/or severe (Table S4).

| SPEI1 | all | <5 years | 5-24 years | 25-44 years | 45-64 years | 65-105 years | males | females | A00B99 | E00E90 | I00I99 | J00J99 |
| --- | --- | --- | --- | --- | --- | --- | --- | --- | --- | --- | --- | --- |
| Alfred Nzo | 1.023 [0.96 - 1.09] | 1.074 [0.938 - 1.229] | 1.023 [0.943 - 1.11] | 1.066 [0.977 - 1.164] | 0.981 [0.899 - 1.071] | 1.049 [0.973 - 1.131] | 1.001 [0.93 - 1.076] | 0.964 [0.852 - 1.092] | 1.067 [0.852 - 1.337] | 1.154 [0.999 - 1.333] | 1.023 [0.96 - 1.09] | 1.074 [0.938 - 1.229] |
| Amajuba | 0.994 [0.936 - 1.057] | 0.976 [0.837 - 1.137] | 0.981 [0.897 - 1.074] | 1.067 [0.978 - 1.164] | 0.93 [0.851 - 1.017] | 1.014 [0.941 - 1.093] | 0.975 [0.911 - 1.045] | 1.038 [0.933 - 1.155] | 1.001 [0.86 - 1.165] | 0.943 [0.856 - 1.038] | 0.994 [0.936 - 1.057] | 0.976 [0.837 - 1.137] |
| Amathole | 1.041 [0.984 - 1.1] | 1.044 [0.927 - 1.177] | 1.061 [0.983 - 1.145] | 1.069 [0.998 - 1.145] | 1.027 [0.967 - 1.091] | 1.044 [0.98 - 1.112] | 1.037 [0.977 - 1.101] | 1.037 [0.959 - 1.121] | 1.038 [0.926 - 1.164] | 1.008 [0.937 - 1.086] | 1.041 [0.984 - 1.1] | 1.044 [0.927 - 1.177] |
| Bojanala | 1.031 [0.966 - 1.1] | 0.983 [0.844 - 1.145] | 1.045 [0.955 - 1.144] | 0.974 [0.901 - 1.053] | 1.049 [0.972 - 1.132] | 1.027 [0.954 - 1.106] | 1.032 [0.964 - 1.106] | 1.031 [0.943 - 1.126] | 1.104 [0.967 - 1.261] | 1.024 [0.945 - 1.11] | 1.031 [0.966 - 1.1] | 0.983 [0.844 - 1.145] |
| Buffalo City | 1.014 [0.947 - 1.087] | 0.985 [0.838 - 1.158] | 1.018 [0.929 - 1.114] | 1.028 [0.943 - 1.121] | 1.019 [0.937 - 1.108] | 0.983 [0.908 - 1.064] | 1.048 [0.971 - 1.132] | 1.034 [0.946 - 1.13] | 1.009 [0.844 - 1.207] | 1.029 [0.935 - 1.132] | 1.014 [0.947 - 1.087] | 0.985 [0.838 - 1.158] |
| Cacadu | 1.043 [0.979 - 1.11] | 0.986 [0.831 - 1.171] | 1.024 [0.929 - 1.127] | 1.058 [0.968 - 1.156] | 1.052 [0.965 - 1.146] | 1.075 [0.997 - 1.16] | 1.012 [0.94 - 1.09] | 1.022 [0.918 - 1.137] | 1.123 [0.944 - 1.337] | 1.02 [0.931 - 1.118] | 1.043 [0.979 - 1.11] | 0.986 [0.831 - 1.171] |
| Cape Winelands | 1.051 [0.986 - 1.12] | 1.015 [0.833 - 1.237] | 1.091 [0.982 - 1.212] | 1.064 [0.982 - 1.152] | 1.025 [0.946 - 1.112] | 1.067 [0.992 - 1.149] | 1.033 [0.956 - 1.116] | 0.971 [0.866 - 1.089] | 1.135 [0.916 - 1.407] | 1.032 [0.942 - 1.131] | 1.051 [0.986 - 1.12] | 1.015 [0.833 - 1.237] |
| Capricorn | 1.016 [0.943 - 1.095] | 0.987 [0.829 - 1.176] | 1.026 [0.926 - 1.137] | 0.997 [0.907 - 1.097] | 1.02 [0.935 - 1.112] | 1.022 [0.934 - 1.117] | 1.012 [0.935 - 1.096] | 1.003 [0.903 - 1.115] | 0.938 [0.808 - 1.089] | 1.09 [0.976 - 1.217] | 1.016 [0.943 - 1.095] | 0.987 [0.829 - 1.176] |
| Central Karoo | 1.08 [0.94 - 1.24] | 1.387 [0.79 - 2.436] | 1.055 [0.806 - 1.38] | 1.014 [0.815 - 1.261] | 1.158 [0.945 - 1.418] | 1.162 [0.972 - 1.389] | 0.997 [0.829 - 1.198] | 0.981 [0.709 - 1.357] | 1.095 [0.563 - 2.131] | 1.146 [0.904 - 1.453] | 1.08 [0.94 - 1.24] | 1.387 [0.79 - 2.436] |
| Chris Hani | 1.012 [0.951 - 1.077] | 0.983 [0.851 - 1.134] | 1.042 [0.957 - 1.135] | 1.018 [0.936 - 1.108] | 1.014 [0.942 - 1.093] | 1.006 [0.937 - 1.08] | 1.017 [0.95 - 1.09] | 1.038 [0.945 - 1.141] | 1.014 [0.881 - 1.167] | 0.975 [0.894 - 1.064] | 1.012 [0.951 - 1.077] | 0.983 [0.851 - 1.134] |
| City of Cape Town | 1.014 [0.966 - 1.064] | 1.031 [0.935 - 1.136] | 1.013 [0.95 - 1.08] | 1.022 [0.969 - 1.077] | 1.022 [0.972 - 1.075] | 1.027 [0.975 - 1.082] | 0.997 [0.948 - 1.048] | 0.994 [0.93 - 1.063] | 1.018 [0.919 - 1.128] | 1.04 [0.984 - 1.099] | 1.014 [0.966 - 1.064] | 1.031 [0.935 - 1.136] |
| City of Johannesburg | 1.056 [0.997 - 1.118] | 0.997 [0.893 - 1.114] | 1.095 [1.022 - 1.173] | 1.058 [0.993 - 1.127] | 1.028 [0.965 - 1.096] | 1.06 [0.997 - 1.127] | 1.053 [0.991 - 1.119] | 1.034 [0.961 - 1.112] | 1.013 [0.895 - 1.147] | 1.067 [0.997 - 1.142] | 1.056 [0.997 - 1.118] | 0.997 [0.893 - 1.114] |
| City of Tshwane | 1.079 [1.018 - 1.144] | 1.118 [0.972 - 1.286] | 1.071 [0.992 - 1.155] | 1.105 [1.031 - 1.183] | 1.051 [0.985 - 1.121] | 1.102 [1.035 - 1.173] | 1.054 [0.99 - 1.123] | 1.148 [1.054 - 1.251] | 1.062 [0.946 - 1.193] | 1.077 [1.004 - 1.154] | 1.079 [1.018 - 1.144] | 1.118 [0.972 - 1.286] |
| Dr Kenneth Kaunda | 1.056 [0.987 - 1.129] | 1.008 [0.83 - 1.224] | 1.053 [0.952 - 1.164] | 1.046 [0.956 - 1.145] | 1.072 [0.981 - 1.172] | 1.046 [0.968 - 1.131] | 1.071 [0.99 - 1.16] | 1.082 [0.977 - 1.198] | 1.009 [0.832 - 1.222] | 1.061 [0.954 - 1.181] | 1.056 [0.987 - 1.129] | 1.008 [0.83 - 1.224] |
| Dr Ruth Segomotsi Mompati | 1.028 [0.96 - 1.1] | 1.089 [0.895 - 1.325] | 1.044 [0.949 - 1.149] | 0.992 [0.903 - 1.09] | 1.031 [0.945 - 1.124] | 1.052 [0.974 - 1.136] | 1 [0.924 - 1.082] | 1 [0.896 - 1.117] | 1.039 [0.868 - 1.244] | 0.978 [0.879 - 1.089] | 1.028 [0.96 - 1.1] | 1.089 [0.895 - 1.325] |
| Eden | 0.982 [0.923 - 1.046] | 1.062 [0.869 - 1.298] | 0.977 [0.879 - 1.085] | 0.957 [0.88 - 1.041] | 0.987 [0.911 - 1.069] | 0.986 [0.915 - 1.063] | 0.979 [0.911 - 1.054] | 0.909 [0.803 - 1.029] | 0.839 [0.645 - 1.09] | 0.945 [0.86 - 1.039] | 0.982 [0.923 - 1.046] | 1.062 [0.869 - 1.298] |
| Ehlanzeni | 1.008 [0.952 - 1.068] | 1.043 [0.922 - 1.179] | 1.061 [0.985 - 1.142] | 0.967 [0.901 - 1.038] | 0.979 [0.916 - 1.047] | 1.009 [0.944 - 1.078] | 1.008 [0.949 - 1.071] | 0.999 [0.924 - 1.081] | 1.024 [0.92 - 1.138] | 1.012 [0.934 - 1.096] | 1.008 [0.952 - 1.068] | 1.043 [0.922 - 1.179] |
| Ekurhuleni | 1.046 [0.988 - 1.108] | 1.062 [0.95 - 1.186] | 1.084 [1.013 - 1.16] | 1.06 [0.991 - 1.132] | 1.025 [0.96 - 1.095] | 1.052 [0.989 - 1.12] | 1.038 [0.978 - 1.103] | 1.039 [0.96 - 1.124] | 1.011 [0.907 - 1.128] | 1.047 [0.975 - 1.124] | 1.046 [0.988 - 1.108] | 1.062 [0.95 - 1.186] |
| eThekwini | 1.014 [0.967 - 1.063] | 1.004 [0.929 - 1.086] | 1.023 [0.965 - 1.084] | 1.036 [0.983 - 1.092] | 1.006 [0.952 - 1.063] | 1.022 [0.969 - 1.078] | 1.004 [0.956 - 1.054] | 1.01 [0.951 - 1.072] | 1.006 [0.921 - 1.099] | 1.004 [0.949 - 1.062] | 1.014 [0.967 - 1.063] | 1.004 [0.929 - 1.086] |
| Fezile Dabi | 1.051 [0.983 - 1.123] | 1.121 [0.906 - 1.387] | 1.03 [0.931 - 1.14] | 1.091 [0.998 - 1.193] | 1.009 [0.926 - 1.099] | 1.059 [0.976 - 1.148] | 1.042 [0.965 - 1.126] | 1.134 [1.004 - 1.28] | 0.892 [0.751 - 1.058] | 1.092 [0.992 - 1.201] | 1.051 [0.983 - 1.123] | 1.121 [0.906 - 1.387] |
| Frances Baard | 0.937 [0.863 - 1.018] | 0.933 [0.729 - 1.195] | 0.959 [0.845 - 1.089] | 0.923 [0.827 - 1.031] | 0.978 [0.869 - 1.101] | 0.918 [0.833 - 1.011] | 0.959 [0.864 - 1.065] | 0.93 [0.811 - 1.068] | 0.926 [0.732 - 1.172] | 1.031 [0.903 - 1.177] | 0.937 [0.863 - 1.018] | 0.933 [0.729 - 1.195] |
| Gert Sibande | 1.063 [1.003 - 1.128] | 1.034 [0.905 - 1.18] | 1.064 [0.984 - 1.151] | 1.105 [1.026 - 1.19] | 1.044 [0.972 - 1.121] | 1.087 [1.017 - 1.161] | 1.04 [0.973 - 1.111] | 0.992 [0.91 - 1.082] | 1.08 [0.958 - 1.218] | 1.045 [0.959 - 1.138] | 1.063 [1.003 - 1.128] | 1.034 [0.905 - 1.18] |
| Greater Sekhukhune | 1.023 [0.953 - 1.097] | 1.046 [0.886 - 1.234] | 1.019 [0.924 - 1.125] | 1.018 [0.923 - 1.123] | 1.042 [0.961 - 1.13] | 1.004 [0.923 - 1.091] | 1.044 [0.966 - 1.128] | 1.014 [0.916 - 1.122] | 1.033 [0.887 - 1.203] | 1.048 [0.949 - 1.156] | 1.023 [0.953 - 1.097] | 1.046 [0.886 - 1.234] |
| iLembe | 1.047 [0.988 - 1.11] | 1.069 [0.931 - 1.227] | 1.047 [0.967 - 1.134] | 1.082 [0.999 - 1.171] | 1.021 [0.942 - 1.105] | 1.027 [0.958 - 1.1] | 1.07 [0.999 - 1.146] | 1.079 [0.994 - 1.171] | 0.972 [0.839 - 1.126] | 1.042 [0.955 - 1.137] | 1.047 [0.988 - 1.11] | 1.069 [0.931 - 1.227] |
| Joe Gqabi | 1.035 [0.965 - 1.11] | 1.135 [0.94 - 1.37] | 1.014 [0.913 - 1.125] | 1.064 [0.962 - 1.176] | 1.034 [0.943 - 1.134] | 1.045 [0.963 - 1.133] | 1.025 [0.944 - 1.114] | 1.044 [0.931 - 1.171] | 1.064 [0.85 - 1.331] | 1.005 [0.887 - 1.138] | 1.035 [0.965 - 1.11] | 1.135 [0.94 - 1.37] |
| John Taolo Gaetsewe | 1.055 [0.957 - 1.163] | 1.132 [0.879 - 1.459] | 1.071 [0.925 - 1.239] | 1.082 [0.952 - 1.23] | 1.033 [0.895 - 1.192] | 1.037 [0.93 - 1.157] | 1.076 [0.951 - 1.216] | 1.099 [0.922 - 1.31] | 0.923 [0.675 - 1.263] | 1.009 [0.849 - 1.199] | 1.055 [0.957 - 1.163] | 1.132 [0.879 - 1.459] |
| Lejweleputswa | 1.038 [0.967 - 1.115] | 1.012 [0.85 - 1.205] | 1.065 [0.963 - 1.179] | 1.075 [0.988 - 1.17] | 0.966 [0.881 - 1.058] | 1.072 [0.989 - 1.163] | 0.999 [0.92 - 1.085] | 0.87 [0.771 - 0.981] | 1.083 [0.924 - 1.271] | 1.036 [0.929 - 1.155] | 1.038 [0.967 - 1.115] | 1.012 [0.85 - 1.205] |
| Mangaung | 1.045 [0.972 - 1.124] | 1.101 [0.923 - 1.313] | 1.085 [0.986 - 1.195] | 1.064 [0.968 - 1.169] | 1.011 [0.922 - 1.109] | 1.074 [0.991 - 1.164] | 1.013 [0.932 - 1.101] | 1.012 [0.907 - 1.129] | 0.9 [0.76 - 1.066] | 1.107 [0.993 - 1.233] | 1.045 [0.972 - 1.124] | 1.101 [0.923 - 1.313] |
| Mopani | 1.04 [0.976 - 1.108] | 0.977 [0.842 - 1.134] | 1.111 [1.016 - 1.215] | 1.06 [0.97 - 1.158] | 1.023 [0.949 - 1.102] | 1.02 [0.946 - 1.1] | 1.058 [0.988 - 1.132] | 1.042 [0.946 - 1.147] | 1.009 [0.889 - 1.145] | 1.018 [0.922 - 1.123] | 1.04 [0.976 - 1.108] | 0.977 [0.842 - 1.134] |
| Namakwa | 1.095 [0.97 - 1.234] | 0.661 [0.353 - 1.239] | 1.298 [1.022 - 1.649] | 1.073 [0.897 - 1.283] | 1.12 [0.944 - 1.329] | 1.086 [0.929 - 1.27] | 1.105 [0.941 - 1.296] | 1.108 [0.795 - 1.546] | 1.542 [0.993 - 2.395] | 1.145 [0.939 - 1.396] | 1.095 [0.97 - 1.234] | 0.661 [0.353 - 1.239] |
| Nelson Mandela Bay | 0.981 [0.908 - 1.06] | 0.988 [0.857 - 1.138] | 0.949 [0.863 - 1.042] | 1.011 [0.926 - 1.105] | 0.98 [0.903 - 1.064] | 0.992 [0.914 - 1.076] | 0.972 [0.896 - 1.054] | 0.926 [0.842 - 1.018] | 0.941 [0.806 - 1.099] | 1.067 [0.98 - 1.162] | 0.981 [0.908 - 1.06] | 0.988 [0.857 - 1.138] |
| Ngaka Modiri Molema | 1.048 [0.983 - 1.118] | 0.988 [0.835 - 1.169] | 1.094 [0.997 - 1.2] | 1.02 [0.941 - 1.106] | 1.026 [0.946 - 1.112] | 1.052 [0.976 - 1.134] | 1.043 [0.972 - 1.12] | 1.06 [0.959 - 1.172] | 0.945 [0.809 - 1.104] | 1.016 [0.927 - 1.115] | 1.048 [0.983 - 1.118] | 0.988 [0.835 - 1.169] |
| Nkangala | 1.063 [1 - 1.131] | 1.123 [0.953 - 1.323] | 1.055 [0.973 - 1.143] | 1.039 [0.96 - 1.125] | 1.103 [1.023 - 1.188] | 1.086 [1.01 - 1.167] | 1.038 [0.97 - 1.112] | 1.142 [1.039 - 1.256] | 1.042 [0.911 - 1.193] | 1.061 [0.97 - 1.16] | 1.063 [1 - 1.131] | 1.123 [0.953 - 1.323] |
| O.R.Tambo | 1.004 [0.948 - 1.063] | 1.006 [0.906 - 1.117] | 1.001 [0.93 - 1.078] | 1.021 [0.951 - 1.097] | 1.021 [0.955 - 1.092] | 1.023 [0.96 - 1.091] | 0.987 [0.928 - 1.049] | 0.948 [0.876 - 1.027] | 0.974 [0.84 - 1.128] | 1.06 [0.964 - 1.166] | 1.004 [0.948 - 1.063] | 1.006 [0.906 - 1.117] |
| Overberg | 1.067 [0.987 - 1.154] | 1.067 [0.806 - 1.412] | 1.122 [0.963 - 1.307] | 1.066 [0.95 - 1.195] | 1.054 [0.949 - 1.17] | 1.067 [0.967 - 1.177] | 1.071 [0.967 - 1.186] | 1.191 [0.995 - 1.425] | 0.689 [0.484 - 0.98] | 1.149 [1.02 - 1.293] | 1.067 [0.987 - 1.154] | 1.067 [0.806 - 1.412] |
| Pixley ka Seme | 1.013 [0.931 - 1.103] | 1.137 [0.885 - 1.461] | 1.074 [0.951 - 1.214] | 1.007 [0.896 - 1.133] | 0.994 [0.884 - 1.118] | 1.002 [0.911 - 1.102] | 1.024 [0.922 - 1.137] | 0.997 [0.862 - 1.153] | 1.119 [0.874 - 1.432] | 1.11 [0.981 - 1.255] | 1.013 [0.931 - 1.103] | 1.137 [0.885 - 1.461] |
| Sedibeng | 1.029 [0.967 - 1.095] | 1.035 [0.893 - 1.199] | 1.077 [0.986 - 1.177] | 1.042 [0.965 - 1.124] | 1 [0.925 - 1.082] | 1.019 [0.95 - 1.093] | 1.047 [0.976 - 1.124] | 0.989 [0.897 - 1.09] | 0.893 [0.764 - 1.045] | 1.005 [0.922 - 1.095] | 1.029 [0.967 - 1.095] | 1.035 [0.893 - 1.199] |
| Sisonke | 1.03 [0.967 - 1.097] | 1.019 [0.886 - 1.172] | 1.034 [0.944 - 1.132] | 1.01 [0.924 - 1.104] | 1.075 [0.988 - 1.169] | 1.012 [0.941 - 1.089] | 1.049 [0.973 - 1.13] | 0.97 [0.878 - 1.071] | 0.966 [0.824 - 1.131] | 1.053 [0.949 - 1.168] | 1.03 [0.967 - 1.097] | 1.019 [0.886 - 1.172] |
| Siyanda | 1.027 [0.94 - 1.122] | 0.998 [0.744 - 1.34] | 1.117 [0.978 - 1.276] | 0.985 [0.868 - 1.119] | 1.06 [0.933 - 1.204] | 0.994 [0.895 - 1.104] | 1.071 [0.96 - 1.194] | 0.907 [0.765 - 1.075] | 1.194 [0.878 - 1.625] | 1.065 [0.921 - 1.231] | 1.027 [0.94 - 1.122] | 0.998 [0.744 - 1.34] |
| Thabo Mofutsanyane | 1.043 [0.982 - 1.108] | 0.966 [0.84 - 1.111] | 1.112 [1.026 - 1.205] | 1.07 [0.992 - 1.153] | 1.021 [0.942 - 1.106] | 1.051 [0.979 - 1.127] | 1.035 [0.97 - 1.105] | 0.996 [0.91 - 1.091] | 1.032 [0.904 - 1.177] | 1.026 [0.942 - 1.119] | 1.043 [0.982 - 1.108] | 0.966 [0.84 - 1.111] |
| Ugu | 1.035 [0.983 - 1.09] | 1.077 [0.968 - 1.199] | 1.062 [0.991 - 1.138] | 1.064 [0.991 - 1.142] | 1.01 [0.947 - 1.077] | 1.053 [0.992 - 1.118] | 1.017 [0.961 - 1.076] | 1.043 [0.972 - 1.12] | 0.943 [0.834 - 1.066] | 1.006 [0.935 - 1.084] | 1.035 [0.983 - 1.09] | 1.077 [0.968 - 1.199] |
| UMgungundlovu | 1.034 [0.983 - 1.088] | 1.157 [1.033 - 1.296] | 1.016 [0.949 - 1.089] | 1.082 [1.018 - 1.151] | 1.017 [0.954 - 1.084] | 1.034 [0.973 - 1.099] | 1.037 [0.982 - 1.095] | 1.001 [0.924 - 1.086] | 0.993 [0.884 - 1.115] | 1.026 [0.961 - 1.096] | 1.034 [0.983 - 1.088] | 1.157 [1.033 - 1.296] |
| Umkhanyakude | 0.988 [0.918 - 1.062] | 1.011 [0.841 - 1.215] | 1.067 [0.963 - 1.182] | 0.947 [0.846 - 1.06] | 0.961 [0.868 - 1.064] | 1.022 [0.938 - 1.113] | 0.952 [0.874 - 1.037] | 0.971 [0.876 - 1.076] | 1.005 [0.815 - 1.239] | 1.02 [0.899 - 1.156] | 0.988 [0.918 - 1.062] | 1.011 [0.841 - 1.215] |
| Umzinyathi | 1.036 [0.976 - 1.101] | 0.95 [0.814 - 1.108] | 1.057 [0.966 - 1.158] | 1.047 [0.956 - 1.146] | 1.082 [1.001 - 1.17] | 1.045 [0.973 - 1.123] | 1.027 [0.958 - 1.1] | 1.096 [0.99 - 1.212] | 1.065 [0.913 - 1.242] | 1.065 [0.971 - 1.169] | 1.036 [0.976 - 1.101] | 0.95 [0.814 - 1.108] |
| Uthukela | 0.997 [0.946 - 1.052] | 1 [0.88 - 1.136] | 1.034 [0.959 - 1.115] | 0.971 [0.9 - 1.047] | 0.983 [0.918 - 1.053] | 0.994 [0.934 - 1.059] | 1.001 [0.941 - 1.065] | 0.996 [0.925 - 1.073] | 1.047 [0.922 - 1.189] | 0.95 [0.876 - 1.031] | 0.997 [0.946 - 1.052] | 1 [0.88 - 1.136] |
| Uthungulu | 0.994 [0.932 - 1.06] | 1.006 [0.863 - 1.172] | 0.985 [0.897 - 1.081] | 0.976 [0.897 - 1.063] | 1.042 [0.963 - 1.128] | 1.003 [0.929 - 1.082] | 0.988 [0.919 - 1.062] | 1.008 [0.912 - 1.113] | 1.035 [0.881 - 1.217] | 1.004 [0.907 - 1.111] | 0.994 [0.932 - 1.06] | 1.006 [0.863 - 1.172] |
| Vhembe | 0.988 [0.918 - 1.064] | 0.965 [0.804 - 1.158] | 1.033 [0.92 - 1.16] | 1.011 [0.915 - 1.117] | 0.949 [0.87 - 1.036] | 1.015 [0.929 - 1.108] | 0.965 [0.889 - 1.048] | 0.952 [0.837 - 1.083] | 0.936 [0.8 - 1.095] | 1.076 [0.953 - 1.216] | 0.988 [0.918 - 1.064] | 0.965 [0.804 - 1.158] |
| Waterberg | 1.048 [0.967 - 1.136] | 0.95 [0.752 - 1.2] | 1.086 [0.96 - 1.228] | 1.102 [0.982 - 1.236] | 0.985 [0.888 - 1.091] | 1.07 [0.973 - 1.177] | 1.025 [0.934 - 1.125] | 1.012 [0.885 - 1.157] | 1.081 [0.883 - 1.323] | 1.028 [0.912 - 1.158] | 1.048 [0.967 - 1.136] | 0.95 [0.752 - 1.2] |
| West Coast | 1 [0.931 - 1.075] | 1.492 [1.204 - 1.849] | 0.909 [0.795 - 1.04] | 0.992 [0.894 - 1.101] | 1.012 [0.928 - 1.105] | 0.998 [0.916 - 1.086] | 1.005 [0.92 - 1.097] | 0.871 [0.751 - 1.01] | 0.857 [0.65 - 1.129] | 1.029 [0.925 - 1.143] | 1 [0.931 - 1.075] | 1.492 [1.204 - 1.849] |
| West Rand | 1.044 [0.976 - 1.117] | 1.008 [0.855 - 1.187] | 1.041 [0.948 - 1.144] | 1.059 [0.969 - 1.157] | 1.057 [0.967 - 1.155] | 1.079 [1.003 - 1.16] | 1.002 [0.926 - 1.084] | 1.005 [0.896 - 1.128] | 1.026 [0.852 - 1.235] | 1.018 [0.922 - 1.123] | 1.044 [0.976 - 1.117] | 1.008 [0.855 - 1.187] |
| Xhariep | 0.933 [0.856 - 1.017] | 1.043 [0.802 - 1.355] | 1.087 [0.967 - 1.223] | 0.861 [0.762 - 0.974] | 0.891 [0.785 - 1.01] | 0.932 [0.841 - 1.032] | 0.935 [0.842 - 1.038] | 0.959 [0.821 - 1.119] | 0.901 [0.692 - 1.172] | 0.902 [0.784 - 1.039] | 0.933 [0.856 - 1.017] | 1.043 [0.802 - 1.355] |
| Zululand | 0.966 [0.908 - 1.027] | 0.844 [0.728 - 0.979] | 0.971 [0.893 - 1.057] | 0.974 [0.894 - 1.06] | 1.016 [0.936 - 1.102] | 0.97 [0.9 - 1.046] | 0.962 [0.898 - 1.031] | 0.942 [0.857 - 1.035] | 0.954 [0.819 - 1.11] | 0.961 [0.869 - 1.063] | 0.966 [0.908 - 1.027] | 0.844 [0.728 - 0.979] |

**Table S8**. Relative risks of mortality and 95% confidence intervals associated with medium-term droughts (SPEI measured at six months of accumulation, SPEI6) by district municipality in South Africa between 2009 and 2016. A higher number of positive associations and robust estimates were observed across the population of Dr. Kenneth Kaunda (Northwest), being one of the district municipalities where the highest frequency of medium-term drought conditions was recorded (Table S4).

| SPEI6 | all | <5 years | 5-24 years | 25-44 years | 45-64 years | 65-105 years | males | females | A00B99 | E00E90 | I00I99 | J00J99 |
| --- | --- | --- | --- | --- | --- | --- | --- | --- | --- | --- | --- | --- |
| Alfred Nzo | 1.005 [0.886 - 1.139] | 0.876 [0.663 - 1.157] | 0.992 [0.832 - 1.183] | 1.078 [0.913 - 1.272] | 0.969 [0.835 - 1.125] | 1.046 [0.907 - 1.206] | 1.146 [0.898 - 1.463] | 0.962 [0.612 - 1.512] | 1.256 [0.94 - 1.677] | 1.005 [0.886 - 1.139] | 0.876 [0.663 - 1.157] | 0.992 [0.832 - 1.183] |
| Amajuba | 0.961 [0.88 - 1.05] | 0.936 [0.751 - 1.166] | 1.025 [0.902 - 1.165] | 0.907 [0.796 - 1.034] | 0.935 [0.839 - 1.042] | 0.993 [0.899 - 1.098] | 0.969 [0.831 - 1.13] | 1.086 [0.869 - 1.356] | 0.928 [0.808 - 1.067] | 0.961 [0.88 - 1.05] | 0.936 [0.751 - 1.166] | 1.025 [0.902 - 1.165] |
| Amathole | 1.079 [0.996 - 1.169] | 1.312 [1.107 - 1.554] | 1.098 [0.995 - 1.213] | 1.044 [0.958 - 1.138] | 1.106 [1.01 - 1.21] | 1.055 [0.968 - 1.149] | 1.029 [0.919 - 1.151] | 0.944 [0.798 - 1.116] | 1.045 [0.941 - 1.16] | 1.079 [0.996 - 1.169] | 1.312 [1.107 - 1.554] | 1.098 [0.995 - 1.213] |
| Bojanala | 1.103 [0.996 - 1.222] | 1.024 [0.801 - 1.31] | 1.092 [0.967 - 1.234] | 1.075 [0.953 - 1.212] | 1.126 [1.002 - 1.265] | 1.081 [0.969 - 1.205] | 1.108 [0.964 - 1.273] | 1.147 [0.929 - 1.416] | 0.986 [0.869 - 1.12] | 1.103 [0.996 - 1.222] | 1.024 [0.801 - 1.31] | 1.092 [0.967 - 1.234] |
| Buffalo City | 1.064 [0.965 - 1.174] | 1.19 [0.952 - 1.488] | 1.007 [0.888 - 1.141] | 1.079 [0.956 - 1.218] | 1.091 [0.976 - 1.22] | 1.036 [0.928 - 1.158] | 1.031 [0.91 - 1.169] | 1.122 [0.868 - 1.451] | 1.096 [0.956 - 1.257] | 1.064 [0.965 - 1.174] | 1.19 [0.952 - 1.488] | 1.007 [0.888 - 1.141] |
| Cacadu | 1.116 [1.035 - 1.205] | 1.085 [0.881 - 1.336] | 1.104 [0.991 - 1.23] | 1.074 [0.967 - 1.193] | 1.149 [1.047 - 1.26] | 1.08 [0.988 - 1.182] | 1.088 [0.956 - 1.239] | 0.967 [0.777 - 1.204] | 1.097 [0.981 - 1.226] | 1.116 [1.035 - 1.205] | 1.085 [0.881 - 1.336] | 1.104 [0.991 - 1.23] |
| Cape Winelands | 1.073 [0.991 - 1.163] | 0.819 [0.636 - 1.055] | 1.114 [1.009 - 1.23] | 1.038 [0.939 - 1.147] | 1.076 [0.98 - 1.18] | 1.069 [0.971 - 1.177] | 0.996 [0.864 - 1.148] | 0.758 [0.569 - 1.011] | 1.04 [0.928 - 1.165] | 1.073 [0.991 - 1.163] | 0.819 [0.636 - 1.055] | 1.114 [1.009 - 1.23] |
| Capricorn | 1.015 [0.911 - 1.131] | 0.871 [0.679 - 1.118] | 1.029 [0.897 - 1.18] | 1.003 [0.885 - 1.136] | 1.01 [0.888 - 1.149] | 1.027 [0.915 - 1.152] | 1.015 [0.87 - 1.183] | 1.154 [0.937 - 1.42] | 1.051 [0.896 - 1.232] | 1.015 [0.911 - 1.131] | 0.871 [0.679 - 1.118] | 1.029 [0.897 - 1.18] |
| Central Karoo | 1.026 [0.892 - 1.181] | 0.943 [0.501 - 1.775] | 0.98 [0.785 - 1.223] | 1.091 [0.886 - 1.344] | 1.035 [0.862 - 1.243] | 1.012 [0.841 - 1.218] | 0.852 [0.61 - 1.191] | 1.305 [0.654 - 2.602] | 1.067 [0.833 - 1.366] | 1.026 [0.892 - 1.181] | 0.943 [0.501 - 1.775] | 0.98 [0.785 - 1.223] |
| Chris Hani | 1.038 [0.957 - 1.125] | 1.085 [0.9 - 1.306] | 1.033 [0.927 - 1.151] | 1.021 [0.929 - 1.122] | 1.028 [0.939 - 1.127] | 1.052 [0.961 - 1.15] | 1.059 [0.932 - 1.204] | 0.896 [0.748 - 1.075] | 1.059 [0.949 - 1.181] | 1.038 [0.957 - 1.125] | 1.085 [0.9 - 1.306] | 1.033 [0.927 - 1.151] |
| City of Cape Town | 0.992 [0.921 - 1.068] | 0.957 [0.825 - 1.11] | 1.011 [0.932 - 1.097] | 1.002 [0.926 - 1.083] | 1.001 [0.924 - 1.084] | 0.979 [0.906 - 1.058] | 0.952 [0.858 - 1.056] | 1.151 [0.98 - 1.351] | 1.018 [0.934 - 1.109] | 0.992 [0.921 - 1.068] | 0.957 [0.825 - 1.11] | 1.011 [0.932 - 1.097] |
| City of Johannesburg | 1.028 [0.943 - 1.12] | 1.023 [0.868 - 1.206] | 1.069 [0.973 - 1.176] | 1.006 [0.915 - 1.106] | 1.006 [0.917 - 1.103] | 1.055 [0.962 - 1.156] | 1.082 [0.969 - 1.208] | 1.128 [0.937 - 1.358] | 1.019 [0.921 - 1.128] | 1.028 [0.943 - 1.12] | 1.023 [0.868 - 1.206] | 1.069 [0.973 - 1.176] |
| City of Tshwane | 1.071 [0.97 - 1.183] | 0.998 [0.787 - 1.265] | 1.077 [0.959 - 1.211] | 1.078 [0.968 - 1.2] | 1.073 [0.964 - 1.193] | 1.069 [0.962 - 1.187] | 1.031 [0.889 - 1.196] | 1.171 [0.967 - 1.419] | 1.048 [0.933 - 1.177] | 1.071 [0.97 - 1.183] | 0.998 [0.787 - 1.265] | 1.077 [0.959 - 1.211] |
| Dr Kenneth Kaunda | 1.146 [1.04 - 1.262] | 1.284 [0.97 - 1.7] | 1.095 [0.961 - 1.247] | 1.137 [1 - 1.292] | 1.091 [0.975 - 1.221] | 1.214 [1.083 - 1.362] | 1.167 [1.005 - 1.355] | 1.174 [0.89 - 1.549] | 1.202 [1.03 - 1.403] | 1.146 [1.04 - 1.262] | 1.284 [0.97 - 1.7] | 1.095 [0.961 - 1.247] |
| Dr Ruth Segomotsi Mompati | 0.932 [0.821 - 1.058] | 1.25 [0.866 - 1.803] | 0.907 [0.761 - 1.082] | 0.96 [0.815 - 1.131] | 0.96 [0.83 - 1.11] | 0.895 [0.773 - 1.036] | 0.688 [0.563 - 0.842] | 0.988 [0.714 - 1.366] | 0.954 [0.782 - 1.164] | 0.932 [0.821 - 1.058] | 1.25 [0.866 - 1.803] | 0.907 [0.761 - 1.082] |
| Eden | 0.983 [0.892 - 1.083] | 0.93 [0.679 - 1.276] | 0.934 [0.819 - 1.065] | 0.99 [0.875 - 1.12] | 1.058 [0.942 - 1.189] | 0.898 [0.802 - 1.006] | 0.995 [0.822 - 1.205] | 0.873 [0.585 - 1.302] | 0.961 [0.83 - 1.112] | 0.983 [0.892 - 1.083] | 0.93 [0.679 - 1.276] | 0.934 [0.819 - 1.065] |
| Ehlanzeni | 1.003 [0.93 - 1.08] | 1.067 [0.908 - 1.254] | 0.999 [0.911 - 1.095] | 0.977 [0.896 - 1.066] | 1.034 [0.949 - 1.127] | 0.973 [0.898 - 1.054] | 1.03 [0.928 - 1.144] | 1.031 [0.9 - 1.182] | 0.985 [0.887 - 1.094] | 1.003 [0.93 - 1.08] | 1.067 [0.908 - 1.254] | 0.999 [0.911 - 1.095] |
| Ekurhuleni | 1.045 [0.961 - 1.137] | 0.938 [0.797 - 1.103] | 1.086 [0.985 - 1.197] | 1.035 [0.94 - 1.139] | 1.039 [0.948 - 1.139] | 1.051 [0.962 - 1.148] | 1.125 [1.003 - 1.261] | 1.08 [0.923 - 1.264] | 1.082 [0.975 - 1.2] | 1.045 [0.961 - 1.137] | 0.938 [0.797 - 1.103] | 1.086 [0.985 - 1.197] |
| eThekwini | 1.033 [0.947 - 1.127] | 1.127 [0.975 - 1.302] | 1.064 [0.965 - 1.173] | 1.016 [0.919 - 1.123] | 1.038 [0.941 - 1.145] | 1.029 [0.941 - 1.124] | 1.019 [0.912 - 1.138] | 1.087 [0.926 - 1.276] | 1.008 [0.911 - 1.115] | 1.033 [0.947 - 1.127] | 1.127 [0.975 - 1.302] | 1.064 [0.965 - 1.173] |
| Fezile Dabi | 1.087 [0.994 - 1.189] | 1.002 [0.75 - 1.337] | 1.065 [0.944 - 1.202] | 1.12 [1.001 - 1.254] | 1.087 [0.976 - 1.211] | 1.086 [0.979 - 1.206] | 0.959 [0.811 - 1.133] | 1.212 [0.968 - 1.516] | 1.095 [0.963 - 1.244] | 1.087 [0.994 - 1.189] | 1.002 [0.75 - 1.337] | 1.065 [0.944 - 1.202] |
| Frances Baard | 1.104 [0.952 - 1.281] | 0.966 [0.622 - 1.499] | 1.087 [0.891 - 1.325] | 1.257 [1.016 - 1.554] | 1.13 [0.949 - 1.345] | 1.077 [0.894 - 1.298] | 1.253 [0.979 - 1.603] | 1.542 [1.015 - 2.341] | 1.085 [0.853 - 1.381] | 1.104 [0.952 - 1.281] | 0.966 [0.622 - 1.499] | 1.087 [0.891 - 1.325] |
| Gert Sibande | 1.015 [0.927 - 1.111] | 0.896 [0.73 - 1.101] | 0.997 [0.889 - 1.118] | 1.103 [0.992 - 1.226] | 0.999 [0.903 - 1.106] | 1.032 [0.933 - 1.143] | 1.032 [0.904 - 1.177] | 1.161 [0.968 - 1.393] | 1.04 [0.913 - 1.184] | 1.015 [0.927 - 1.111] | 0.896 [0.73 - 1.101] | 0.997 [0.889 - 1.118] |
| Greater Sekhukhune | 1.06 [0.939 - 1.198] | 0.932 [0.694 - 1.251] | 1.137 [0.963 - 1.341] | 1.14 [0.992 - 1.312] | 1.067 [0.924 - 1.232] | 1.056 [0.923 - 1.208] | 1.117 [0.941 - 1.325] | 1.127 [0.873 - 1.457] | 1.044 [0.877 - 1.243] | 1.06 [0.939 - 1.198] | 0.932 [0.694 - 1.251] | 1.137 [0.963 - 1.341] |
| iLembe | 0.954 [0.854 - 1.065] | 1.07 [0.827 - 1.384] | 1.013 [0.87 - 1.179] | 0.999 [0.86 - 1.162] | 0.899 [0.79 - 1.022] | 1.015 [0.89 - 1.156] | 1.008 [0.862 - 1.178] | 0.96 [0.732 - 1.26] | 1.107 [0.94 - 1.304] | 0.954 [0.854 - 1.065] | 1.07 [0.827 - 1.384] | 1.013 [0.87 - 1.179] |
| Joe Gqabi | 1.037 [0.93 - 1.156] | 1.069 [0.79 - 1.446] | 1.068 [0.914 - 1.249] | 1.02 [0.887 - 1.173] | 1.056 [0.931 - 1.198] | 1.024 [0.9 - 1.166] | 0.998 [0.827 - 1.203] | 1.136 [0.806 - 1.601] | 0.944 [0.78 - 1.143] | 1.037 [0.93 - 1.156] | 1.069 [0.79 - 1.446] | 1.068 [0.914 - 1.249] |
| John Taolo Gaetsewe | 1.113 [0.943 - 1.315] | 1.731 [1.11 - 2.7] | 1.225 [0.981 - 1.53] | 1.049 [0.821 - 1.341] | 1.041 [0.864 - 1.253] | 1.202 [0.974 - 1.483] | 1.105 [0.821 - 1.487] | 0.878 [0.511 - 1.508] | 0.912 [0.679 - 1.223] | 1.113 [0.943 - 1.315] | 1.731 [1.11 - 2.7] | 1.225 [0.981 - 1.53] |
| Lejweleputswa | 1.072 [0.961 - 1.195] | 1.132 [0.861 - 1.487] | 1.03 [0.905 - 1.173] | 1.051 [0.92 - 1.201] | 1.037 [0.916 - 1.174] | 1.114 [0.984 - 1.262] | 0.98 [0.812 - 1.181] | 0.897 [0.704 - 1.142] | 1.048 [0.891 - 1.233] | 1.072 [0.961 - 1.195] | 1.132 [0.861 - 1.487] | 1.03 [0.905 - 1.173] |
| Mangaung | 0.979 [0.874 - 1.098] | 1.165 [0.879 - 1.544] | 0.977 [0.842 - 1.134] | 1.018 [0.888 - 1.167] | 0.954 [0.84 - 1.084] | 1.004 [0.881 - 1.144] | 1.003 [0.839 - 1.201] | 1.108 [0.858 - 1.431] | 0.926 [0.786 - 1.09] | 0.979 [0.874 - 1.098] | 1.165 [0.879 - 1.544] | 0.977 [0.842 - 1.134] |
| Mopani | 1.047 [0.967 - 1.133] | 1.148 [0.961 - 1.371] | 0.977 [0.874 - 1.092] | 1.054 [0.961 - 1.156] | 1.033 [0.94 - 1.134] | 1.062 [0.975 - 1.157] | 1.038 [0.918 - 1.174] | 1.04 [0.893 - 1.211] | 1.026 [0.907 - 1.16] | 1.047 [0.967 - 1.133] | 1.148 [0.961 - 1.371] | 0.977 [0.874 - 1.092] |
| Namakwa | 1.184 [0.976 - 1.437] | 0.871 [0.338 - 2.241] | 1.08 [0.805 - 1.449] | 1.233 [0.943 - 1.613] | 1.189 [0.925 - 1.527] | 1.176 [0.909 - 1.521] | 1.171 [0.685 - 2] | 1.167 [0.522 - 2.61] | 1.431 [1.038 - 1.974] | 1.184 [0.976 - 1.437] | 0.871 [0.338 - 2.241] | 1.08 [0.805 - 1.449] |
| Nelson Mandela Bay | 0.973 [0.848 - 1.117] | 1.206 [0.941 - 1.545] | 1.058 [0.902 - 1.24] | 0.916 [0.788 - 1.064] | 0.985 [0.852 - 1.139] | 0.96 [0.83 - 1.111] | 0.909 [0.771 - 1.073] | 0.921 [0.695 - 1.22] | 0.981 [0.836 - 1.151] | 0.973 [0.848 - 1.117] | 1.206 [0.941 - 1.545] | 1.058 [0.902 - 1.24] |
| Ngaka Modiri Molema | 1.039 [0.946 - 1.141] | 0.94 [0.738 - 1.197] | 1.04 [0.926 - 1.168] | 1.067 [0.949 - 1.2] | 1.005 [0.901 - 1.121] | 1.084 [0.979 - 1.201] | 0.996 [0.861 - 1.152] | 1.025 [0.824 - 1.275] | 0.969 [0.849 - 1.106] | 1.039 [0.946 - 1.141] | 0.94 [0.738 - 1.197] | 1.04 [0.926 - 1.168] |
| Nkangala | 1.091 [0.984 - 1.211] | 1.269 [0.961 - 1.676] | 1.056 [0.926 - 1.205] | 1.107 [0.977 - 1.255] | 1.088 [0.962 - 1.23] | 1.092 [0.975 - 1.223] | 1.151 [0.977 - 1.356] | 1.002 [0.801 - 1.254] | 1.053 [0.906 - 1.223] | 1.091 [0.984 - 1.211] | 1.269 [0.961 - 1.676] | 1.056 [0.926 - 1.205] |
| O.R.Tambo | 0.989 [0.904 - 1.082] | 0.974 [0.826 - 1.148] | 1.021 [0.913 - 1.143] | 0.977 [0.881 - 1.084] | 0.998 [0.903 - 1.104] | 0.978 [0.889 - 1.077] | 0.962 [0.848 - 1.09] | 1.03 [0.817 - 1.3] | 0.972 [0.836 - 1.129] | 0.989 [0.904 - 1.082] | 0.974 [0.826 - 1.148] | 1.021 [0.913 - 1.143] |
| Overberg | 0.98 [0.851 - 1.128] | 0.651 [0.384 - 1.102] | 0.939 [0.767 - 1.151] | 1.029 [0.855 - 1.24] | 0.899 [0.752 - 1.073] | 1.09 [0.909 - 1.307] | 1.045 [0.749 - 1.458] | 0.892 [0.51 - 1.561] | 0.953 [0.768 - 1.184] | 0.98 [0.851 - 1.128] | 0.651 [0.384 - 1.102] | 0.939 [0.767 - 1.151] |
| Pixley ka Seme | 0.975 [0.893 - 1.065] | 0.89 [0.682 - 1.162] | 0.969 [0.858 - 1.095] | 0.995 [0.881 - 1.123] | 0.924 [0.836 - 1.02] | 1.032 [0.926 - 1.15] | 0.944 [0.81 - 1.1] | 0.986 [0.759 - 1.28] | 0.969 [0.85 - 1.105] | 0.975 [0.893 - 1.065] | 0.89 [0.682 - 1.162] | 0.969 [0.858 - 1.095] |
| Sedibeng | 1.072 [0.984 - 1.167] | 1.009 [0.823 - 1.237] | 1.069 [0.962 - 1.187] | 1.127 [1.014 - 1.253] | 1.059 [0.962 - 1.166] | 1.088 [0.987 - 1.2] | 1.18 [1.033 - 1.346] | 1.059 [0.854 - 1.313] | 1.201 [1.07 - 1.349] | 1.072 [0.984 - 1.167] | 1.009 [0.823 - 1.237] | 1.069 [0.962 - 1.187] |
| Sisonke | 1.1 [0.976 - 1.24] | 1.134 [0.867 - 1.482] | 0.999 [0.845 - 1.182] | 1.275 [1.084 - 1.498] | 1.16 [1.011 - 1.33] | 1.049 [0.908 - 1.211] | 1.005 [0.834 - 1.21] | 1.154 [0.855 - 1.557] | 1.169 [0.957 - 1.427] | 1.1 [0.976 - 1.24] | 1.134 [0.867 - 1.482] | 0.999 [0.845 - 1.182] |
| Siyanda | 1.029 [0.907 - 1.166] | 0.856 [0.567 - 1.292] | 1.028 [0.859 - 1.23] | 1.104 [0.922 - 1.321] | 1.008 [0.869 - 1.169] | 1.048 [0.897 - 1.224] | 0.961 [0.759 - 1.216] | 1.184 [0.763 - 1.836] | 1.015 [0.823 - 1.25] | 1.029 [0.907 - 1.166] | 0.856 [0.567 - 1.292] | 1.028 [0.859 - 1.23] |
| Thabo Mofutsanyane | 1.066 [0.975 - 1.165] | 0.954 [0.774 - 1.176] | 1.14 [1.021 - 1.272] | 1.117 [0.996 - 1.253] | 1.048 [0.944 - 1.163] | 1.085 [0.986 - 1.195] | 0.93 [0.813 - 1.064] | 1.195 [0.986 - 1.449] | 1.13 [0.998 - 1.279] | 1.066 [0.975 - 1.165] | 0.954 [0.774 - 1.176] | 1.14 [1.021 - 1.272] |
| Ugu | 1.049 [0.931 - 1.183] | 1.066 [0.832 - 1.366] | 1.143 [0.97 - 1.347] | 1.082 [0.932 - 1.258] | 1.099 [0.956 - 1.263] | 0.998 [0.877 - 1.136] | 0.993 [0.843 - 1.171] | 0.832 [0.629 - 1.1] | 1.199 [1.011 - 1.421] | 1.049 [0.931 - 1.183] | 1.066 [0.832 - 1.366] | 1.143 [0.97 - 1.347] |
| UMgungundlovu | 1.006 [0.908 - 1.114] | 1.033 [0.817 - 1.306] | 1.064 [0.937 - 1.207] | 0.988 [0.866 - 1.126] | 1.004 [0.889 - 1.135] | 1.011 [0.906 - 1.129] | 0.97 [0.827 - 1.137] | 1.162 [0.922 - 1.464] | 0.951 [0.833 - 1.087] | 1.006 [0.908 - 1.114] | 1.033 [0.817 - 1.306] | 1.064 [0.937 - 1.207] |
| Umkhanyakude | 0.905 [0.781 - 1.049] | 0.976 [0.664 - 1.433] | 0.991 [0.794 - 1.236] | 0.807 [0.66 - 0.986] | 0.93 [0.781 - 1.107] | 0.888 [0.747 - 1.057] | 0.89 [0.717 - 1.106] | 0.911 [0.598 - 1.388] | 0.795 [0.623 - 1.016] | 0.905 [0.781 - 1.049] | 0.976 [0.664 - 1.433] | 0.991 [0.794 - 1.236] |
| Umzinyathi | 1.065 [0.967 - 1.174] | 1.088 [0.855 - 1.383] | 1.135 [0.981 - 1.312] | 1.042 [0.912 - 1.19] | 1.121 [0.998 - 1.259] | 1.017 [0.91 - 1.137] | 1.012 [0.862 - 1.188] | 1.022 [0.795 - 1.314] | 1.105 [0.943 - 1.293] | 1.065 [0.967 - 1.174] | 1.088 [0.855 - 1.383] | 1.135 [0.981 - 1.312] |
| Uthukela | 0.985 [0.903 - 1.074] | 0.912 [0.739 - 1.126] | 0.916 [0.809 - 1.037] | 0.977 [0.874 - 1.092] | 1.006 [0.909 - 1.114] | 0.96 [0.868 - 1.062] | 0.926 [0.82 - 1.046] | 1.047 [0.851 - 1.289] | 0.947 [0.83 - 1.081] | 0.985 [0.903 - 1.074] | 0.912 [0.739 - 1.126] | 0.916 [0.809 - 1.037] |
| Uthungulu | 0.984 [0.89 - 1.088] | 0.982 [0.771 - 1.251] | 0.998 [0.874 - 1.139] | 0.991 [0.873 - 1.124] | 1.004 [0.89 - 1.131] | 0.965 [0.861 - 1.08] | 1.063 [0.912 - 1.239] | 0.939 [0.724 - 1.218] | 1.021 [0.87 - 1.199] | 0.984 [0.89 - 1.088] | 0.982 [0.771 - 1.251] | 0.998 [0.874 - 1.139] |
| Vhembe | 0.965 [0.855 - 1.089] | 0.849 [0.63 - 1.144] | 1.036 [0.881 - 1.218] | 0.991 [0.859 - 1.144] | 0.985 [0.853 - 1.138] | 0.945 [0.826 - 1.082] | 0.859 [0.695 - 1.062] | 0.853 [0.662 - 1.099] | 1.033 [0.845 - 1.264] | 0.965 [0.855 - 1.089] | 0.849 [0.63 - 1.144] | 1.036 [0.881 - 1.218] |
| Waterberg | 1.039 [0.913 - 1.181] | 1.016 [0.704 - 1.468] | 1.172 [0.975 - 1.407] | 1.011 [0.858 - 1.191] | 1.125 [0.966 - 1.31] | 0.949 [0.818 - 1.102] | 1.036 [0.836 - 1.284] | 0.998 [0.727 - 1.371] | 0.942 [0.776 - 1.143] | 1.039 [0.913 - 1.181] | 1.016 [0.704 - 1.468] | 1.172 [0.975 - 1.407] |
| West Coast | 0.991 [0.897 - 1.095] | 1.274 [0.923 - 1.757] | 0.974 [0.843 - 1.126] | 0.934 [0.826 - 1.055] | 0.981 [0.872 - 1.104] | 1.001 [0.886 - 1.132] | 1.036 [0.848 - 1.265] | 1.255 [0.867 - 1.817] | 0.977 [0.844 - 1.131] | 0.991 [0.897 - 1.095] | 1.274 [0.923 - 1.757] | 0.974 [0.843 - 1.126] |
| West Rand | 1.091 [0.989 - 1.202] | 1.116 [0.881 - 1.414] | 1.164 [1.024 - 1.324] | 1.066 [0.938 - 1.212] | 1.054 [0.947 - 1.173] | 1.133 [1.011 - 1.269] | 1.089 [0.921 - 1.287] | 1.078 [0.825 - 1.408] | 1.074 [0.932 - 1.239] | 1.091 [0.989 - 1.202] | 1.116 [0.881 - 1.414] | 1.164 [1.024 - 1.324] |
| Xhariep | 1.089 [0.939 - 1.264] | 0.956 [0.597 - 1.533] | 1.095 [0.891 - 1.345] | 1.145 [0.928 - 1.413] | 1.015 [0.851 - 1.21] | 1.179 [0.984 - 1.413] | 0.765 [0.584 - 1.002] | 1.526 [0.982 - 2.372] | 1.21 [0.959 - 1.528] | 1.089 [0.939 - 1.264] | 0.956 [0.597 - 1.533] | 1.095 [0.891 - 1.345] |
| Zululand | 0.968 [0.864 - 1.086] | 0.795 [0.609 - 1.037] | 1.083 [0.925 - 1.268] | 1.068 [0.916 - 1.245] | 1.02 [0.888 - 1.173] | 0.925 [0.814 - 1.051] | 0.921 [0.775 - 1.093] | 0.914 [0.685 - 1.22] | 0.95 [0.786 - 1.148] | 0.968 [0.864 - 1.086] | 0.795 [0.609 - 1.037] | 1.083 [0.925 - 1.268] |

**Table S9**. Relative risks of mortality and 95% confidence intervals associated with long-term droughts (SPEI measured at twelve months of accumulation, SPEI12) by district municipality in South Africa between 2009 and 2016. Higher imprecision was observed in the estimates for long-term droughts in many district municipalities for the total population compared to other types of droughts. Buffalo City (Eastern Cape) and Umkhanyakude (Kwazulu-Natal) were the most affected locations in terms of death, but the frequency and severity of long-term droughts in these regions were not high (Table S4).

| SPEI12 | all | <5 years | 5-24 years | 25-44 years | 45-64 years | 65-105 years | males | females | A00B99 | E00E90 | I00I99 | J00J99 |
| --- | --- | --- | --- | --- | --- | --- | --- | --- | --- | --- | --- | --- |
| Alfred Nzo | 1.123 [0.929 - 1.356] | 1.245 [0.826 - 1.877] | 1.156 [0.89 - 1.502] | 1.175 [0.908 - 1.52] | 1.088 [0.868 - 1.362] | 1.163 [0.938 - 1.441] | 1.283 [0.897 - 1.833] | 1.201 [0.612 - 2.356] | 1.141 [0.735 - 1.771] | 1.123 [0.929 - 1.356] | 1.245 [0.826 - 1.877] | 1.156 [0.89 - 1.502] |
| Amajuba | 0.976 [0.861 - 1.107] | 1.221 [0.893 - 1.669] | 1.028 [0.857 - 1.234] | 0.974 [0.812 - 1.167] | 0.97 [0.832 - 1.132] | 0.994 [0.863 - 1.146] | 0.874 [0.701 - 1.088] | 1.091 [0.801 - 1.487] | 1.12 [0.923 - 1.359] | 0.976 [0.861 - 1.107] | 1.221 [0.893 - 1.669] | 1.028 [0.857 - 1.234] |
| Amathole | 1.088 [0.959 - 1.234] | 1.109 [0.846 - 1.454] | 1.207 [1.033 - 1.411] | 1.025 [0.893 - 1.176] | 1.129 [0.979 - 1.302] | 1.047 [0.916 - 1.198] | 1.202 [1.015 - 1.424] | 1.239 [0.949 - 1.618] | 1.191 [1.009 - 1.405] | 1.088 [0.959 - 1.234] | 1.109 [0.846 - 1.454] | 1.207 [1.033 - 1.411] |
| Bojanala | 1.106 [0.964 - 1.269] | 0.947 [0.684 - 1.311] | 1.107 [0.94 - 1.303] | 1.141 [0.97 - 1.342] | 1.128 [0.965 - 1.319] | 1.087 [0.939 - 1.258] | 1.051 [0.872 - 1.267] | 1.136 [0.857 - 1.507] | 1.103 [0.93 - 1.308] | 1.106 [0.964 - 1.269] | 0.947 [0.684 - 1.311] | 1.107 [0.94 - 1.303] |
| Buffalo City | 1.272 [1.125 - 1.439] | 1.305 [0.99 - 1.719] | 1.228 [1.047 - 1.441] | 1.399 [1.198 - 1.634] | 1.337 [1.161 - 1.539] | 1.21 [1.052 - 1.39] | 1.043 [0.893 - 1.218] | 1.45 [1.038 - 2.025] | 1.464 [1.231 - 1.74] | 1.272 [1.125 - 1.439] | 1.305 [0.99 - 1.719] | 1.228 [1.047 - 1.441] |
| Cacadu | 1.052 [0.939 - 1.177] | 1.079 [0.807 - 1.444] | 1.05 [0.893 - 1.235] | 0.946 [0.806 - 1.11] | 1.053 [0.917 - 1.21] | 1.051 [0.922 - 1.198] | 1.057 [0.879 - 1.272] | 0.991 [0.713 - 1.378] | 1.094 [0.923 - 1.295] | 1.052 [0.939 - 1.177] | 1.079 [0.807 - 1.444] | 1.05 [0.893 - 1.235] |
| Cape Winelands | 1.032 [0.914 - 1.164] | 0.742 [0.505 - 1.09] | 1.017 [0.874 - 1.183] | 1.072 [0.924 - 1.243] | 1.039 [0.903 - 1.196] | 1.028 [0.889 - 1.189] | 1.105 [0.895 - 1.366] | 0.706 [0.462 - 1.081] | 1.089 [0.92 - 1.289] | 1.032 [0.914 - 1.164] | 0.742 [0.505 - 1.09] | 1.017 [0.874 - 1.183] |
| Capricorn | 1.156 [0.94 - 1.422] | 0.693 [0.427 - 1.123] | 1.151 [0.884 - 1.499] | 1.201 [0.95 - 1.517] | 1.121 [0.874 - 1.438] | 1.201 [0.964 - 1.496] | 1.173 [0.868 - 1.585] | 1.428 [0.97 - 2.101] | 1.164 [0.862 - 1.574] | 1.156 [0.94 - 1.422] | 0.693 [0.427 - 1.123] | 1.151 [0.884 - 1.499] |
| Central Karoo | 1.041 [0.872 - 1.241] | 0.893 [0.422 - 1.89] | 0.948 [0.716 - 1.256] | 1.042 [0.802 - 1.355] | 1.106 [0.878 - 1.392] | 0.968 [0.768 - 1.22] | 0.948 [0.632 - 1.421] | 1.012 [0.399 - 2.57] | 1.155 [0.848 - 1.573] | 1.041 [0.872 - 1.241] | 0.893 [0.422 - 1.89] | 0.948 [0.716 - 1.256] |
| Chris Hani | 0.896 [0.751 - 1.069] | 0.841 [0.56 - 1.264] | 0.846 [0.668 - 1.072] | 1.054 [0.857 - 1.295] | 0.821 [0.673 - 1.003] | 0.992 [0.817 - 1.206] | 0.805 [0.61 - 1.061] | 0.728 [0.494 - 1.073] | 0.886 [0.696 - 1.127] | 0.896 [0.751 - 1.069] | 0.841 [0.56 - 1.264] | 0.846 [0.668 - 1.072] |
| City of Cape Town | 0.99 [0.875 - 1.12] | 0.939 [0.733 - 1.203] | 1.012 [0.884 - 1.158] | 0.994 [0.873 - 1.131] | 0.985 [0.862 - 1.124] | 1 [0.88 - 1.137] | 0.931 [0.783 - 1.106] | 0.973 [0.743 - 1.275] | 0.998 [0.866 - 1.15] | 0.99 [0.875 - 1.12] | 0.939 [0.733 - 1.203] | 1.012 [0.884 - 1.158] |
| City of Johannesburg | 1.037 [0.929 - 1.156] | 1.06 [0.861 - 1.305] | 1.057 [0.937 - 1.192] | 1.025 [0.908 - 1.157] | 1.023 [0.91 - 1.15] | 1.047 [0.932 - 1.176] | 1.103 [0.959 - 1.268] | 0.905 [0.715 - 1.145] | 1.033 [0.907 - 1.175] | 1.037 [0.929 - 1.156] | 1.06 [0.861 - 1.305] | 1.057 [0.937 - 1.192] |
| City of Tshwane | 1.15 [1.006 - 1.316] | 1.335 [0.968 - 1.841] | 1.166 [0.995 - 1.367] | 1.078 [0.93 - 1.249] | 1.189 [1.029 - 1.375] | 1.106 [0.958 - 1.277] | 1.248 [1.022 - 1.525] | 1.254 [0.963 - 1.633] | 1.027 [0.876 - 1.204] | 1.15 [1.006 - 1.316] | 1.335 [0.968 - 1.841] | 1.166 [0.995 - 1.367] |
| Dr Kenneth Kaunda | 1.002 [0.869 - 1.156] | 0.991 [0.656 - 1.498] | 1.034 [0.855 - 1.251] | 0.991 [0.821 - 1.197] | 0.963 [0.816 - 1.135] | 1.057 [0.893 - 1.253] | 1.131 [0.909 - 1.407] | 0.866 [0.583 - 1.287] | 0.943 [0.752 - 1.182] | 1.002 [0.869 - 1.156] | 0.991 [0.656 - 1.498] | 1.034 [0.855 - 1.251] |
| Dr Ruth Segomotsi Mompati | 0.927 [0.778 - 1.105] | 1.321 [0.806 - 2.166] | 0.78 [0.611 - 0.996] | 1.084 [0.864 - 1.359] | 0.968 [0.792 - 1.184] | 0.881 [0.72 - 1.078] | 0.658 [0.496 - 0.874] | 0.898 [0.593 - 1.358] | 0.965 [0.732 - 1.271] | 0.927 [0.778 - 1.105] | 1.321 [0.806 - 2.166] | 0.78 [0.611 - 0.996] |
| Eden | 0.907 [0.796 - 1.034] | 0.902 [0.597 - 1.361] | 0.852 [0.714 - 1.016] | 0.967 [0.817 - 1.144] | 0.913 [0.781 - 1.069] | 0.905 [0.777 - 1.055] | 0.776 [0.597 - 1.008] | 0.923 [0.537 - 1.586] | 0.916 [0.751 - 1.116] | 0.907 [0.796 - 1.034] | 0.902 [0.597 - 1.361] | 0.852 [0.714 - 1.016] |
| Ehlanzeni | 1.064 [0.938 - 1.207] | 1.264 [0.964 - 1.659] | 1.075 [0.92 - 1.256] | 1.072 [0.927 - 1.241] | 1.093 [0.945 - 1.265] | 1.032 [0.902 - 1.18] | 1.074 [0.898 - 1.283] | 1.099 [0.872 - 1.384] | 1.083 [0.911 - 1.288] | 1.064 [0.938 - 1.207] | 1.264 [0.964 - 1.659] | 1.075 [0.92 - 1.256] |
| Ekurhuleni | 1.096 [0.983 - 1.222] | 1.056 [0.858 - 1.299] | 1.065 [0.939 - 1.208] | 1.124 [0.993 - 1.272] | 1.097 [0.974 - 1.235] | 1.094 [0.976 - 1.226] | 1.115 [0.961 - 1.292] | 0.967 [0.791 - 1.183] | 1.115 [0.975 - 1.275] | 1.096 [0.983 - 1.222] | 1.056 [0.858 - 1.299] | 1.065 [0.939 - 1.208] |
| eThekwini | 0.985 [0.854 - 1.136] | 0.879 [0.686 - 1.126] | 1.05 [0.894 - 1.233] | 0.953 [0.815 - 1.115] | 1.013 [0.863 - 1.19] | 0.953 [0.823 - 1.104] | 1.026 [0.851 - 1.237] | 0.922 [0.701 - 1.212] | 0.941 [0.801 - 1.105] | 0.985 [0.854 - 1.136] | 0.879 [0.686 - 1.126] | 1.05 [0.894 - 1.233] |
| Fezile Dabi | 0.969 [0.839 - 1.12] | 0.715 [0.446 - 1.147] | 0.981 [0.808 - 1.19] | 1.017 [0.847 - 1.22] | 0.991 [0.831 - 1.181] | 0.949 [0.803 - 1.123] | 0.981 [0.753 - 1.279] | 1.028 [0.72 - 1.466] | 0.896 [0.73 - 1.1] | 0.969 [0.839 - 1.12] | 0.715 [0.446 - 1.147] | 0.981 [0.808 - 1.19] |
| Frances Baard | 1.103 [0.864 - 1.41] | 0.566 [0.276 - 1.158] | 1.334 [0.961 - 1.852] | 1.018 [0.719 - 1.442] | 1.175 [0.881 - 1.568] | 1.038 [0.764 - 1.411] | 1.191 [0.799 - 1.776] | 2.018 [1.001 - 4.069] | 1.015 [0.68 - 1.517] | 1.103 [0.864 - 1.41] | 0.566 [0.276 - 1.158] | 1.334 [0.961 - 1.852] |
| Gert Sibande | 1.001 [0.878 - 1.14] | 0.976 [0.729 - 1.306] | 0.936 [0.793 - 1.103] | 1.105 [0.947 - 1.29] | 0.951 [0.822 - 1.101] | 1.057 [0.914 - 1.223] | 0.946 [0.784 - 1.142] | 1.215 [0.937 - 1.577] | 1.151 [0.953 - 1.391] | 1.001 [0.878 - 1.14] | 0.976 [0.729 - 1.306] | 0.936 [0.793 - 1.103] |
| Greater Sekhukhune | 1.063 [0.869 - 1.3] | 1.002 [0.611 - 1.642] | 1.155 [0.88 - 1.515] | 1.103 [0.878 - 1.387] | 1.144 [0.903 - 1.448] | 0.989 [0.79 - 1.237] | 1.178 [0.874 - 1.586] | 1.043 [0.689 - 1.579] | 0.887 [0.671 - 1.173] | 1.063 [0.869 - 1.3] | 1.002 [0.611 - 1.642] | 1.155 [0.88 - 1.515] |
| iLembe | 0.977 [0.825 - 1.159] | 1.273 [0.843 - 1.924] | 1.052 [0.833 - 1.329] | 1.106 [0.886 - 1.382] | 0.911 [0.746 - 1.111] | 1.055 [0.862 - 1.292] | 0.938 [0.73 - 1.206] | 0.867 [0.583 - 1.288] | 1.087 [0.847 - 1.395] | 0.977 [0.825 - 1.159] | 1.273 [0.843 - 1.924] | 1.052 [0.833 - 1.329] |
| Joe Gqabi | 1.059 [0.863 - 1.298] | 0.763 [0.437 - 1.331] | 1.284 [0.959 - 1.719] | 1.017 [0.78 - 1.325] | 1.033 [0.814 - 1.31] | 1.081 [0.848 - 1.377] | 0.894 [0.628 - 1.273] | 1.122 [0.59 - 2.136] | 1.082 [0.751 - 1.557] | 1.059 [0.863 - 1.298] | 0.763 [0.437 - 1.331] | 1.284 [0.959 - 1.719] |
| John Taolo Gaetsewe | 1.129 [0.857 - 1.489] | 1.775 [0.854 - 3.69] | 1.198 [0.827 - 1.737] | 1.239 [0.815 - 1.882] | 1.104 [0.808 - 1.507] | 1.16 [0.821 - 1.641] | 1.262 [0.79 - 2.014] | 0.607 [0.245 - 1.502] | 1.079 [0.647 - 1.797] | 1.129 [0.857 - 1.489] | 1.775 [0.854 - 3.69] | 1.198 [0.827 - 1.737] |
| Lejweleputswa | 1.046 [0.875 - 1.251] | 0.944 [0.597 - 1.494] | 1.005 [0.812 - 1.243] | 1.014 [0.817 - 1.259] | 1.051 [0.857 - 1.288] | 1.035 [0.842 - 1.271] | 0.886 [0.651 - 1.206] | 1.045 [0.708 - 1.543] | 1.117 [0.858 - 1.454] | 1.046 [0.875 - 1.251] | 0.944 [0.597 - 1.494] | 1.005 [0.812 - 1.243] |
| Mangaung | 0.916 [0.751 - 1.116] | 1.334 [0.814 - 2.184] | 0.942 [0.728 - 1.22] | 0.928 [0.73 - 1.18] | 0.876 [0.702 - 1.093] | 0.963 [0.767 - 1.208] | 0.793 [0.585 - 1.077] | 0.761 [0.491 - 1.18] | 0.954 [0.715 - 1.274] | 0.916 [0.751 - 1.116] | 1.334 [0.814 - 2.184] | 0.942 [0.728 - 1.22] |
| Mopani | 0.951 [0.839 - 1.078] | 1.145 [0.856 - 1.532] | 0.953 [0.799 - 1.137] | 0.984 [0.853 - 1.135] | 0.942 [0.813 - 1.092] | 0.966 [0.843 - 1.107] | 0.935 [0.77 - 1.134] | 1.104 [0.872 - 1.397] | 1.011 [0.839 - 1.219] | 0.951 [0.839 - 1.078] | 1.145 [0.856 - 1.532] | 0.953 [0.799 - 1.137] |
| Namakwa | 1.441 [1.109 - 1.873] | 2.101 [0.636 - 6.941] | 1.094 [0.733 - 1.631] | 1.46 [1.01 - 2.11] | 1.57 [1.114 - 2.213] | 1.313 [0.927 - 1.861] | 1.171 [0.555 - 2.472] | 1.174 [0.377 - 3.659] | 1.561 [0.991 - 2.46] | 1.441 [1.109 - 1.873] | 2.101 [0.636 - 6.941] | 1.094 [0.733 - 1.631] |
| Nelson Mandela Bay | 0.87 [0.765 - 0.988] | 0.748 [0.593 - 0.943] | 0.877 [0.756 - 1.018] | 0.918 [0.798 - 1.056] | 0.895 [0.781 - 1.025] | 0.844 [0.738 - 0.965] | 0.875 [0.752 - 1.019] | 0.959 [0.734 - 1.252] | 0.977 [0.841 - 1.136] | 0.87 [0.765 - 0.988] | 0.748 [0.593 - 0.943] | 0.877 [0.756 - 1.018] |
| Ngaka Modiri Molema | 1.086 [0.951 - 1.242] | 0.878 [0.621 - 1.242] | 1.122 [0.95 - 1.324] | 1.087 [0.919 - 1.285] | 1.072 [0.918 - 1.252] | 1.101 [0.951 - 1.274] | 0.957 [0.78 - 1.175] | 0.917 [0.675 - 1.246] | 1.154 [0.956 - 1.393] | 1.086 [0.951 - 1.242] | 0.878 [0.621 - 1.242] | 1.122 [0.95 - 1.324] |
| Nkangala | 1.11 [0.963 - 1.279] | 1.551 [1.065 - 2.258] | 1.086 [0.907 - 1.3] | 1.143 [0.962 - 1.357] | 1.14 [0.964 - 1.348] | 1.077 [0.922 - 1.258] | 1.119 [0.896 - 1.398] | 0.959 [0.707 - 1.301] | 1.164 [0.946 - 1.431] | 1.11 [0.963 - 1.279] | 1.551 [1.065 - 2.258] | 1.086 [0.907 - 1.3] |
| O.R.Tambo | 0.983 [0.84 - 1.15] | 0.97 [0.728 - 1.292] | 0.936 [0.769 - 1.139] | 1.019 [0.848 - 1.224] | 0.991 [0.83 - 1.183] | 0.975 [0.825 - 1.153] | 0.979 [0.788 - 1.218] | 0.959 [0.637 - 1.444] | 1.227 [0.945 - 1.593] | 0.983 [0.84 - 1.15] | 0.97 [0.728 - 1.292] | 0.936 [0.769 - 1.139] |
| Overberg | 1.105 [0.951 - 1.283] | 0.91 [0.519 - 1.594] | 1.012 [0.817 - 1.255] | 1.148 [0.941 - 1.4] | 1.078 [0.894 - 1.3] | 1.141 [0.937 - 1.39] | 0.853 [0.596 - 1.223] | 0.942 [0.513 - 1.731] | 1.099 [0.874 - 1.382] | 1.105 [0.951 - 1.283] | 0.91 [0.519 - 1.594] | 1.012 [0.817 - 1.255] |
| Pixley ka Seme | 1.115 [0.931 - 1.335] | 1.2 [0.679 - 2.119] | 1.099 [0.857 - 1.409] | 1.162 [0.914 - 1.478] | 1.11 [0.906 - 1.361] | 1.129 [0.904 - 1.41] | 1.057 [0.755 - 1.479] | 1.217 [0.723 - 2.049] | 1.009 [0.778 - 1.31] | 1.115 [0.931 - 1.335] | 1.2 [0.679 - 2.119] | 1.099 [0.857 - 1.409] |
| Sedibeng | 0.998 [0.885 - 1.126] | 0.921 [0.692 - 1.226] | 0.96 [0.828 - 1.113] | 1.044 [0.899 - 1.213] | 0.963 [0.841 - 1.104] | 1.043 [0.908 - 1.198] | 0.92 [0.763 - 1.109] | 1.059 [0.785 - 1.429] | 1.143 [0.969 - 1.347] | 0.998 [0.885 - 1.126] | 0.921 [0.692 - 1.226] | 0.96 [0.828 - 1.113] |
| Sisonke | 1.15 [0.949 - 1.395] | 1.105 [0.722 - 1.691] | 1.024 [0.783 - 1.339] | 1.481 [1.148 - 1.912] | 1.237 [0.992 - 1.541] | 1.079 [0.856 - 1.36] | 1.11 [0.817 - 1.508] | 1.245 [0.772 - 2.009] | 1.025 [0.751 - 1.4] | 1.15 [0.949 - 1.395] | 1.105 [0.722 - 1.691] | 1.024 [0.783 - 1.339] |
| Siyanda | 0.902 [0.698 - 1.165] | 0.629 [0.265 - 1.492] | 0.861 [0.598 - 1.239] | 0.969 [0.674 - 1.393] | 0.888 [0.655 - 1.204] | 0.918 [0.67 - 1.259] | 0.82 [0.5 - 1.342] | 0.881 [0.362 - 2.143] | 0.826 [0.548 - 1.247] | 0.902 [0.698 - 1.165] | 0.629 [0.265 - 1.492] | 0.861 [0.598 - 1.239] |
| Thabo Mofutsanyane | 1.042 [0.907 - 1.198] | 1.16 [0.838 - 1.605] | 1.085 [0.914 - 1.29] | 1.102 [0.924 - 1.314] | 0.986 [0.839 - 1.159] | 1.102 [0.948 - 1.281] | 0.887 [0.718 - 1.095] | 1.028 [0.765 - 1.38] | 1.161 [0.959 - 1.407] | 1.042 [0.907 - 1.198] | 1.16 [0.838 - 1.605] | 1.085 [0.914 - 1.29] |
| Ugu | 1.073 [0.875 - 1.317] | 0.776 [0.505 - 1.191] | 1.072 [0.81 - 1.418] | 1.133 [0.881 - 1.456] | 1.102 [0.869 - 1.398] | 1.047 [0.84 - 1.306] | 1.128 [0.852 - 1.495] | 1.536 [0.961 - 2.457] | 0.953 [0.715 - 1.269] | 1.073 [0.875 - 1.317] | 0.776 [0.505 - 1.191] | 1.072 [0.81 - 1.418] |
| UMgungundlovu | 0.971 [0.827 - 1.139] | 1.007 [0.692 - 1.465] | 1.008 [0.828 - 1.228] | 0.995 [0.816 - 1.213] | 0.961 [0.794 - 1.164] | 0.978 [0.824 - 1.162] | 0.912 [0.706 - 1.179] | 1.104 [0.766 - 1.589] | 0.983 [0.803 - 1.204] | 0.971 [0.827 - 1.139] | 1.007 [0.692 - 1.465] | 1.008 [0.828 - 1.228] |
| Umkhanyakude | 1.32 [1.042 - 1.672] | 1.911 [1.051 - 3.476] | 1.523 [1.062 - 2.183] | 1.264 [0.918 - 1.739] | 1.348 [1.017 - 1.787] | 1.29 [0.978 - 1.702] | 1.356 [0.958 - 1.921] | 0.989 [0.494 - 1.977] | 1.503 [1.016 - 2.226] | 1.32 [1.042 - 1.672] | 1.911 [1.051 - 3.476] | 1.523 [1.062 - 2.183] |
| Umzinyathi | 1.05 [0.92 - 1.199] | 0.897 [0.642 - 1.254] | 1.139 [0.933 - 1.389] | 1.1 [0.925 - 1.307] | 1.081 [0.922 - 1.267] | 1.02 [0.877 - 1.187] | 1.057 [0.845 - 1.322] | 0.887 [0.628 - 1.252] | 1.005 [0.818 - 1.235] | 1.05 [0.92 - 1.199] | 0.897 [0.642 - 1.254] | 1.139 [0.933 - 1.389] |
| Uthukela | 1.06 [0.932 - 1.205] | 1.062 [0.782 - 1.442] | 1.047 [0.871 - 1.259] | 1.039 [0.881 - 1.225] | 1.043 [0.897 - 1.213] | 1.078 [0.929 - 1.25] | 0.982 [0.819 - 1.177] | 0.968 [0.708 - 1.323] | 1 [0.821 - 1.218] | 1.06 [0.932 - 1.205] | 1.062 [0.782 - 1.442] | 1.047 [0.871 - 1.259] |
| Uthungulu | 0.907 [0.779 - 1.055] | 1.08 [0.741 - 1.575] | 0.95 [0.779 - 1.158] | 1.018 [0.846 - 1.226] | 0.877 [0.732 - 1.051] | 0.948 [0.799 - 1.124] | 0.891 [0.7 - 1.135] | 0.642 [0.444 - 0.929] | 0.945 [0.738 - 1.211] | 0.907 [0.779 - 1.055] | 1.08 [0.741 - 1.575] | 0.95 [0.779 - 1.158] |
| Vhembe | 0.91 [0.783 - 1.057] | 0.87 [0.6 - 1.261] | 0.973 [0.794 - 1.191] | 0.969 [0.816 - 1.152] | 0.91 [0.76 - 1.089] | 0.905 [0.766 - 1.069] | 0.833 [0.633 - 1.097] | 0.765 [0.567 - 1.034] | 0.845 [0.657 - 1.086] | 0.91 [0.783 - 1.057] | 0.87 [0.6 - 1.261] | 0.973 [0.794 - 1.191] |
| Waterberg | 0.977 [0.799 - 1.194] | 0.964 [0.539 - 1.724] | 1.048 [0.788 - 1.395] | 0.963 [0.748 - 1.238] | 1.109 [0.873 - 1.408] | 0.857 [0.681 - 1.08] | 0.775 [0.559 - 1.073] | 0.766 [0.477 - 1.231] | 0.861 [0.642 - 1.156] | 0.977 [0.799 - 1.194] | 0.964 [0.539 - 1.724] | 1.048 [0.788 - 1.395] |
| West Coast | 0.988 [0.821 - 1.188] | 1.396 [0.743 - 2.621] | 0.917 [0.702 - 1.198] | 0.93 [0.744 - 1.162] | 0.947 [0.761 - 1.178] | 1.039 [0.828 - 1.304] | 1.507 [1.043 - 2.177] | 1.132 [0.571 - 2.243] | 0.931 [0.709 - 1.221] | 0.988 [0.821 - 1.188] | 1.396 [0.743 - 2.621] | 0.917 [0.702 - 1.198] |
| West Rand | 1.033 [0.919 - 1.162] | 0.946 [0.715 - 1.252] | 1.106 [0.947 - 1.29] | 0.988 [0.846 - 1.155] | 0.974 [0.857 - 1.107] | 1.113 [0.971 - 1.275] | 0.847 [0.695 - 1.033] | 1.006 [0.729 - 1.388] | 1.021 [0.861 - 1.212] | 1.033 [0.919 - 1.162] | 0.946 [0.715 - 1.252] | 1.106 [0.947 - 1.29] |
| Xhariep | 1.116 [0.857 - 1.454] | 1.105 [0.475 - 2.572] | 1.049 [0.728 - 1.511] | 1.218 [0.839 - 1.77] | 1.047 [0.766 - 1.43] | 1.213 [0.878 - 1.677] | 0.703 [0.434 - 1.139] | 0.873 [0.417 - 1.829] | 1.404 [0.929 - 2.122] | 1.116 [0.857 - 1.454] | 1.105 [0.475 - 2.572] | 1.049 [0.728 - 1.511] |
| Zululand | 0.976 [0.804 - 1.186] | 0.689 [0.439 - 1.082] | 1.055 [0.806 - 1.38] | 0.971 [0.748 - 1.261] | 1.057 [0.835 - 1.337] | 0.904 [0.726 - 1.125] | 1.075 [0.802 - 1.442] | 1.066 [0.655 - 1.735] | 0.821 [0.595 - 1.131] | 0.976 [0.804 - 1.186] | 0.689 [0.439 - 1.082] | 1.055 [0.806 - 1.38] |

**Table S10**. Relative risks of mortality and 95% confidence intervals associated with very long-term droughts (SPEI measured at fifteen months of accumulation, SPEI15) by district municipality in South Africa between 2009 and 2016. SPEI15 was associated with a larger and more robust estimates of all-cause mortality risk in the total population compared to other type of droughts.

| SPEI15 | all | <5 years | 5-24 years | 25-44 years | 45-64 years | 65-105 years | males | females | A00B99 | E00E90 | I00I99 | J00J99 |
| --- | --- | --- | --- | --- | --- | --- | --- | --- | --- | --- | --- | --- |
| Alfred Nzo | 0.957 [0.805 - 1.139] | 0.792 [0.537 - 1.169] | 0.932 [0.732 - 1.187] | 1.07 [0.85 - 1.346] | 0.944 [0.768 - 1.16] | 0.974 [0.8 - 1.186] | 1.155 [0.823 - 1.621] | 0.704 [0.373 - 1.326] | 0.911 [0.612 - 1.356] | 0.957 [0.805 - 1.139] | 0.792 [0.537 - 1.169] | 0.932 [0.732 - 1.187] |
| Amajuba | 1.072 [0.886 - 1.297] | 1.275 [0.791 - 2.056] | 1.18 [0.896 - 1.554] | 0.94 [0.714 - 1.237] | 1.038 [0.822 - 1.31] | 1.124 [0.906 - 1.394] | 1.068 [0.768 - 1.486] | 0.791 [0.492 - 1.271] | 1.26 [0.936 - 1.695] | 1.072 [0.886 - 1.297] | 1.275 [0.791 - 2.056] | 1.18 [0.896 - 1.554] |
| Amathole | 1.236 [1.079 - 1.417] | 1.553 [1.161 - 2.077] | 1.282 [1.083 - 1.519] | 1.148 [0.99 - 1.332] | 1.269 [1.089 - 1.48] | 1.204 [1.04 - 1.393] | 1.17 [0.967 - 1.415] | 1.143 [0.855 - 1.529] | 1.177 [0.984 - 1.409] | 1.236 [1.079 - 1.417] | 1.553 [1.161 - 2.077] | 1.282 [1.083 - 1.519] |
| Bojanala | 1.077 [0.911 - 1.274] | 1.015 [0.68 - 1.515] | 1.037 [0.848 - 1.267] | 1.168 [0.959 - 1.422] | 1.075 [0.888 - 1.301] | 1.078 [0.903 - 1.288] | 0.926 [0.736 - 1.166] | 1.28 [0.912 - 1.795] | 0.999 [0.811 - 1.23] | 1.077 [0.911 - 1.274] | 1.015 [0.68 - 1.515] | 1.037 [0.848 - 1.267] |
| Buffalo City | 1.229 [1.028 - 1.47] | 0.987 [0.656 - 1.486] | 1.269 [1.013 - 1.59] | 1.286 [1.029 - 1.607] | 1.302 [1.064 - 1.593] | 1.155 [0.943 - 1.415] | 1.225 [0.976 - 1.539] | 1.023 [0.636 - 1.645] | 1.478 [1.154 - 1.893] | 1.229 [1.028 - 1.47] | 0.987 [0.656 - 1.486] | 1.269 [1.013 - 1.59] |
| Cacadu | 1.153 [1.027 - 1.294] | 1.113 [0.824 - 1.503] | 1.167 [0.988 - 1.379] | 1.009 [0.856 - 1.189] | 1.2 [1.042 - 1.383] | 1.102 [0.963 - 1.261] | 1.151 [0.951 - 1.394] | 0.963 [0.691 - 1.342] | 1.222 [1.029 - 1.452] | 1.153 [1.027 - 1.294] | 1.113 [0.824 - 1.503] | 1.167 [0.988 - 1.379] |
| Cape Winelands | 1.109 [0.975 - 1.262] | 0.786 [0.521 - 1.186] | 1.23 [1.049 - 1.442] | 1.035 [0.882 - 1.213] | 1.16 [0.999 - 1.348] | 1.048 [0.897 - 1.225] | 1.219 [0.971 - 1.529] | 0.865 [0.548 - 1.365] | 1.02 [0.852 - 1.222] | 1.109 [0.975 - 1.262] | 0.786 [0.521 - 1.186] | 1.23 [1.049 - 1.442] |
| Capricorn | 1.172 [0.745 - 1.844] | 1.057 [0.369 - 3.027] | 1.458 [0.822 - 2.586] | 1.172 [0.704 - 1.952] | 1.136 [0.66 - 1.956] | 1.209 [0.747 - 1.957] | 1.05 [0.537 - 2.052] | 0.676 [0.295 - 1.549] | 1.661 [0.859 - 3.212] | 1.172 [0.745 - 1.844] | 1.057 [0.369 - 3.027] | 1.458 [0.822 - 2.586] |
| Central Karoo | 1.023 [0.875 - 1.196] | 1.454 [0.743 - 2.844] | 0.916 [0.714 - 1.176] | 1.074 [0.853 - 1.352] | 1.052 [0.859 - 1.288] | 0.991 [0.807 - 1.219] | 0.915 [0.636 - 1.317] | 1.366 [0.584 - 3.198] | 0.949 [0.721 - 1.247] | 1.023 [0.875 - 1.196] | 1.454 [0.743 - 2.844] | 0.916 [0.714 - 1.176] |
| Chris Hani | 1.167 [0.989 - 1.378] | 1.158 [0.789 - 1.699] | 1.162 [0.929 - 1.453] | 1.16 [0.956 - 1.409] | 1.178 [0.976 - 1.421] | 1.156 [0.962 - 1.39] | 1.026 [0.787 - 1.338] | 0.996 [0.683 - 1.453] | 1.347 [1.073 - 1.693] | 1.167 [0.989 - 1.378] | 1.158 [0.789 - 1.699] | 1.162 [0.929 - 1.453] |
| City of Cape Town | 1.05 [0.936 - 1.178] | 1.027 [0.814 - 1.298] | 1.048 [0.923 - 1.189] | 1.066 [0.946 - 1.2] | 1.029 [0.909 - 1.165] | 1.075 [0.954 - 1.211] | 1.016 [0.865 - 1.193] | 1.116 [0.872 - 1.428] | 1.092 [0.958 - 1.244] | 1.05 [0.936 - 1.178] | 1.027 [0.814 - 1.298] | 1.048 [0.923 - 1.189] |
| City of Johannesburg | 1.061 [0.919 - 1.225] | 0.97 [0.734 - 1.281] | 1.03 [0.878 - 1.208] | 1.071 [0.914 - 1.254] | 1.026 [0.879 - 1.197] | 1.104 [0.947 - 1.286] | 0.992 [0.824 - 1.193] | 0.979 [0.72 - 1.331] | 1.098 [0.928 - 1.3] | 1.061 [0.919 - 1.225] | 0.97 [0.734 - 1.281] | 1.03 [0.878 - 1.208] |
| City of Tshwane | 1.186 [0.972 - 1.448] | 1.244 [0.77 - 2.009] | 1.181 [0.933 - 1.494] | 1.117 [0.9 - 1.386] | 1.223 [0.987 - 1.515] | 1.14 [0.923 - 1.41] | 1.355 [1.007 - 1.823] | 1.27 [0.857 - 1.881] | 0.985 [0.779 - 1.246] | 1.186 [0.972 - 1.448] | 1.244 [0.77 - 2.009] | 1.181 [0.933 - 1.494] |
| Dr Kenneth Kaunda | 1.213 [0.99 - 1.487] | 1.835 [1.011 - 3.333] | 1.256 [0.957 - 1.65] | 1.233 [0.943 - 1.612] | 1.187 [0.938 - 1.502] | 1.243 [0.976 - 1.585] | 1.151 [0.84 - 1.578] | 1.366 [0.766 - 2.435] | 0.94 [0.682 - 1.295] | 1.213 [0.99 - 1.487] | 1.835 [1.011 - 3.333] | 1.256 [0.957 - 1.65] |
| Dr Ruth Segomotsi Mompati | 0.949 [0.752 - 1.199] | 1.064 [0.545 - 2.08] | 0.912 [0.658 - 1.264] | 1.087 [0.804 - 1.47] | 0.966 [0.74 - 1.261] | 0.924 [0.706 - 1.21] | 0.858 [0.587 - 1.252] | 0.835 [0.477 - 1.46] | 1.018 [0.706 - 1.467] | 0.949 [0.752 - 1.199] | 1.064 [0.545 - 2.08] | 0.912 [0.658 - 1.264] |
| Eden | 0.932 [0.841 - 1.033] | 0.775 [0.563 - 1.068] | 0.889 [0.773 - 1.022] | 1.01 [0.886 - 1.152] | 0.979 [0.865 - 1.107] | 0.879 [0.779 - 0.992] | 0.823 [0.67 - 1.012] | 1.157 [0.753 - 1.776] | 0.898 [0.769 - 1.049] | 0.932 [0.841 - 1.033] | 0.775 [0.563 - 1.068] | 0.889 [0.773 - 1.022] |
| Ehlanzeni | 1.028 [0.89 - 1.187] | 1.129 [0.828 - 1.539] | 1.079 [0.904 - 1.288] | 1.041 [0.882 - 1.229] | 1.093 [0.926 - 1.291] | 0.967 [0.83 - 1.127] | 1.049 [0.859 - 1.282] | 1.08 [0.829 - 1.408] | 1.058 [0.868 - 1.289] | 1.028 [0.89 - 1.187] | 1.129 [0.828 - 1.539] | 1.079 [0.904 - 1.288] |
| Ekurhuleni | 1.174 [1.008 - 1.367] | 1.237 [0.917 - 1.668] | 1.068 [0.894 - 1.276] | 1.282 [1.082 - 1.519] | 1.193 [1.01 - 1.409] | 1.162 [0.99 - 1.362] | 1.188 [0.963 - 1.465] | 1.208 [0.91 - 1.602] | 1.229 [1.021 - 1.479] | 1.174 [1.008 - 1.367] | 1.237 [0.917 - 1.668] | 1.068 [0.894 - 1.276] |
| eThekwini | 1.004 [0.863 - 1.168] | 0.785 [0.603 - 1.023] | 1.135 [0.958 - 1.345] | 0.956 [0.809 - 1.13] | 1.015 [0.856 - 1.204] | 0.994 [0.852 - 1.16] | 1.003 [0.826 - 1.218] | 1.001 [0.752 - 1.333] | 0.961 [0.81 - 1.141] | 1.004 [0.863 - 1.168] | 0.785 [0.603 - 1.023] | 1.135 [0.958 - 1.345] |
| Fezile Dabi | 1.105 [0.898 - 1.359] | 1.456 [0.738 - 2.873] | 1.055 [0.798 - 1.394] | 1.105 [0.85 - 1.436] | 1.218 [0.949 - 1.563] | 0.995 [0.781 - 1.267] | 0.992 [0.678 - 1.453] | 1.28 [0.764 - 2.144] | 1.036 [0.773 - 1.39] | 1.105 [0.898 - 1.359] | 1.456 [0.738 - 2.873] | 1.055 [0.798 - 1.394] |
| Frances Baard | 1.017 [0.767 - 1.349] | 0.409 [0.179 - 0.935] | 1.053 [0.722 - 1.536] | 1.193 [0.799 - 1.78] | 0.896 [0.643 - 1.249] | 1.187 [0.833 - 1.692] | 1.085 [0.683 - 1.724] | 2.407 [1.081 - 5.359] | 1.343 [0.85 - 2.123] | 1.017 [0.767 - 1.349] | 0.409 [0.179 - 0.935] | 1.053 [0.722 - 1.536] |
| Gert Sibande | 1.158 [0.943 - 1.422] | 0.932 [0.585 - 1.486] | 0.979 [0.754 - 1.273] | 1.494 [1.179 - 1.893] | 1.049 [0.832 - 1.321] | 1.292 [1.029 - 1.624] | 0.986 [0.727 - 1.337] | 1.183 [0.785 - 1.781] | 1.282 [0.954 - 1.723] | 1.158 [0.943 - 1.422] | 0.932 [0.585 - 1.486] | 0.979 [0.754 - 1.273] |
| Greater Sekhukhune | 1.178 [0.779 - 1.782] | 1.387 [0.5 - 3.847] | 1.178 [0.672 - 2.064] | 1.353 [0.848 - 2.157] | 1.215 [0.748 - 1.974] | 1.149 [0.726 - 1.818] | 1.227 [0.661 - 2.276] | 1.849 [0.794 - 4.309] | 1.029 [0.581 - 1.822] | 1.178 [0.779 - 1.782] | 1.387 [0.5 - 3.847] | 1.178 [0.672 - 2.064] |
| iLembe | 1.229 [0.958 - 1.575] | 1.042 [0.559 - 1.945] | 1.271 [0.905 - 1.787] | 1.355 [0.989 - 1.857] | 1.076 [0.803 - 1.441] | 1.4 [1.043 - 1.879] | 1.264 [0.866 - 1.846] | 0.861 [0.487 - 1.521] | 1.58 [1.11 - 2.249] | 1.229 [0.958 - 1.575] | 1.042 [0.559 - 1.945] | 1.271 [0.905 - 1.787] |
| Joe Gqabi | 1.224 [0.981 - 1.529] | 0.768 [0.412 - 1.431] | 1.434 [1.043 - 1.971] | 1.276 [0.964 - 1.69] | 1.221 [0.945 - 1.579] | 1.229 [0.943 - 1.604] | 0.884 [0.598 - 1.307] | 1.373 [0.678 - 2.781] | 1.197 [0.811 - 1.767] | 1.224 [0.981 - 1.529] | 0.768 [0.412 - 1.431] | 1.434 [1.043 - 1.971] |
| John Taolo Gaetsewe | 1.126 [0.831 - 1.525] | 2.442 [1.067 - 5.589] | 1.17 [0.778 - 1.759] | 1.093 [0.696 - 1.715] | 0.93 [0.661 - 1.309] | 1.391 [0.951 - 2.036] | 1.107 [0.657 - 1.865] | 0.419 [0.158 - 1.114] | 0.995 [0.589 - 1.678] | 1.126 [0.831 - 1.525] | 2.442 [1.067 - 5.589] | 1.17 [0.778 - 1.759] |
| Lejweleputswa | 1.234 [1.004 - 1.517] | 1.268 [0.744 - 2.16] | 1.162 [0.909 - 1.485] | 1.215 [0.944 - 1.564] | 1.15 [0.909 - 1.454] | 1.332 [1.051 - 1.688] | 1.05 [0.734 - 1.502] | 1.022 [0.652 - 1.602] | 1.157 [0.85 - 1.574] | 1.234 [1.004 - 1.517] | 1.268 [0.744 - 2.16] | 1.162 [0.909 - 1.485] |
| Mangaung | 1.025 [0.819 - 1.283] | 0.869 [0.498 - 1.515] | 0.99 [0.739 - 1.327] | 1.024 [0.777 - 1.349] | 0.992 [0.772 - 1.275] | 1.067 [0.825 - 1.381] | 0.971 [0.686 - 1.375] | 1.29 [0.786 - 2.118] | 0.899 [0.645 - 1.253] | 1.025 [0.819 - 1.283] | 0.869 [0.498 - 1.515] | 0.99 [0.739 - 1.327] |
| Mopani | 1.132 [0.931 - 1.377] | 1.524 [0.974 - 2.384] | 0.921 [0.699 - 1.213] | 1.333 [1.064 - 1.67] | 1.137 [0.902 - 1.432] | 1.131 [0.914 - 1.398] | 0.984 [0.727 - 1.332] | 1.203 [0.817 - 1.771] | 1.096 [0.808 - 1.487] | 1.132 [0.931 - 1.377] | 1.524 [0.974 - 2.384] | 0.921 [0.699 - 1.213] |
| Namakwa | 1.343 [1.026 - 1.757] | 0.794 [0.22 - 2.867] | 1.084 [0.723 - 1.625] | 1.412 [0.971 - 2.055] | 1.361 [0.958 - 1.935] | 1.316 [0.921 - 1.878] | 0.838 [0.388 - 1.812] | 1.052 [0.349 - 3.177] | 1.599 [1.014 - 2.522] | 1.343 [1.026 - 1.757] | 0.794 [0.22 - 2.867] | 1.084 [0.723 - 1.625] |
| Nelson Mandela Bay | 0.923 [0.769 - 1.107] | 0.883 [0.632 - 1.234] | 0.916 [0.742 - 1.13] | 0.956 [0.784 - 1.165] | 0.923 [0.761 - 1.121] | 0.921 [0.761 - 1.114] | 0.941 [0.757 - 1.17] | 0.832 [0.573 - 1.207] | 1.017 [0.824 - 1.255] | 0.923 [0.769 - 1.107] | 0.883 [0.632 - 1.234] | 0.916 [0.742 - 1.13] |
| Ngaka Modiri Molema | 1.212 [1.009 - 1.456] | 1.002 [0.621 - 1.616] | 1.091 [0.867 - 1.372] | 1.303 [1.039 - 1.634] | 1.128 [0.911 - 1.398] | 1.311 [1.075 - 1.598] | 1.065 [0.8 - 1.418] | 1.404 [0.921 - 2.142] | 1.21 [0.935 - 1.565] | 1.212 [1.009 - 1.456] | 1.002 [0.621 - 1.616] | 1.091 [0.867 - 1.372] |
| Nkangala | 1.305 [1.015 - 1.678] | 2.696 [1.399 - 5.197] | 1.158 [0.839 - 1.597] | 1.402 [1.037 - 1.896] | 1.481 [1.102 - 1.991] | 1.132 [0.858 - 1.493] | 1.172 [0.784 - 1.751] | 1.099 [0.639 - 1.89] | 1.317 [0.915 - 1.896] | 1.305 [1.015 - 1.678] | 2.696 [1.399 - 5.197] | 1.158 [0.839 - 1.597] |
| O.R.Tambo | 1.132 [0.997 - 1.286] | 1.344 [1.066 - 1.695] | 1.102 [0.939 - 1.292] | 1.038 [0.895 - 1.203] | 1.095 [0.949 - 1.264] | 1.173 [1.025 - 1.344] | 1.115 [0.931 - 1.335] | 1.215 [0.873 - 1.691] | 1.1 [0.889 - 1.36] | 1.132 [0.997 - 1.286] | 1.344 [1.066 - 1.695] | 1.102 [0.939 - 1.292] |
| Overberg | 1.157 [0.994 - 1.347] | 1.157 [0.655 - 2.044] | 1.177 [0.946 - 1.465] | 1.149 [0.94 - 1.405] | 1.173 [0.97 - 1.419] | 1.144 [0.938 - 1.395] | 1.09 [0.76 - 1.565] | 0.732 [0.394 - 1.361] | 1.158 [0.917 - 1.462] | 1.157 [0.994 - 1.347] | 1.157 [0.655 - 2.044] | 1.177 [0.946 - 1.465] |
| Pixley ka Seme | 1.186 [0.991 - 1.418] | 1.271 [0.725 - 2.227] | 1.159 [0.903 - 1.487] | 1.116 [0.877 - 1.42] | 1.141 [0.932 - 1.397] | 1.23 [0.987 - 1.534] | 1.185 [0.855 - 1.641] | 1.337 [0.795 - 2.25] | 1.019 [0.783 - 1.326] | 1.186 [0.991 - 1.418] | 1.271 [0.725 - 2.227] | 1.159 [0.903 - 1.487] |
| Sedibeng | 1.079 [0.916 - 1.271] | 0.963 [0.651 - 1.426] | 1.135 [0.928 - 1.389] | 1.162 [0.949 - 1.423] | 1.073 [0.892 - 1.291] | 1.085 [0.899 - 1.309] | 0.943 [0.732 - 1.215] | 0.996 [0.665 - 1.492] | 1.349 [1.08 - 1.686] | 1.079 [0.916 - 1.271] | 0.963 [0.651 - 1.426] | 1.135 [0.928 - 1.389] |
| Sisonke | 1.264 [1.043 - 1.532] | 1.098 [0.714 - 1.688] | 1.102 [0.842 - 1.443] | 1.139 [0.878 - 1.477] | 1.387 [1.115 - 1.726] | 1.158 [0.918 - 1.46] | 1.126 [0.831 - 1.527] | 1.529 [0.957 - 2.442] | 1.151 [0.838 - 1.579] | 1.264 [1.043 - 1.532] | 1.098 [0.714 - 1.688] | 1.102 [0.842 - 1.443] |
| Siyanda | 0.875 [0.636 - 1.205] | 0.557 [0.186 - 1.664] | 0.813 [0.514 - 1.283] | 0.865 [0.556 - 1.346] | 0.943 [0.644 - 1.381] | 0.8 [0.541 - 1.184] | 0.925 [0.493 - 1.733] | 0.558 [0.185 - 1.687] | 0.956 [0.578 - 1.583] | 0.875 [0.636 - 1.205] | 0.557 [0.186 - 1.664] | 0.813 [0.514 - 1.283] |
| Thabo Mofutsanyane | 1.114 [0.914 - 1.356] | 1.779 [1.118 - 2.834] | 1.114 [0.872 - 1.423] | 1.084 [0.846 - 1.388] | 1.069 [0.849 - 1.345] | 1.153 [0.931 - 1.427] | 1.085 [0.804 - 1.465] | 1.096 [0.715 - 1.68] | 1.286 [0.982 - 1.683] | 1.114 [0.914 - 1.356] | 1.779 [1.118 - 2.834] | 1.114 [0.872 - 1.423] |
| Ugu | 1.055 [0.895 - 1.244] | 1.085 [0.764 - 1.541] | 1.063 [0.848 - 1.332] | 0.987 [0.806 - 1.209] | 1.104 [0.912 - 1.336] | 1.007 [0.843 - 1.205] | 1.042 [0.833 - 1.303] | 1.096 [0.755 - 1.592] | 0.958 [0.759 - 1.21] | 1.055 [0.895 - 1.244] | 1.085 [0.764 - 1.541] | 1.063 [0.848 - 1.332] |
| UMgungundlovu | 1.021 [0.86 - 1.212] | 1.409 [0.938 - 2.117] | 0.912 [0.738 - 1.128] | 1.049 [0.851 - 1.291] | 1.041 [0.847 - 1.279] | 0.993 [0.825 - 1.195] | 0.972 [0.734 - 1.288] | 1.039 [0.704 - 1.534] | 1.066 [0.859 - 1.324] | 1.021 [0.86 - 1.212] | 1.409 [0.938 - 2.117] | 0.912 [0.738 - 1.128] |
| Umkhanyakude | 1.193 [0.816 - 1.745] | 2.889 [1.076 - 7.761] | 1.402 [0.795 - 2.475] | 1.109 [0.67 - 1.834] | 1.117 [0.71 - 1.757] | 1.288 [0.826 - 2.006] | 0.833 [0.468 - 1.482] | 1.111 [0.364 - 3.393] | 1.713 [0.927 - 3.166] | 1.193 [0.816 - 1.745] | 2.889 [1.076 - 7.761] | 1.402 [0.795 - 2.475] |
| Umzinyathi | 1.245 [1 - 1.549] | 1.362 [0.769 - 2.414] | 1.462 [1.054 - 2.03] | 1.216 [0.921 - 1.606] | 1.255 [0.965 - 1.633] | 1.241 [0.967 - 1.594] | 1.04 [0.709 - 1.527] | 1.117 [0.632 - 1.973] | 1.058 [0.758 - 1.476] | 1.245 [1 - 1.549] | 1.362 [0.769 - 2.414] | 1.462 [1.054 - 2.03] |
| Uthukela | 1.068 [0.874 - 1.306] | 1.48 [0.905 - 2.42] | 0.947 [0.711 - 1.261] | 0.998 [0.776 - 1.282] | 1.027 [0.812 - 1.3] | 1.113 [0.881 - 1.405] | 1.055 [0.792 - 1.406] | 1.081 [0.668 - 1.751] | 0.992 [0.732 - 1.344] | 1.068 [0.874 - 1.306] | 1.48 [0.905 - 2.42] | 0.947 [0.711 - 1.261] |
| Uthungulu | 1.025 [0.834 - 1.26] | 0.947 [0.563 - 1.592] | 1.083 [0.827 - 1.416] | 1.12 [0.874 - 1.435] | 0.98 [0.766 - 1.255] | 1.081 [0.858 - 1.361] | 1.128 [0.81 - 1.571] | 0.907 [0.544 - 1.513] | 1.095 [0.785 - 1.526] | 1.025 [0.834 - 1.26] | 0.947 [0.563 - 1.592] | 1.083 [0.827 - 1.416] |
| Vhembe | 1.004 [0.732 - 1.378] | 0.549 [0.245 - 1.228] | 1.193 [0.778 - 1.829] | 1.006 [0.703 - 1.442] | 0.927 [0.635 - 1.354] | 1.079 [0.76 - 1.532] | 0.923 [0.509 - 1.673] | 0.611 [0.322 - 1.16] | 0.942 [0.553 - 1.604] | 1.004 [0.732 - 1.378] | 0.549 [0.245 - 1.228] | 1.193 [0.778 - 1.829] |
| Waterberg | 1.167 [0.844 - 1.614] | 1.456 [0.576 - 3.685] | 1.166 [0.732 - 1.859] | 0.993 [0.658 - 1.5] | 1.368 [0.93 - 2.013] | 0.996 [0.685 - 1.448] | 0.81 [0.473 - 1.387] | 1.01 [0.453 - 2.251] | 0.93 [0.575 - 1.505] | 1.167 [0.844 - 1.614] | 1.456 [0.576 - 3.685] | 1.166 [0.732 - 1.859] |
| West Coast | 0.93 [0.745 - 1.16] | 2.157 [0.972 - 4.788] | 0.983 [0.712 - 1.357] | 0.769 [0.592 - 0.999] | 0.848 [0.653 - 1.103] | 1.05 [0.8 - 1.379] | 0.978 [0.625 - 1.531] | 2.042 [0.892 - 4.674] | 0.759 [0.55 - 1.047] | 0.93 [0.745 - 1.16] | 2.157 [0.972 - 4.788] | 0.983 [0.712 - 1.357] |
| West Rand | 0.989 [0.852 - 1.149] | 0.862 [0.599 - 1.242] | 1.049 [0.861 - 1.277] | 0.937 [0.768 - 1.143] | 0.926 [0.786 - 1.09] | 1.065 [0.895 - 1.266] | 0.809 [0.628 - 1.042] | 0.946 [0.629 - 1.423] | 0.937 [0.753 - 1.165] | 0.989 [0.852 - 1.149] | 0.862 [0.599 - 1.242] | 1.049 [0.861 - 1.277] |
| Xhariep | 1.312 [0.972 - 1.769] | 1.231 [0.457 - 3.315] | 1.35 [0.891 - 2.046] | 1.36 [0.897 - 2.062] | 1.294 [0.906 - 1.848] | 1.319 [0.916 - 1.9] | 0.732 [0.414 - 1.296] | 0.95 [0.401 - 2.247] | 1.615 [1.021 - 2.555] | 1.312 [0.972 - 1.769] | 1.231 [0.457 - 3.315] | 1.35 [0.891 - 2.046] |
| Zululand | 1.078 [0.78 - 1.489] | 0.625 [0.285 - 1.369] | 1.354 [0.873 - 2.099] | 1.264 [0.835 - 1.913] | 1.182 [0.8 - 1.747] | 0.989 [0.687 - 1.426] | 0.859 [0.515 - 1.432] | 1.328 [0.594 - 2.97] | 0.911 [0.545 - 1.522] | 1.078 [0.78 - 1.489] | 0.625 [0.285 - 1.369] | 1.354 [0.873 - 2.099] |
